# Supplementary material for: Spatiotemporal analysis of glioma heterogeneity reveals COL1A1 as an actionable target to disrupt tumor progression
Source: Nat Commun. 2022 Jun 24;13:3606. doi: 10.1038/s41467-022-31340-1 (PMC9232499; doi:10.1038/s41467-022-31340-1)
Supplement: Supplementary file 1 — Supplementary Information [file 41467_2022_31340_MOESM1_ESM.pdf]

Supplementary Information

Spatiotemporal analysis of glioma heterogeneity reveals Col1A1 as an actionable target to disrupt tumor progression

Andrea Comba, Syed M. Faisal, Patrick J. Dunn, Anna E. Argento, Todd C. Hollon, Wajd N. Al-Holou, Maria Luisa Varela, Daniel B. Zamler, Gunnar L Quass, Pierre F. Apostolides, Clifford Abel II, Christine E. Brown, Phillip E. Kish, Alon Kahana, Celina G. Kleer, Sebastien Motsch, Maria G Castro, Pedro R. Lowenstein, *

*Correspondence to: pedrol@umich.edu

This file includes:

Supplementary Methods

Supplementary Figures S1-S37

Supplementary Tables S1, S2, S3, S4, S6, S6, S7

Other Supplementary Data Files:

Movies #1 to #17

Supplementary Data 1

Supplementary Data 2

SUPPLEMENTARY METHODS

Craniotomy and cranial window implantation

Mice were anesthetized using an intraperitoneal injection of injectable anesthetics ketamine and dexmedetomidine. One dose of carprofen was given pre-operatively, and another dose was given 24hr post-surgery. Eye ointment was applied prior to surgery, and hairs overlying the skull were clipped with a hair-clipper. Following anesthesia induction, the mouse was placed in a stereotactic apparatus and betadine disinfectant was applied to the incision site. The skin was cut, retracted and periosteum, as well as musculature on the surface of the skull, was gently scraped to ensure entire cranium dry and rough. Then, a craniotomy was made over the right hemisphere in between the bregma and lambda (size, 3x3 mm; center relative to Bregma: lateral right, 0.5mm; posterior, 0.5mm). Multiple rounds of slow and delicate drilling were performed to ensure that underlying brain matter did not overheat. The skull was moistened with chilled sterile saline solution in between rounds of drilling to further reduce heat and swelling. Once the edges of the craniotomy are thin enough, the skull fragment in a single piece was then removed using fine forceps or a 26G needle. Intracranial injection of $5x10^4$ GFP^+ NPA or NPAshCOL1A1 glioma cells were implanted at a depth of 0.8mm ventral through the craniotomy overlying the brain cortex of B6.129(Cg)-Gt(ROSA)26Sortm4(ACTB-tdTomato-EGFP)Luo/J using a Hamilton syringe in a volume of 1ul over the period of 5min. These tdTomato mice were utilized to identify (in red) normal brain tissue, and thus establish tumor borders.

Following the injection, imaging window was constructed from two layers of standard microscope cover glass (Electron Microscopy Sciences), of which one was small (3mm) equal to the size of the craniotomy and another was large (5mm), which were joined with an ultraviolet curable optical adhesive (NOR-71, Norland). Two layered coverslip was carefully placed over the exposed brain filled with saline to facilitate visual access to tumor and brain during the imaging. A larger piece was attached to the cranium and a smaller insert was fitted snugly into the craniotomy. The cyanoacrylate-based adhesive (Krazy glue) was spread with a needle towards the edges of the larger coverslip to avoid leeching of the glue underneath of the coverslip obscuring the brain. To permanently secure the coverslip on the skull, the coverslip was held in place by gentle pressure from a blunt

needle of the syringe. A thin layer of glue was then applied over the entire surface of the exposed skull except for the location of the craniotomy. Once the adhesive was set, the blunt needle was slowly lifted away, and the coverslip was further reinforced to the skull with dental cement. A lightweight, metallic headbar was positioned on the skull posterior to the cranial window and affixed using dental cement to stabilize the imaging plane and minimize motion artifacts during the time-lapse imaging. A dental acrylic well was created around the cranial window to immerse the imaging objective. Upon hardening of the dental cement, the animal was removed from and allowed to recover on a heating pad in its home cage while under constant supervision. The animal was placed in a new home cage with only one animal per cage and allowed to recover. The animals were recovered from anesthesia using Atipamezole via intra-peritoneal injection (1.0 mg/Kg) to reverse Ketamine/Xylazine, and a single subcutaneous injection of Buprenorphine (0.1mg/kg subcutaneous) was administered as post-operative pain relief. Water-soaked chow in ceramic crock was placed on the cage floor post-op. The following day the animal was received a second dose of carprofen for pain relief.

Two photon intravital live imaging *in vivo*:

One-week NPA tumor cells' injection and cranial window implantation, and two-weeks post NPAshCOL1A1 implantation, mice were monitored for tumor growth using an IVIS bioluminescence imaging system. Mice with positive bioluminescent signal were placed in the glass jar and anesthesia was induced using isoflurane (3%). After anesthesia was achieved, mouse was transported to the multiphoton imaging platform and held in place using the headbar and headbar clamp. The breadboard (Thorlabs) was fitted with a nose cone for applying inhalational isoflurane anesthesia while the mouse was placed in the headbar clamp. Water base immersion oil was used to fill the dental acrylic well and the objective of the microscope was lowered towards the cranial window until it was situated just beneath the surface layer of oil. The animal was placed on a heating pad to maintain physiologic body temperature regulated by a rectal thermal probe (Stoelting), under continuous light anesthesia with isoflurane (1%). Dehydration was prevented by giving 300μl isotonic saline every three hours using a catheter placed subcutaneously. Eyes were lubricated with eye-ointment to prevent drying while under anesthesia. The vitals of

the mice were monitored using a mouse thigh sensor with MouseOX Plus (Star Life Sciences Corp). To visualize glioma dynamics in real-time in the living animal with a Bruker Optima two-photon microscope, using a Chameleon Ti-sapphire laser (Coherent) tuned to 920 nm and attenuated with a Pockels cell to adjust the power at the sample so that approximately the same signal level could be achieved at a constant frame rate starting from 150mW for the surface to 300mW at imaging depths of 300-330 µm. The images were acquired with a 20X water immersion objective (Olympus, NA 1.0). To avoid imaging at the brain surface, first we acquired high-resolution 3D z-stacks spanning 0-330 µm depth from the surface of the brain. Z-stacks were then imported to Imaris viewer version 9.8 (Bitplane, Imaris, Oxford Instruments, MA, USA) to obtain 3D images. We then used Orthoslicer3D to reveal the depth of the imaging position in relation to the surface of the brain for time-lapse data acquisition. Images within the cortex were taken of the GFP+ tumor (peak excitation: 700nm) and TdTomato brain (peak excitation: 920nm) every 5min for up to 8-12 hours. Emitted fluorescence was split with a dichroic mirror to separate green (GFP) and red (Tdtomato) channels. Once imaging was complete, animals were removed from the stereotactic frame and allowed to recover in a clean cage under a heating lamp.

Mathematical analysis of tumor cell movement

We used as parameters for the cell size (called 'blob') 20µm and a threshold of 1 together with the DoG method (Difference of Gaussian detectors). We then obtained for each experiment several paths for many different cells. To reduce the erratic behavior, we filtered the trajectories. From the data obtained with TrackMate, we then smoothed out the paths which allowed to estimate the velocity of each cell. We simply use a Gaussian Kernel as a filter (with standard deviation $\sigma = 2$ and a stencil of 9 points). Thus, we had an estimation of the positions $x_i(t)$ and velocities $v_i(t)$ of the cells i. From the velocity $v_i(t)$, we also deduced the velocity direction θ_i (t).

After obtaining these parameters we applied several statistics to investigate cell behavior. Each cell is characterized by a position x_i Є R2 and a velocity v_i Є R2. The velocity vector v can be decomposed into a speed (scalar) c = |v| and angle direction θ such that v = c (cos θ, sin θ). We utilized three statistics to visualize the distribution of velocity: (i)

distribution of velocity angle θ (provides indication of the overall direction of the cells); (ii) distribution of speed c and (iii) distribution of the velocity vector v = (vx, vy). Since v is a vector, we need to plot a 'heatmap' to visualize its distribution.

To understand the organization of oncostream dynamics we analyzed the cell-cell correlation using two further statistics using pair wise information: (i) Neighbor density: taking a reference cell xi, we estimate the probability to have another cell x_j nearby, and, (ii) velocity correlation: depending on the position of a nearby x_j, estimate the correlation: $\omega_i \cdot \omega_j$ where ω_i (resp. ω_j) is the (normalized) velocity direction of cell *i* (resp. $_j$). A correlation of +1/-1 indicates that cells are moving in the same/opposite direction, whereas 0 indicates that they move in orthogonal direction. Orthogonal direction is when $\omega_i \cdot \omega_j = 0$. Details on how to estimate numerically the correlation functions are described in **Fig. S17 A, B, C**.

Classification of glioma migration patterns

Our data-set consists of cell orientations $\theta_n \in [0, 2\pi)$ with $n = 1..N$ indexing the cells and N being the total number of cells in a given zone. Zones were defined by either varying density, alignment, geographical distribution, and/or movement patterns of cells for each experiment. We build a criteria to decide whether the underlying distribution of orientations θ is *more like* a flock, a stream or a swarm. With this aim, we define three types of distributions ρ_{flock}, ρ_{stream} and ρ_{swarm} and use *likelihood* estimation to determine which distribution is more likely (given the data-set $\{\theta_n\}_{n=1..N}$). First, we present the three distributions used to describe a flock, a stream and a swarm.

Flock: wrapped normal distribution. In a flock, the distribution of orientations should be *uni-modal*, i.e. the orientations are centered around a given direction denoted $\overline{\theta}$. Thus, we use for ρ_{flock} a *wrapped* Gaussian distribution:

$$\rho_{flock}(\theta;\ \overline{\theta}, \sigma) = \frac{1}{\sigma\sqrt{2\pi}} \sum_{k=-\infty}^{\infty} {}^{-\frac{|\theta-\overline{\theta}+2\pi k|^2}{2\sigma^2}}, \qquad (1)$$

where $\overline{\theta}$ represents the 'peak' of the distribution and σ measures its spreading. Summing in *k* is necessary to ensure that the distribution ρ_{flock} is 2π periodic in θ. The two free

parameters $\overline{\theta}$ and σ of the distribution are determined by maximizing the *likelihood*, i.e. by determining $\overline{\theta}_*$ and σ_* the maximizers of the following quantity:

$$\overline{\theta}_*, \sigma_* = \mathrm{argmax}_{\overline{\theta},\sigma}\mathcal{L}(\overline{\theta},\sigma) \quad \text{with} \quad \mathcal{L}(\overline{\theta},\sigma) = \prod_{n=1}^{N} \rho_{flock}(\theta_n;\ \overline{\theta},\sigma). \tag{2}$$

After some standard computations (taking the log), we find an explicit solution:

$$\overline{\theta}_* = \mathrm{Arg}(\bar{\mathbf{z}}) \quad , \quad \sigma_*^2 = -\ln R_e^2$$

with $\bar{\mathbf{z}} = \frac{1}{N}\sum_{n=1}^{N} {}^{i\theta_n}$ (using complex number) and $R_e^2 = \frac{N}{N-1}\left(|\bar{\mathbf{z}}|^2 - \frac{1}{N}\right)$.

Stream: symmetric wrapped normal distribution. In a stream, the distribution of orientations is only π periodic since the distribution of cells moving in both directions inside the stream should be alike. Thus, we use for ρ_{stream} a symmetric version of ρ_{flock}:

$$\rho_{stream}(\theta;\ \overline{\theta},\sigma) = \frac{1}{2}\Big(\rho_{flock}(\theta;\ \overline{\theta},\sigma) + \rho_{flock}(\theta+\pi;\ \overline{\theta},\sigma)\Big) \tag{3}$$

where $\rho_{flock}(\theta;\ \overline{\theta},\sigma)$ is defined in (Eq. 1). The estimation of the two free parameters $\overline{\theta}$ and σ can be estimated as previously by maximizing the likelihood. We notice for that the following identity:

$$\begin{aligned}
\rho_{stream}(\theta;\ \overline{\theta},\sigma) &= \frac{1}{2\sigma\sqrt{2\pi}} \sum_{k=-\infty}^{\infty} \left(^{-\frac{|\theta-\overline{\theta}+2\pi k|^2}{2\sigma^2}} + ^{-\frac{|\theta-\overline{\theta}+2\pi k+2\pi|^2}{2\sigma^2}} \right) \\
&= \frac{1}{2\sigma\sqrt{2\pi}} \sum_{k=-\infty}^{\infty} \left(^{-\frac{|2\theta-2\overline{\theta}+4\pi k|^2}{2(2\sigma)^2}} + ^{-\frac{|2\theta-2\overline{\theta}+4\pi k+2\pi|^2}{2(2\sigma)^2}} \right) \\
&= \frac{1}{2\sigma\sqrt{2\pi}} \sum_{k=-\infty}^{\infty} {}^{-\frac{|2\theta-2\overline{\theta}+2\pi k|^2}{2(2\sigma)^2}} = \rho_{flock}(2\theta;\ 2\overline{\theta}, 2\sigma).
\end{aligned} \tag{4}$$

Thus, the parameters $\overline{\theta}_*$ and σ_* for the stream distribution are estimated using with $\bar{\mathbf{z}}_{\mathbf{2}} = \frac{1}{N}\sum_{n=1}^{N} {}^{i2\theta_n}$ in lieu of $\bar{\mathbf{z}}$.

Swarm: uniform distribution. In a swarm, the orientations of the cells are 'random' meaning that all directions are equally probable. Thus, the distribution ρ_{swarm} is simply constant:

$$\rho_{swarm}(\theta) = \frac{1}{2\pi}.$$

There is no free parameters. The normalization 2π is to make sure that the distribution satisfies $\int_0^{2\pi} \rho_{swarm}(\theta)\,\mathrm{d}\theta = 1$.

Model selection. Since we have now three distributions ρ_{flock}, ρ_{stream} and ρ_{swarm}, we can determine the likelihood of our data-set $\{\theta_n\}_{n=1..N}$ in each case. The higher the likelihood, the higher the chance the model (flock, stream or flock) is selected. However, this strategy will never select a swarm since there is no free parameter in ρ_{swarm} and a flock or stream distribution can be made uniform with σ large. One could penalize having free parameters in ρ_{flock} and ρ_{stream} using information criterion (e.g. AIC, BIC) but even then swarm distributions are not selected, a flock or a stream will always be selected.

For this reason, we *fix* a priori the value of σ for the flock and stream distribution ρ_{flock} and ρ_{stream}. In other words, a distribution will be considered a flock if the distribution is not "too" flat. We choose:

$$\sigma^2_{flock} = 2 \quad , \quad \sigma^2_{stream} = 1. \tag{5}$$

We give an illustration with the experiment "scene 5" zone D in figure 3. The associated log-likelihood gives:

$$\ln(\mathcal{L}_{flock}) = -46798.93, \;\; \ln(\mathcal{L}_{stream}) = -43583.16, \;\; \ln(\mathcal{L}_{swarm}) = -43732.28. \tag{6}$$

Thus, in this example, we select a stream formation since the log-likelihood is the highest.

Analysis of the tumor borders

Phase separation. We denote $h(x,y)$ the original image of green intensity where (x,y) is a position (pixel position) and $h(x,y) \in [0,1]$ the intensity of green color (i.e. 1 indicate a tumor, 0 no tumor). We want to separate the image into two regions: inside the tumor ("+1") and outside ("-1").

To do so, we define a threshold $h_* = .12$ and then apply the Allen-Cahn equation to the function $c_0(x,y) = h(x,y) - h_*$:

$$\partial_t c = c(1 - c^2) + \gamma \Delta c \qquad (7)$$

where $\gamma = .5$ is the diffusion parameter to *smooth* the distribution. The source term ``$c(1 - c^2)$" makes the function $c(x,y,t)$ converge to either -1 or $+1$. The dynamics (7) can be interpreted as a gradient flow with respect to the energy:

$$\mathcal{E}[c] = \int_{\mathbb{R}^2} \frac{1}{4}(c^2 - 1)^2 + \frac{\gamma}{2}|\nabla c|^2 \, \mathrm{d}\mathbf{x}. \qquad (8)$$

We present in Supplementary Figure S28 the evolution of the $c(x,y,t)$ over time, transitioning from a representative original image (in red/blue scale instead of green) into a *binary* image with only two values -1 and $+1$.

Statistic of the border. Once the image has been split into two regions (using Allen-Cahn equation), we investigate the sinuosity of the border. The sinuosity is defined as the ratio between the length of the curve *L* and the distance joining the end points of the curve (see Figure 8H-I). The more 'sinuous' a curve is, the large the sinuosity. We create a boxplot computing the sinuosity for all the experiments regrouping the experiments with/without collagen.

Supplementary Tables

Supplementary Table 1. Analysis of oncostreams on TCGA glioma diagnostic slides from the Genomic Data Commons Portal

TCGA Manual analysis				
**Grade**	**Recurrence**	**Total Tumors**	**OS Positive**	**OS Negative**
**GBM-Grade IV**	Primary	100	**47**	53
**LGG-Grade III**	Primary	70	**6**	64
**LGG-Grade II**	Primary	50	**0**	50

Supplementary Table 2. Summary results of oncostreams detection using manually histopathological analysis and deep learning segmentation

Accuracy of methodologies for Oncostreams analysis					
Tumor	Method	Oncostreams		Concordance	
		Positive	Negative	Images #	Percentage (%)
GBM - Grade IV	Manually	77	32	88/109	80.7
	Deep Learning	78	31		
LGG - Grade III	Manually	29	97	113/126	89.7
	Deep Learning	24	102		
LGG - Grade II	Manually	0	61	60/61	98.4
	Deep Learning	1	60		

Supplementary Table 3. Enriched Gene Ontologies (GOs) biological process within oncostream fascicles

GO ID	Go Name	countDE	countAll	pv_fdr
GO:0030335	positive regulation of cell migration	9	561	0.000845
GO:2000147	positive regulation of cell motility	9	584	0.000845
GO:0040017	positive regulation of locomotion	9	600	0.000845
GO:0051272	positive regulation of cellular component movement	9	604	0.000845
GO:0030334	regulation of cell migration	10	932	0.002304
GO:2000145	regulation of cell motility	10	982	0.002731
GO:0040012	regulation of locomotion	10	1029	0.003686
GO:0051270	regulation of cellular component movement	10	1072	0.004748
GO:0006950	response to stress	18	3567	0.006971
GO:0032963	collagen metabolic process	4	111	0.007461
GO:0016477	cell migration	11	1488	0.011264
GO:0040011	locomotion	12	1842	0.01536
GO:0048870	cell motility	11	1647	0.022187
GO:0051674	localization of cell	11	1647	0.022187
GO:0051240	positive regulation of multicellular organismal process	12	1973	0.022674
GO:0030198	extracellular matrix organization	5	301	0.022674
GO:0043062	extracellular structure organization	5	302	0.022674
GO:0009605	response to external stimulus	14	2673	0.027927
GO:0006952	defense response	10	1521	0.040737

Supplementary Table 4. Pathways of the Network for COL1A1

Pathways of the Network including Col1a1	p-value	FDR	Genes	minusLOGFDR
Relaxin signaling pathway(K)	2.96E-07	2.52E-05	Mapk3,Jun,Fos,Mmp9,Nfkb1,Col1a1,Acta2	4.598823558
AGE-RAGE signaling pathway in diabetic complications(K)	2.31E-05	3.99E-04	Mapk3,Jun,Stat3,Nfkb1,Col1a1	3.398907391
Focal adhesion(K)	5.91E-04	3.55E-03	Fyn,Mapk3,Jun,Col1a1,Ctnnb1	2.450138812
Human papillomavirus infection(K)	1.01E-03	5.07E-03	Hdac2,Mapk3,Isg15,Nfkb1,Col1a1,Ctnnb1	2.29537768
Extracellular matrix organization(R)	2.00E-03	7.99E-03	Adamts2,Mmp9,Mmp10,Col1a1,Tll1	2.097529324
RNA Polymerase II Transcription(R)	2.69E-03	9.98E-03	Hdac2,Irak1,Ubc,Mapk3,Jun,Fos,Col1a1,Sp1,Ctnnb1	2.00075198
VEGFR3 signaling in lymphatic endothelium(N)	3.43E-03	0.0103	Mapk3,Col1a1	1.987162775
Platelet activation(K)	8.79E-03	0.0213	Fyn,Mapk3,Col1a1	1.671620397
Beta3 integrin cell surface interactions(N)	9.77E-03	0.0213	Cyr61,Col1a1	1.671620397
Integrin signalling pathway(P)	0.0172	0.0343	Fyn,Mapk3,Col1a1	1.46470588
IL4-mediated signaling events(N)	0.0207	0.0414	Col1a1,Sp1	1.382999659
Amoebiasis(K)	0.0434	0.0472	Nfkb1,Col1a1	1.326058001

Supplementary Table 5. Pathways of the Modules

Module	GeneSet	FDR	Nodes	NegLOGFDR
M1	Alzheimer disease-presenilin pathway(P)	0.0461	Acta2,Mmp9	1.336299075
	Leukocyte transendothelial migration(K)	0.0461	Cdh5,Mmp9	1.336299075
	Relaxin signaling pathway(K)	0.0461	Acta2,Mmp9	1.336299075
	Fluid shear stress and atherosclerosis(K)	0.0461	Cdh5,Mmp9	1.336299075
	Effects of Botulinum toxin(N)	0.0461	Syt1	1.336299075
	Synaptic_vesicle_trafficking(P)	0.0461	Syt1	1.336299075
	Extracellular matrix organization(R)	0.0461	Mmp9,Mmp10	1.336299075
	Syndecan-1-mediated signaling events(N)	0.0461	Mmp9	1.336299075
	Wnt signaling pathway(P)	0.0461	Acta2,Cdh5	1.336299075
	Nicotinic acetylcholine receptor signaling pathway(P)	0.0461	Acta2	1.336299075
	S1P2 pathway(N)	0.0461	Cdh5	1.336299075
	Osteopontin-mediated events(N)	0.0461	Mmp9	1.336299075
	amb2 Integrin signaling(N)	0.0461	Mmp9	1.336299075
	Syndecan-4-mediated signaling events(N)	0.0461	Mmp9	1.336299075
	Validated transcriptional targets of AP1 family members Fra1 and Fra2(N)	0.0461	Mmp9	1.336299075

Module	GeneSet	FDR	Nodes	NegLOGFDR
M2	Beta5 beta6 beta7 and beta8 integrin cell surface interactions(N)	0.0405	Cyr61	1.392544977
	Beta2 integrin cell surface interactions(N)	0.0405	Cyr61	1.392544977
	Primary immunodeficiency(K)	0.0405	Ciita	1.392544977
	Beta3 integrin cell surface interactions(N)	0.0405	Cyr61	1.392544977
	RhoA signaling pathway(N)	0.0405	Cyr61	1.392544977
	Antimicrobial peptides(R)	0.0405	Lcn2	1.392544977
	Iron uptake and transport(R)	0.0405	Lcn2	1.392544977
	AP-1 transcription factor network(N)	0.0405	Cyr61	1.392544977
	Interferon gamma signaling(R)	0.0405	Ciita	1.392544977
	Antigen processing and presentation(K)	0.0405	Ciita	1.392544977
	Regulation of nuclear beta catenin signaling and target gene transcription(N)	0.0405	Cyr61	1.392544977
	IL-17 signaling pathway(K)	0.0405	Lcn2	1.392544977
	Post-translational protein phosphorylation(R)	0.0464	Cyr61	1.333482019
	Interleukin-4 and Interleukin-13 signaling(R)	0.0481	Lcn2	1.317854924
	Toxoplasmosis(K)	0.049	Ciita	1.30980392

Module	GeneSet	FDR	Nodes	NegLOGFDR
M3	Ether lipid metabolism(K)	0.0581	Enpp2	1.235823868
	Interferon gamma signaling(R)	0.0581	Gbp1	1.235823868
	Glycerophospholipid biosynthesis(R)	0.0581	Pla1a	1.235823868
	NOD-like receptor signaling pathway(K)	0.0581	Gbp1	1.235823868
	Ras signaling pathway(K)	0.0796	Pla1a	1.099086932

Module	GeneSet	FDR	Nodes	NegLOGFDR
	Reelin signalling pathway(R)	7.08E-03	Vldlr	2.150236727
	Reelin signaling pathway(N)	7.42E-03	Vldlr	2.12976001
	Lissencephaly gene (LIS1) in neuronal migration and development(N)	7.42E-03	Vldlr	2.12976001
	EPHA forward signaling(N)	7.42E-03	Epha4	2.12976001
	Urokinase-type plasminogen activator (uPA) and uPAR-mediated signaling(N)	7.42E-03	Vldlr	2.12976001
	Plasma lipoprotein assembly, remodeling, and clearance(R)	0.012	Vldlr	1.920818754
	EPH-Ephrin signaling(R)	0.0152	Epha4	1.818156412
M4	Axon guidance(K)	0.0307	Epha4	1.512861625

Module	GeneSet	FDR	Nodes	NegLOGFDR
	Extracellular matrix organization(R)	2.12E-04	Col1a1,Adamts2,Tll1	3.67434069
	VEGFR3 signaling in lymphatic endothelium(N)	0.026	Col1a1	1.585026652
	Beta3 integrin cell surface interactions(N)	0.026	Col1a1	1.585026652
	IL4-mediated signaling events(N)	0.026	Col1a1	1.585026652
	Beta1 integrin cell surface interactions(N)	0.026	Col1a1	1.585026652
	ECM-receptor interaction(K)	0.026	Col1a1	1.585026652
	Protein digestion and absorption(K)	0.026	Col1a1	1.585026652
	Amoebiasis(K)	0.026	Col1a1	1.585026652
	AGE-RAGE signaling pathway in diabetic complications(K)	0.026	Col1a1	1.585026652
	Platelet activation(K)	0.0323	Col1a1	1.490797478
	Binding and Uptake of Ligands by Scavenger Receptors(R)	0.0325	Col1a1	1.488116639
	Relaxin signaling pathway(K)	0.0341	Col1a1	1.467245621
M5	Integrin signalling pathway(P)	0.0413	Col1a1	1.384049948

Module	GeneSet	FDR	Nodes	NegLOGFDR
M6	Neutrophil degranulation(R)	0.0374	S100a11	1.427128398

Supplementary Table 6–List of antibodies used for immuno-histochemistry analysis

Primary antibody name	Company	Catalog #	Host	Dilution
Anti-Nuclei Antibody, Clone 235-1 (HuNu)	Millipore Sigma	MAB1281	Mouse	1:100
Anti-Green Fluorescent Protein (GFP)	Rockland	600-101-215	Goat	1:1000
Anti-Alpha Smooth Muscle Actin (α-SMA/ACTA2)	Abcam	ab5694	Rabbit	1:500
Anti-Glial Fibrillary Acidic Protein (GFAP)	Millipore Sigma	AB5804	Rabbit	1:1000
Anti-Neurofilament-L (C28e10)	Cell Signaling	2837	Rabbit	1:100
Anti-E-Cadherin (24E10)	Cell Signaling	3195	Rabbit	1:400
Anti-N-Cadherin	Abcam	AB18203	Rabbit	1:1000
Anti-Nestin	Novus	NB100-1604	Chicken	1:800
Anti Sox2	Invitrogen	MA1-014	Mouse	1:200
Anti-Iba1 Antibody [EPR16588]	Abcam	ab178846	Rabbit	1:500
Anti-Brdu (Bu20a)	Cell Signaling	5292	Mouse	1:200
Anti-Collagen I	Abcam	ab34710	Rabbit	1:500
Anti-Collagen 1A1	Thermo Fisher Scientific	PA5-29569	Rabbit	1:500
Anti-Fibronectin	Abcam	ab2413	Rabbit	1:1000
Anti-CD68	Abcam	ab125212	Rabbit	1:1000
Anti-CD31	Cell Signaling	77699	Rabbit	1:100
Anti-Survivin	Cell Signaling	2808	Rabbit	1:500
Anti-PCNA	Cell Signaling	2586	Mouse	1:1000
Anti-Cleaved Caspase 3	Cell Signaling	9661	Rabbit	1:400
Anti-TdTomato	SICGEN	AB8181-200	Goat	1:200
Anti-Glial Fibrillary Acidic Protein (GFAP)	EMD Millipore	AB5541	Chicken	1:500
Anti-Green Fluorescent Protein (GFP)	Abcam	AB290	Rabbit	1:500
**Secondary antibody name**	**Company**	**Catalog #**	**Host**	**Dilution**
Polyclonal Goat Anti-Rabbit Immunoglobulins/Biotin	Agilent	E043201-6	Goat	1:1000
Polyclonal Goat Anti-Mouse Immunoglobulins/Biotinylated	Agilent	E0433	Goat	1:1000
Goat Anti-Chicken IgY Antibody (H+L), Biotinylated	Vector Laboratories	BA-9010-1.5	Goat	1:1000
Alexa Fluor 555 Goat anti-Chicken IgY (H+L)	Invitrogen	A21437	Donkey	1:1000

Alexa Fluor Plus 488 Donkey anti-Rabbit IgG (H+L)	Invitrogen	A32790	Donkey	1:1000
Alexa Fluor Plus 488 Donkey anti-Goat IgG (H+L)	Invitrogen	A32814	Donkey	1:1000
Alexa Fluor Plus 555 Donkey anti-Rabbit IgG (H+L)	Invitrogen	A32794	Donkey	1:1000
Alexa Fluor Plus 647 Donkey anti-Goat IgG (H+L)	Invitrogen	A21447	Donkey	1:1000
Alexa Fluor 594 Donkey anti-Chicken IgY (IgG) (H+L)	Jackson Immuno-Research Labs	703-585-155	Donkey	1:1000
Alexa Fluor 488 Donkey Anti-Mouse IgG (H+L)	Invitrogen	A21202	Donkey	1:1000

Supplementary Table 7- IOU values over the testing set.

Dataset	Number of Slides	IOU (%, mean)	IOU (%, sd)	Range (%)
Mouse	30	76.6	14.2	41.1 - 94.4
TCGA II	11	100*	0	100
TCGA III	11	73.6	11.7	54.9 - 87.9
TCGA IV	12	75.5	12.7	52.6 - 97.3
All	64	80.3	11.2	52.9 - 100

* No oncostreams were present in TCGA II data and our model did not have any false positive errors for this dataset

Other Supplementary information

Supplementary Data 1. Oncostreams analysis using TCGA-GBM and TCGA-LGG histological tissues database

Supplementary Data 2. Oncostreams' differentially gene expression analysis

Movies 1 to 4. Glioma dynamics at the tumor core.
Time-lapse confocal imaging of explant brain slice cultures of NPA glioma cores. Movement of tumor cells GFP positive (green) were analyzed within the tumor core. Imaging was obtained every 10 minutes for the duration of 293 cycles.

Movies 5 to 9. Glioma dynamics at the tumor border.
Glioma dynamic at the tumor border were analyzed using explant slice cultures glioma model by intracranial implantation of GFP positive NPA cells into the striatum of B6.129(Cg)-Gt(ROSA)26Sortm4(ACTB-**tdTomato**-EGFP)Luo/J- transgenic mice. Normal brain parenchyma is visualized in red. Imaging was obtained every 10 minutes for the duration of 186 cycles.

Movies 10 to 13. Glioma dynamics of NPAshCOL1A1 tumors
Cell glioma dynamic of NPA-ShCOL1A1 tumors were analyzed using explant slice cultures glioma model by intracranial implantation of GFP positive NPAshCOL1A1 cells into the striatum of B6.129(Cg)-Gt(ROSA)26Sortm4(ACTB-**tdTomato**-EGFP)Luo/J- transgenic mice. Normal brain parenchyma is visualized in red. Imaging was obtained every 10 minutes for the duration of 220 cycles.

Movies 14-16. *In vivo* NPA glioma dynamics patterns employing 2-Photon intravital imaging
GFP positive NPA glioma cells were intracranially implanted into the cortex of the brain of B6.129(Cg)-Gt(ROSA)26Sortm4(ACTB-tdTomato-EGFP)Luo/J- transgenic mice. Glioma dynamics patterns were evaluated by time-lapse intravital imaging using a two-photon microscope. Normal brain parenchyma is visualized in red and the glioma cells in green. Images were obtained every 5 minutes.

Movie 17. *In vivo* NPAshCOL1A1 glioma dynamics patterns employing 2-Photon intravital imaging
GFP positive NPAshCOL1A1 glioma cells were intracranially implanted into the cortex of the brain of B6.129(Cg)-Gt(ROSA)26Sortm4(ACTB-tdTomato-EGFP)Luo/J- transgenic mice. Glioma dynamics patterns were evaluated by time-lapse intravital imaging using a two-photon microscope. Normal brain parenchyma is visualized in red and the glioma cells in green. Images were obtained every 5 minutes.

Fig. S1

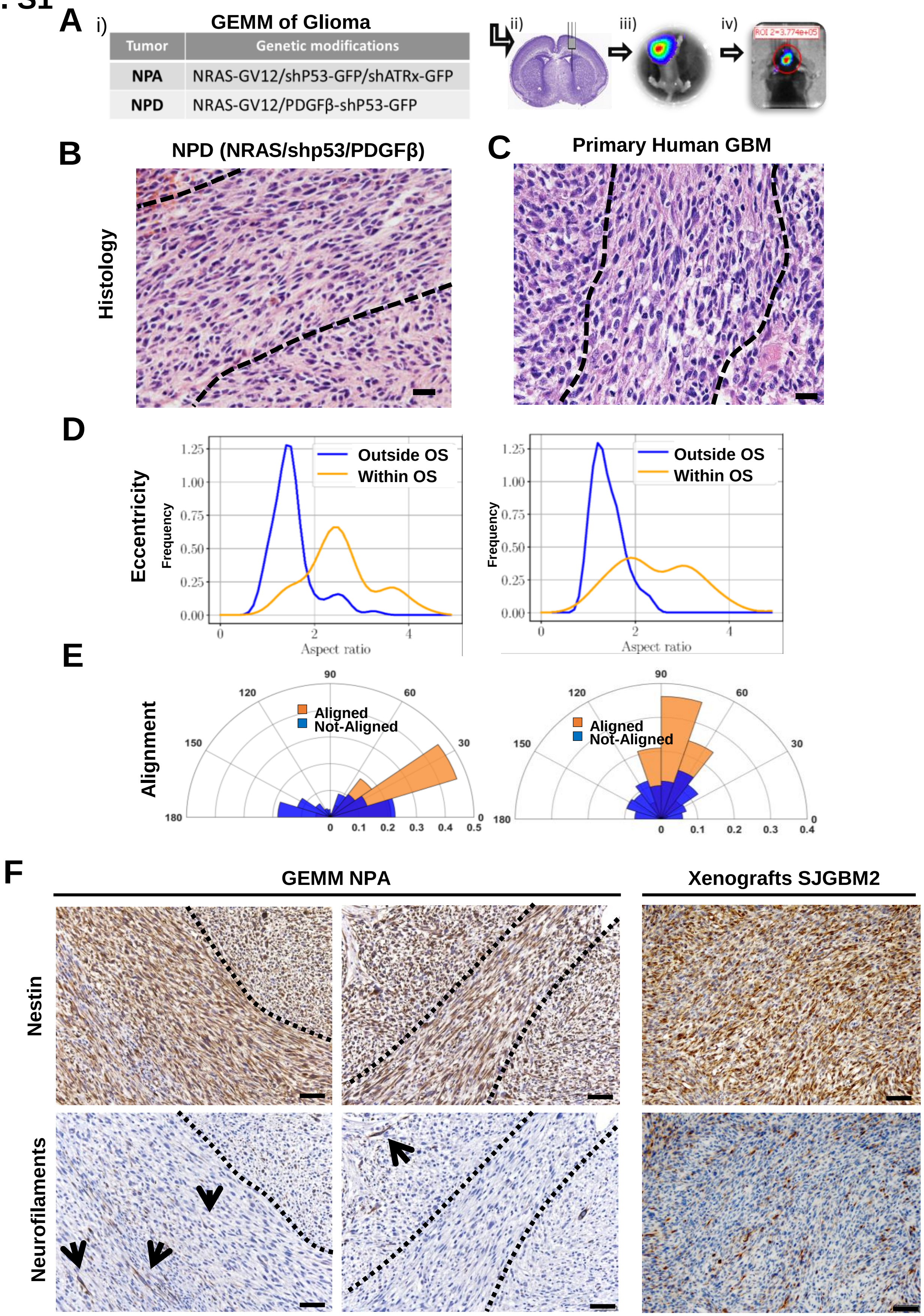

A
i)
GEMM of Glioma
Tumor
Genetic modifications
NPA
NRAS-GV12/shP53-GFP/shATRx-GFP
NPD
NRAS-GV12/PDGFβ-shP53-GFP
ii)
iii)
iv)
B
NPD (NRAS/shp53/PDGFβ)
Histology
C
Primary Human GBM
D
Eccentricity
Frequency
Outside OS
Within OS
Aspect ratio
E
Alignment
Aligned
Not-Aligned
F
GEMM NPA
Xenografts SJGBM2
Nestin
Neurofilaments

Fig. S1: Oncostream characterization in mouse and human glioma tissue. A) Schematic representation of the genetically engineered mouse models (GEMM) of glioma generated using the Sleeping Beauty transposase system; i) genetic makeup of NPA and NPD gliomas, ii) plasmid injection into the lateral ventricle of neonatal mice, iii) bioluminescence imaging of a neonatal mouse 1 day post-injection, iv) bioluminescence detection at 70 days post-injection. **B-C)** Representative Hematoxylin and Eosin 5 µm of 5 independent tumor sections from gliomas showing that elongated glioma cells forming oncostreams (dotted line) are present in NPD (NRAS/shp53/PDGFβ) GEMM, and in human glioma tissue. Scale bars: 50 µm. **D**) Cellular eccentricity and alignment analysis on glioma GEMM (NPD) and human glioma representative images. Histograms of cellular aspect ratio **(D)** and cellular alignment **(E)** shows cells within oncostreams are elongated and aligned, whereas outside of oncostreams, they are rounded and not-aligned. **F)** Immunohistochemistry staining for Neurofilaments-L and Nestin in three independent tumors GEMM-NPA model and xenograft models (SJGBM2 human cells), showing that oncostreams are not specially organized alongside brain axonal pathways. Scale bars: 50 µm. Arrowheads indicate the presence of Neurofilaments immunoreactivity.

Fig. S2

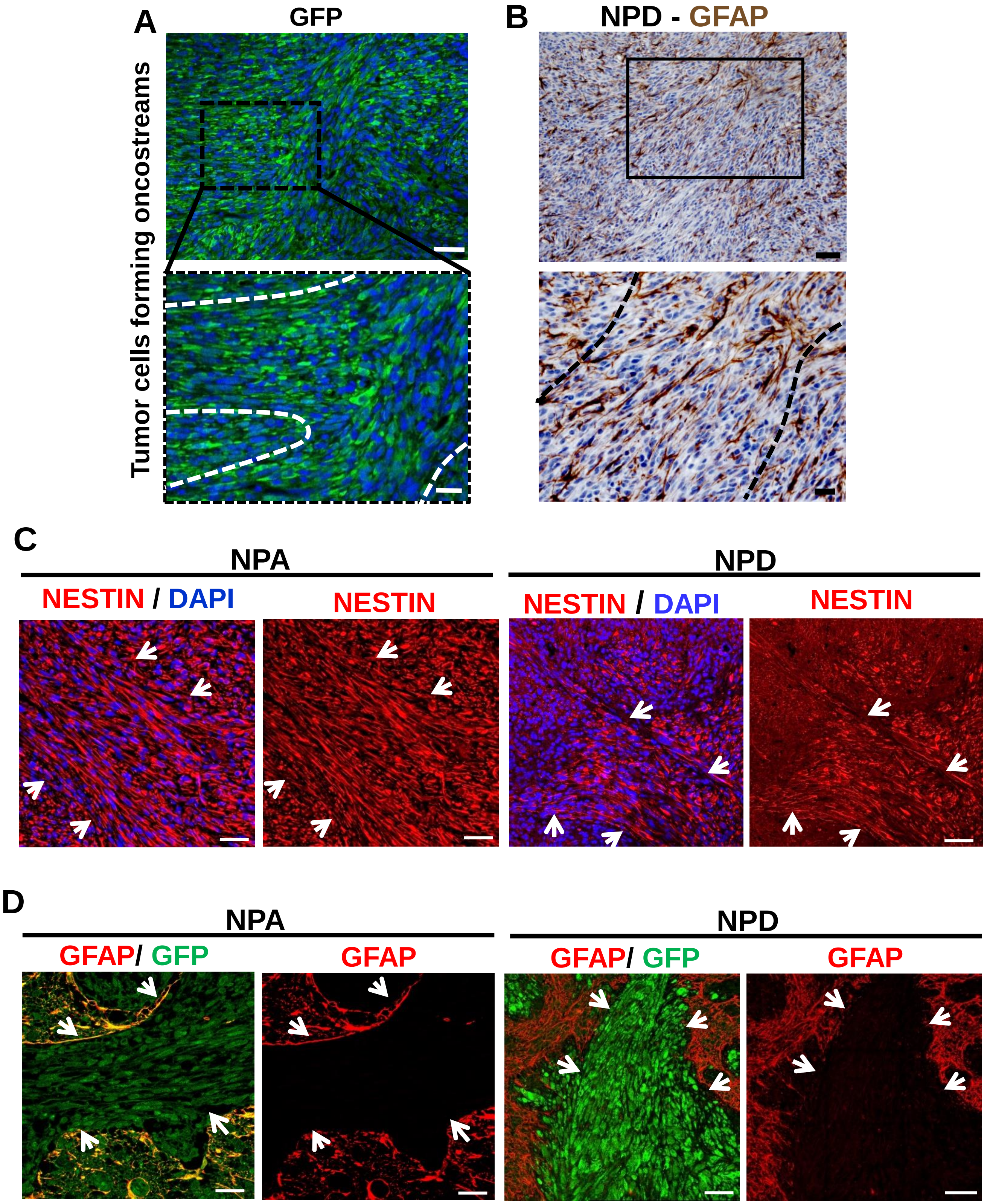

A
GFP
Tumor cells forming oncostreams
B
NPD - GFAP
C
NPA
NESTIN / DAPI
NESTIN
NPD
NESTIN / DAPI
NESTIN
D
NPA
GFAP/ GFP
GFAP
NPD
GFAP/ GFP
GFAP

Fig. S2: Oncostreams are formed by aligned tumor cells and include cells from the tumor microenvironment.

Oncostreams' cellular heterogeneity was analyzed by immunofluorescence staining in three independent tumor samples of each model. **A)** GFP expression in NPA tumors show oncostreams formed by tumor cells (green = tumor cells; and blue = DAPI stained nuclei). Scale bar: 100 µm (top) and 50 µm (bottom). **B)** Immunohistochemistry analysis on GEMM of glioma (NPD) shows alignment of GFAP+ cells within oncostreams. Scale bars: 50 µm (top) and 20 µm (bottom). **C)** Representative confocal images of GEMM NPA and NPD glioma illustrates the presence of Nestin (red) within oncostreams. Nestin is a highly expressed marker in glioblastomas and expression correlate with tumor prognosis. Nuclei were stained with DAPI (left). Scale bar: 50 µm. **D)** Confocal images of GEMM NPA and NPD glioma show the expression of GFP+ (green) in tumor cells within streams, and tumor microenvironment GFAP+ cells (red) surrounding the oncostream (right panel). GFAP, is expressed by reactive astrocytes or neoplastic astrocytes. Oncostreams either display GFAP+ cells throughout the structures (Fig. S2B) or surrounding the oncostreams (Fig. S2D). GFAP+ cells within oncostreams most likely are tumor cells, while GFAP+ cells surrounding oncostreams are reactive astrocytes. Scale bar: 50 µm. Arrowheads indicate oncostreams areas surrounded by GFAP cells.

Fig. S3

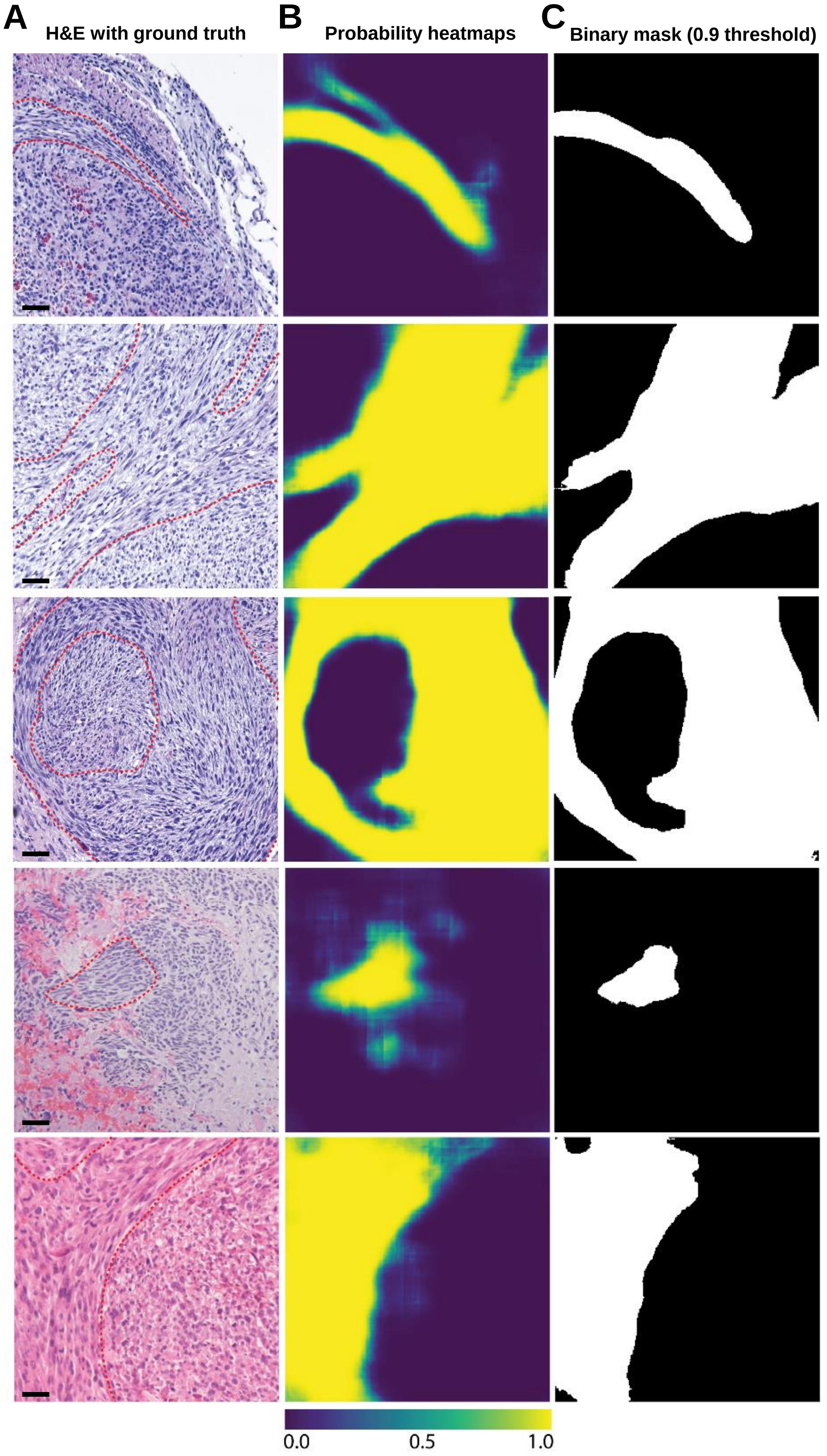
A
H&E with ground truth
B
Probability heatmaps
C
Binary mask (0.9 threshold)
0.0
0.5
1.0

Fig. S3: A deep learning model identifies oncostreams in mouse glioma H&E histological images.

A-C) We implemented a U-Net architecture to provide semantic segmentation of oncostream fascicles in glioma histological sections. Independent tumor images (n=105) were used, and the experiment was repeated 6 times as shown in Fig. S4. **A)** Mouse Hematoxylin and Eosin (H&E) images are shown with oncostreams manually segmented (stippled/dashed red lines). Scale bar: 50 µm. **B)** Model output is a semantic segmentation probability heatmap with each pixel being assigned a probability of being within an oncostream (foreground, yellow), or not (background, deep purple). Probability heatmaps for each corresponding H&E images are shown. **C)** Probability heatmaps can be converted to binary masks (foreground versus background) using probability thresholding. Oncostream binary masks with probability threshold of >0.9 are shown. Threshold was tuned during validation as a hyperparameter.

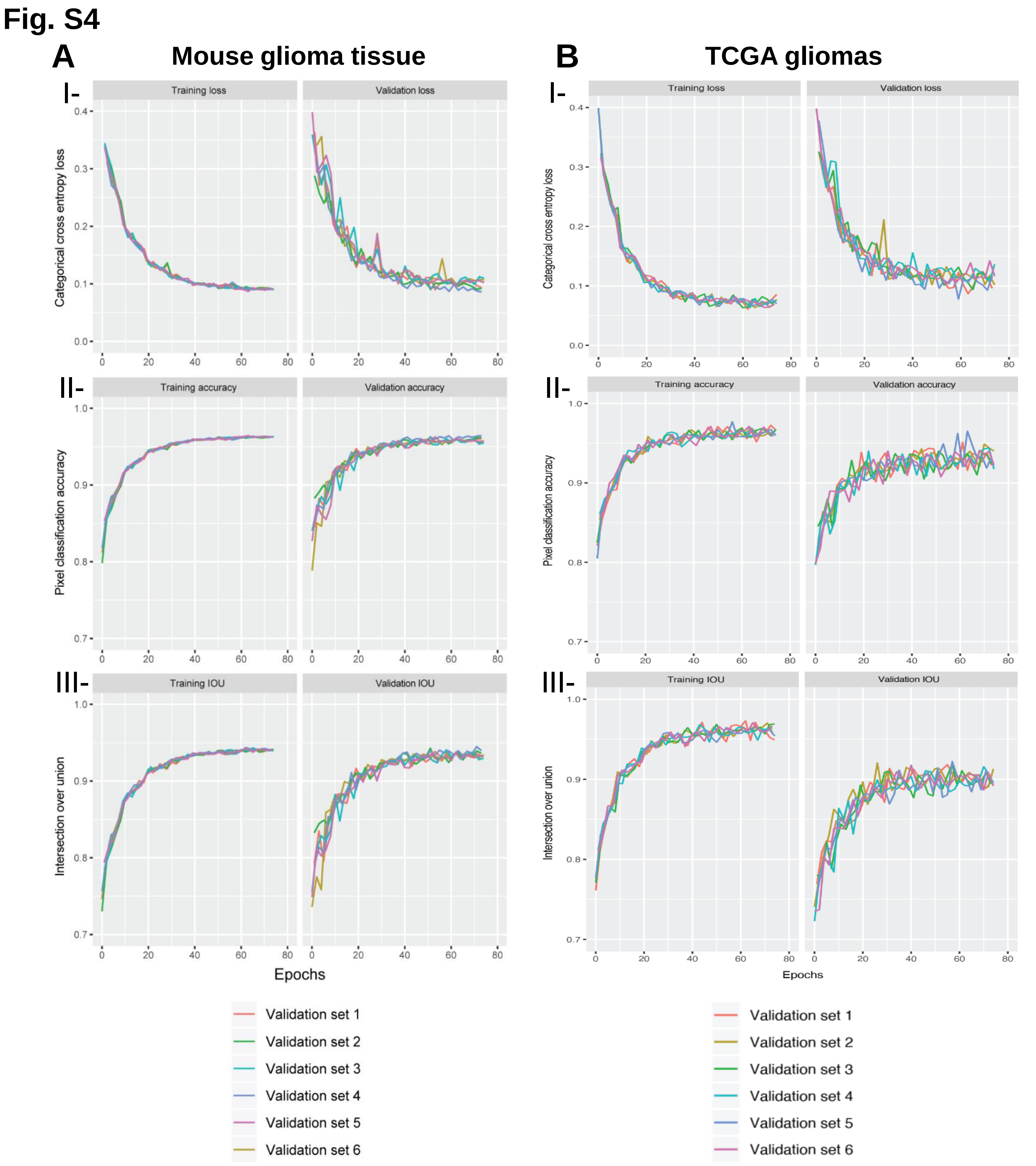

Fig. S4. Training and validation curves. A-B) Training and validation curves for a fully convolutional neural network trained using the mouse dataset (**A**) and TCGA dataset (**B**) are shown for the **(I)** cross entropy loss, **(II)** pixel classification accuracy, and **(III)** intersection over union (IOU) metric. We performed 6 iterations of random training-validation dataset splits (80%/20%) and held out the TCGA data for validation. Training and validation metrics stabilized after 75 epochs.

Fig. S5

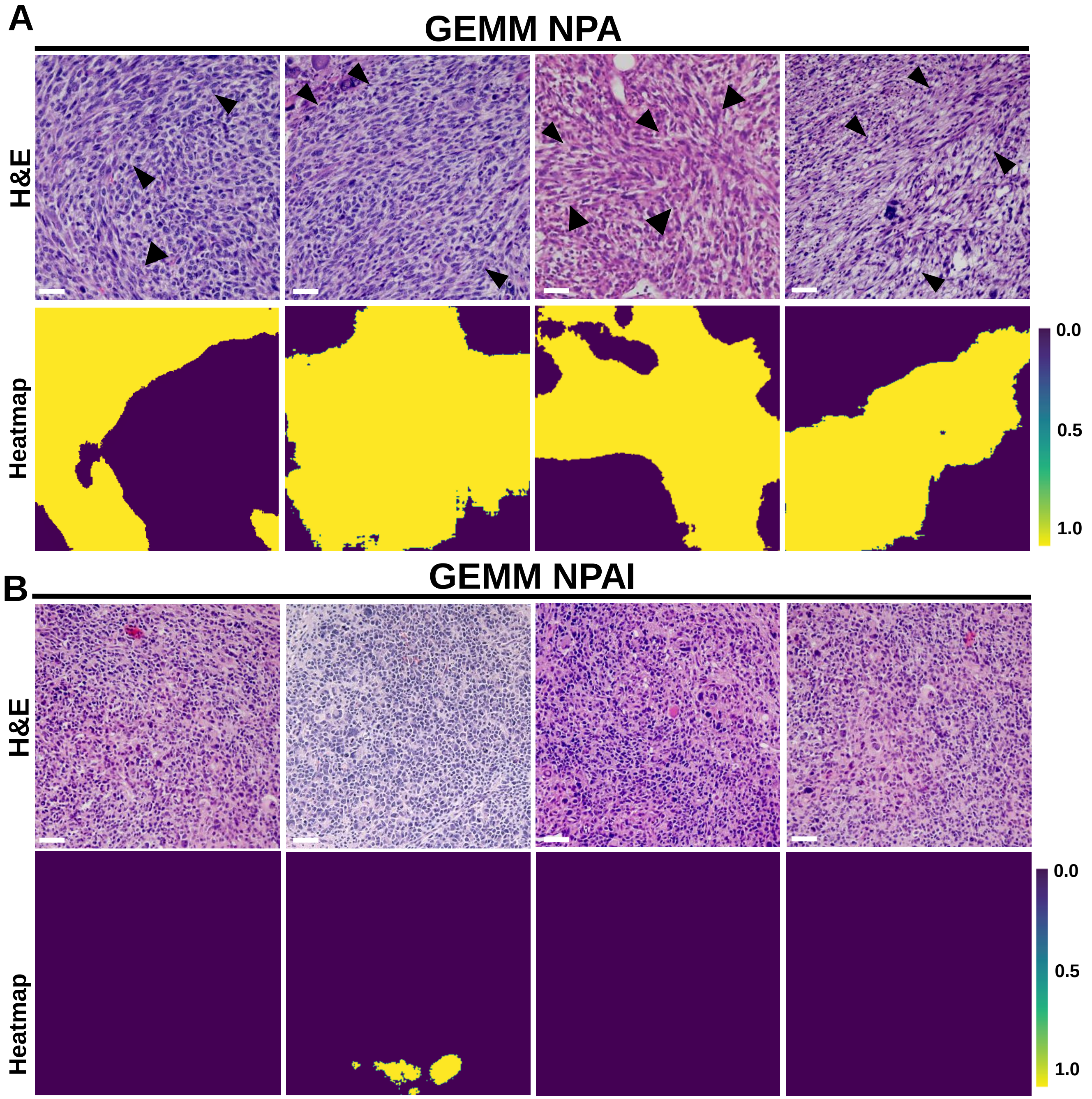

Fig. S5: Schematic segmentation of oncostreams areas on IDH-WT (NPA) and IDH-Mutant (NPAI) mouse genetically engineered glioma model. A-B) Representative images of oncostreams manually segmented on Hematoxylin and Eosin (H&E) stained sections of NPA (IDH-WT) gliomas (**A**) and NPAI (IDH-Mut) gliomas (**B**); (oncostreams are indicated with black arrowheads). Scale bar: 50 µm. 'Heatmap' row illustrates the semantic segmentation probability heatmaps for each corresponding H&E image. Quantification of A and B is shown in Fig. 2D.

Fig. S6

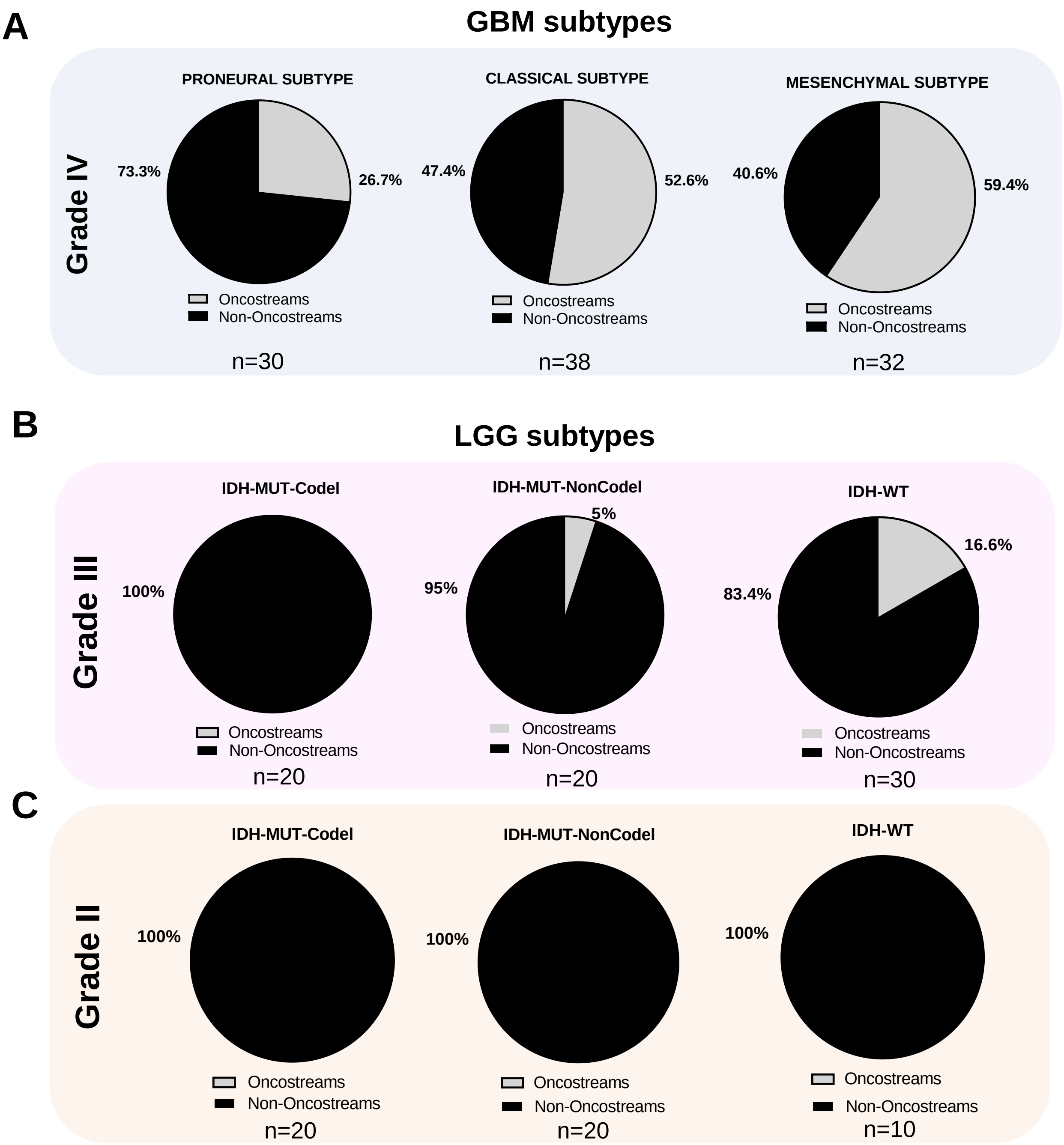

Fig. S6. A) Pie charts show percentage of tumors displaying oncostreams in GBM tumors (Grade IV) classified by molecular subtypes. Oncostreams are displayed in Proneural subtype (26.7%), Classical subtype (52.6%) and Mesenchymal subtype (59.4%) . **B)** Pie charts show percentage of tumors showing oncostreams in LGG tumors (Grade III) classified by molecular subtypes (IDH1-status and 1p-19q codeletion). Oncostreams are present in 5% of IDH1-Mutant-1p-19q-No-codeletion and in 16.6% of IDH1-WT tumors. **C)** Pie charts show percentage of tumors with oncostreams in LGG tumors (Grade II) classified by molecular subtypes (IDH1-status and 1p-19q codeletion). No oncostreams are observed in Grade II gliomas.

Fig. S7

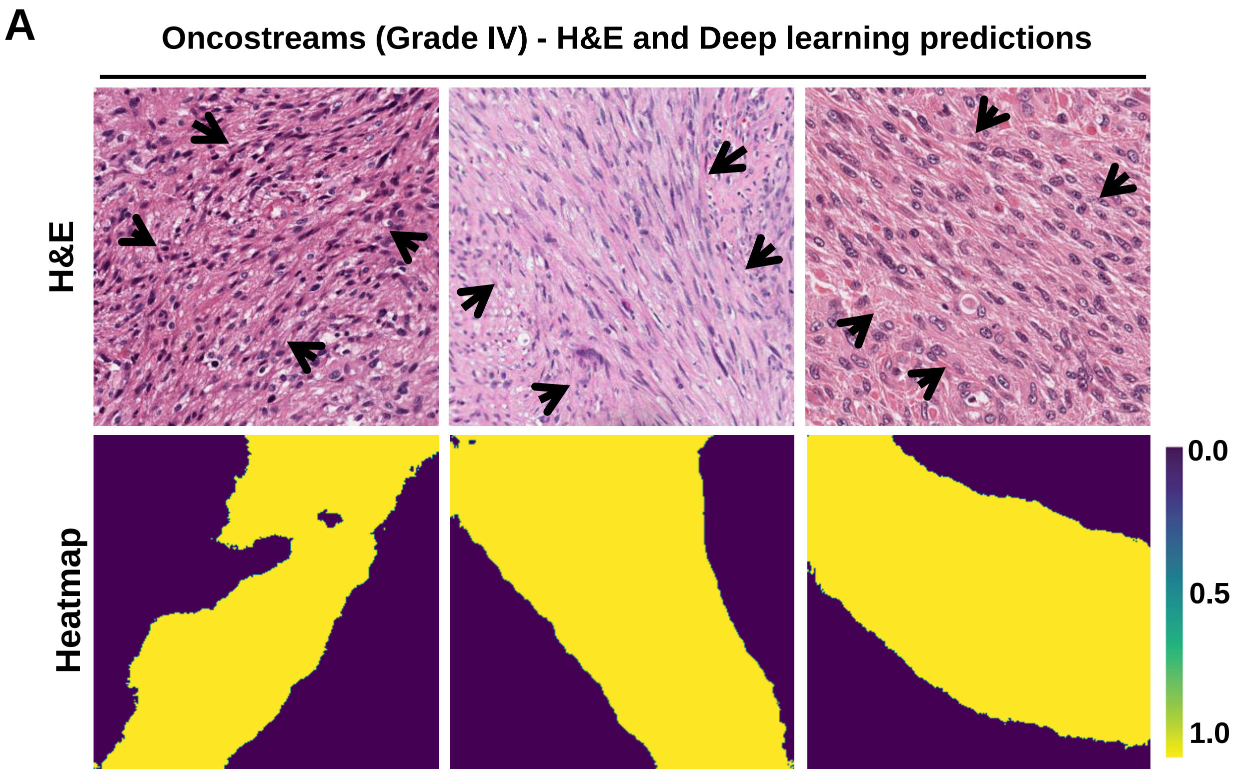

Fig. S7: Oncostreams are identified by deep learning in human high-grade gliomas (Glioblastoma - Grade IV). A) Representative Hematoxylin and Eosin (H&E) images of TCGA-glioblastoma multiforme (WHO Grade IV) diagnostic slides show the presence of manually segmented oncostreams (indicated by black arrows). Experiment was performed in 109 tumor sections as indicated in Fig. 3B-C. The second row shows semantic segmentation probability heatmaps for each corresponding H&E image.

Fig. S8

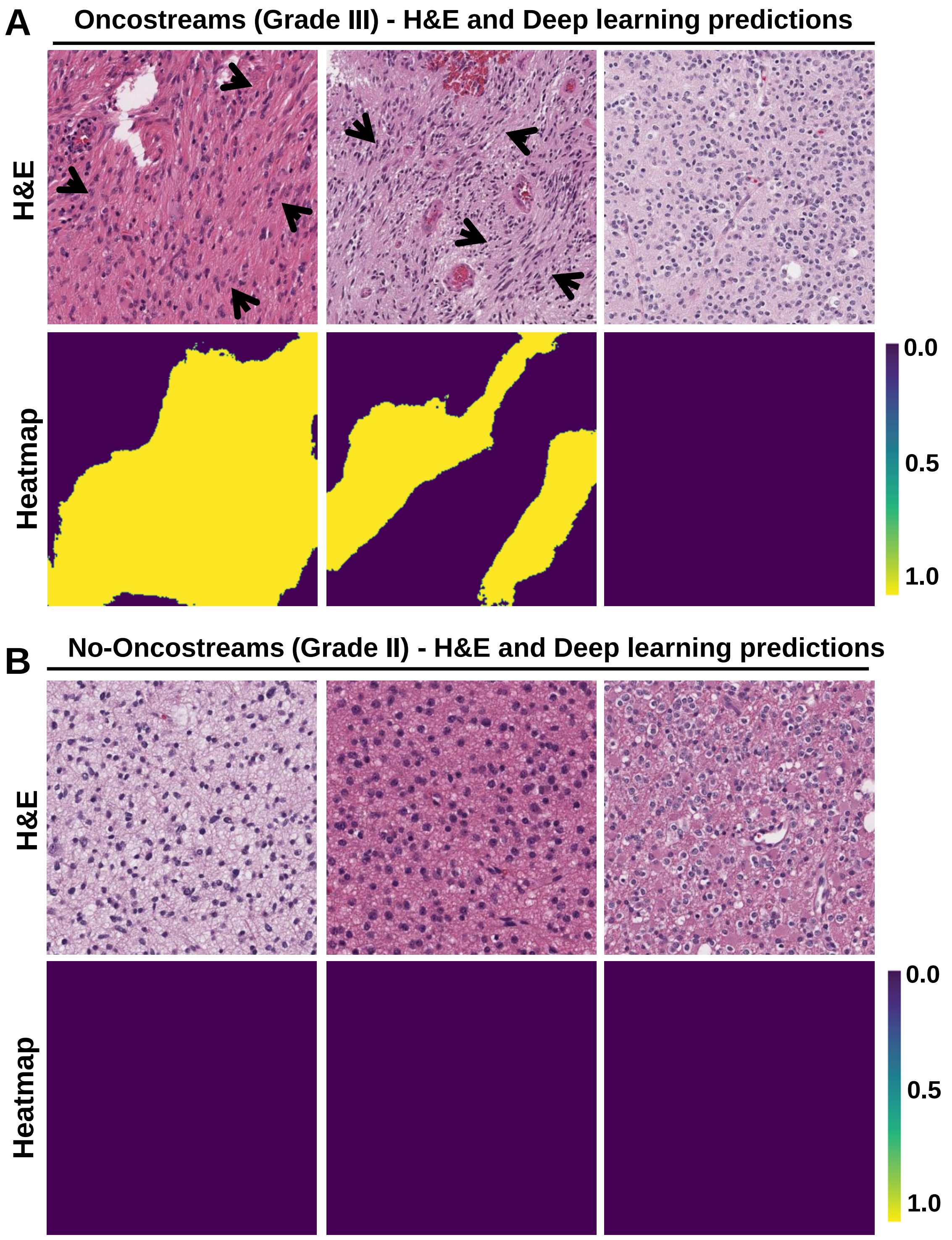

Fig. S8: Oncostreams are observed in a low percentage of human gliomas WHO grade III but not in WHO grade II. A-B) Representative Hematoxylin and Eosin (H&E) images of TCGA gliomas WHO Grade III **(A)** and Grade II **(B)** samples show the presence of manually segmented oncostreams (indicated by black arrows). Semantic segmentation probability heatmaps for each corresponding H&E image are shown in the row labeled 'heatmap'. Experiment was performed in 126 (grade II) and 61 (Grade II) tumor sections as indicated in Fig. 3B-C.

Fig. S9

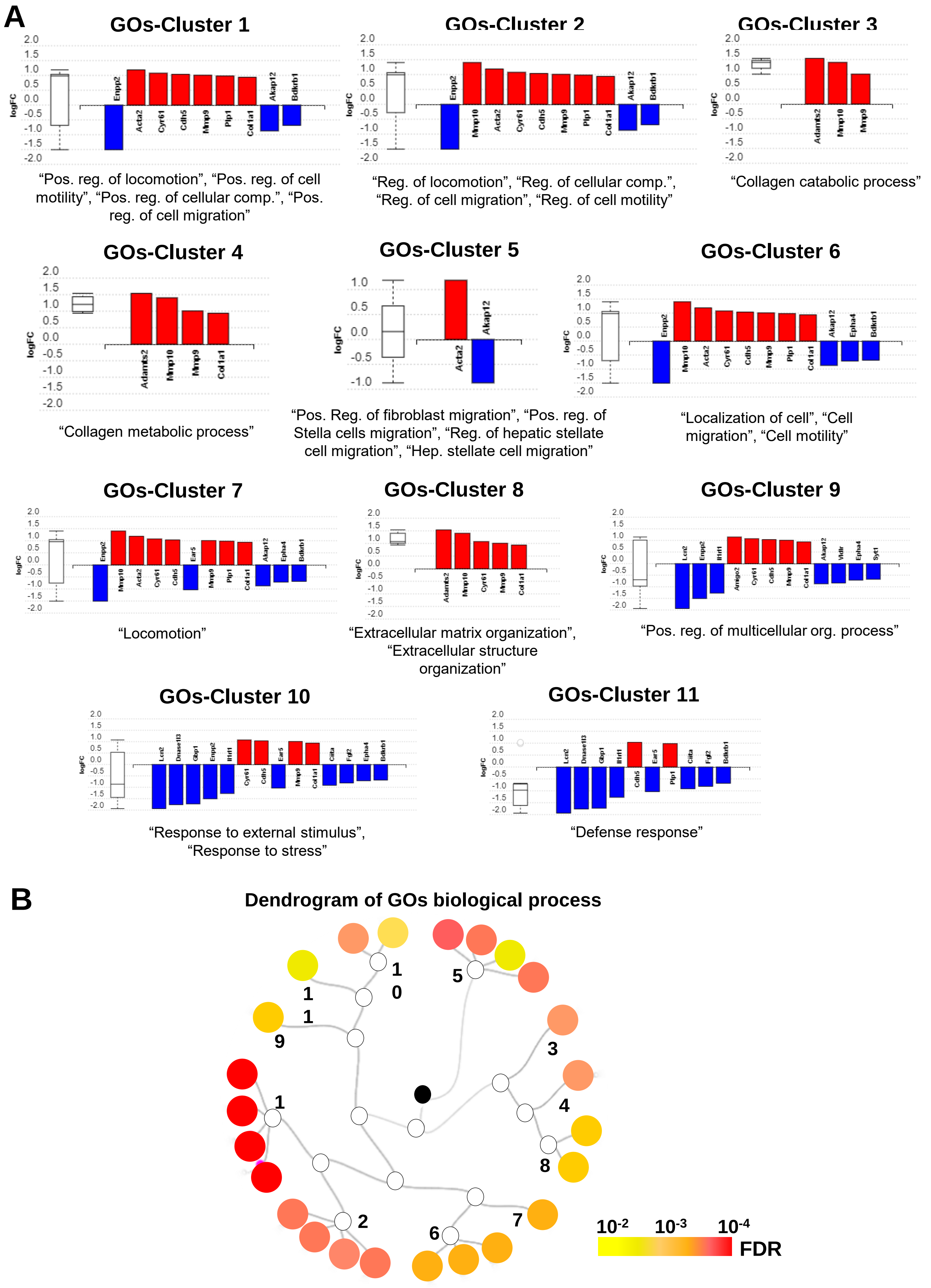
A
GOs-Cluster 1
“Pos. reg. of locomotion”, “Pos. reg. of cell motility”, “Pos. reg. of cellular comp.”, “Pos. reg. of cell migration”
GOs-Cluster 2
“Reg. of locomotion”, “Reg. of cellular comp.”, “Reg. of cell migration”, “Reg. of cell motility”
GOs-Cluster 3
“Collagen catabolic process”
GOs-Cluster 4
“Collagen metabolic process”
GOs-Cluster 5
“Pos. Reg. of fibroblast migration”, “Pos. reg. of Stella cells migration”, “Reg. of hepatic stellate cell migration”, “Hep. stellate cell migration”
GOs-Cluster 6
“Localization of cell”, “Cell migration”, “Cell motility”
GOs-Cluster 7
“Locomotion”
GOs-Cluster 8
“Extracellular matrix organization”, “Extracellular structure organization”
GOs-Cluster 9
“Pos. reg. of multicellular org. process”
GOs-Cluster 10
“Response to external stimulus”, “Response to stress”
GOs-Cluster 11
“Defense response”
logFC
B
Dendrogram of GOs biological process
FDR
10^{-2} 10^{-3} 10^{-4}

Fig. S9. Enrichment in migration and extracellular matrix biological process suggest oncostreams are mesenchymal migratory structures.

A) Bar graphs show clusters of enriched Gene Ontologies (GO) biological process sharing same differentially expressed genes. Pos: Positive, Reg: Regulation. Upregulated genes are shown in red, downregulated genes are shown in blue. Differentially Expressed (DE) genes have been determined using n=3 biologically independent replicates per group. The box and whisker plot on the left summarizes the distribution of all the differentially expressed genes in this pathway. Centre line of the boxplot is the median, upper bound of the boxplot is the Q3 (75th percentile), the lower bound of the boxplot is the Q1(25th percentile), the upper whiskers is the maximum value of the data (within 1.5 times the interquartile range over the 75th percentile), and the lower whisker is the minimum value of the data (within 1.5 times the interquartile range under the 25th percentile). Outliers are represented by circles. The data were analyzed using Advaita Bio's iPathwayGuide. **B)** Dendrogram of GOs biological process. Significant results are organized hierarchically based on overlap in associated DE genes. Node color (yellow to red: 10^{-2} to 10^{-4}) represent significant differences (FDR corrected p-value), black dot represent the center of the dendrogram.

Fig. S10

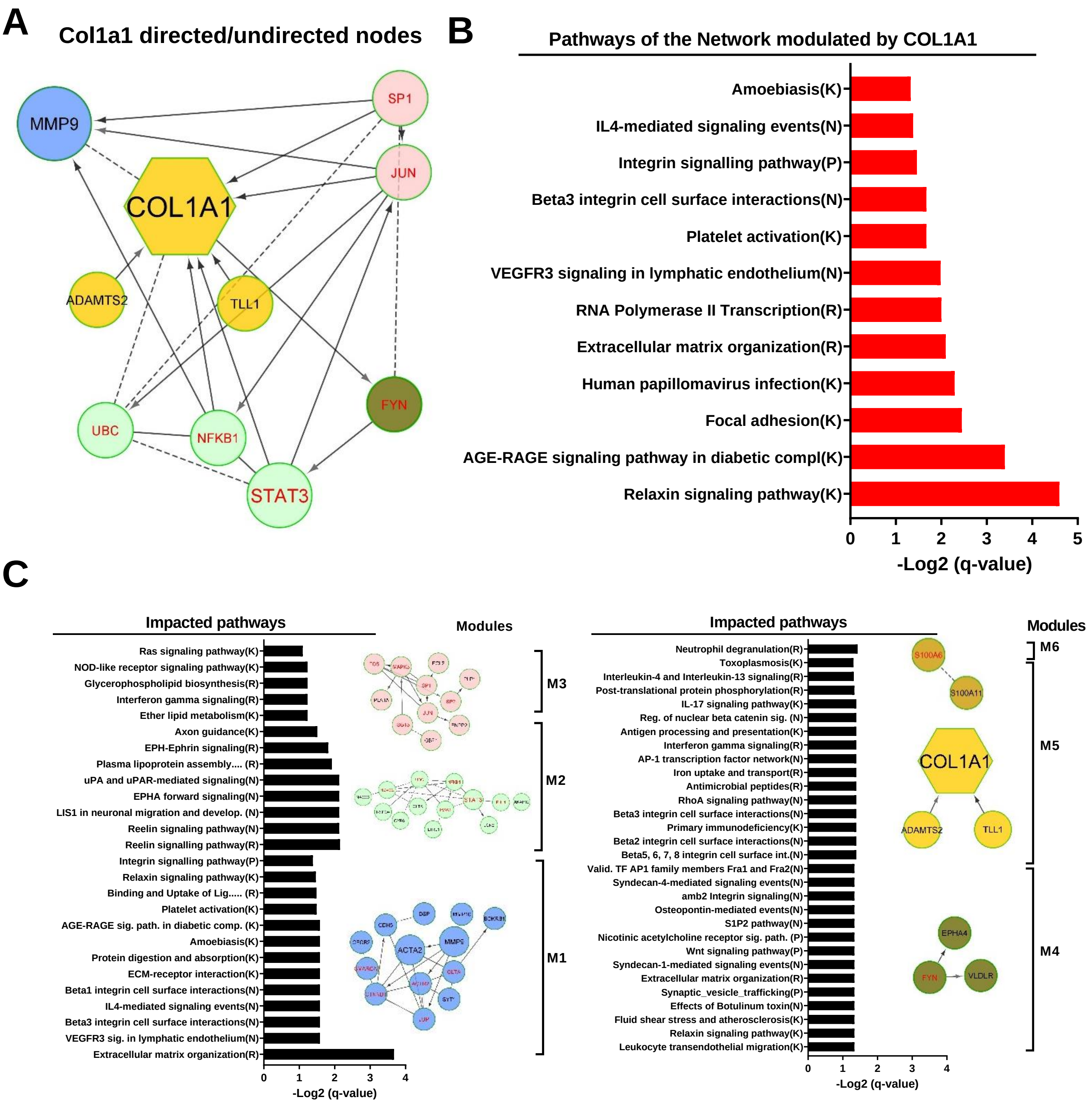

Fig. S10. Pathway enrichment analyses of the COL1A1 network derived from DE genes of oncostreams vs no-oncostreams. A) COL1A1 network showing its first neighbors. COL1A1 is a hub node with a degree of 9 interactions in the network (2nd most connected Differentially Expressed node). **B)** Functional enrichment analysis of the gene ontology (GO) terms of the COL1A1 network. The bar graph displays over-represented GOs biological process that include COL1A1. GO term significance was determined by a cutoff of q-value (FDR) < 0.05. GO terms were plotted against the minus Log 2 of the q-value (FDR). **C)** Functional enrichment analysis of the impacted pathways in each module of the network. Modules (nodes of the same color) represent a highly interacting group of genes in the network. The bar graph shows the overrepresented pathways plotted according to the minus Log2 q-value (FDR). Pathway-selection cutoff was set at a q-value (FDR) <0.05.

Fig. S11

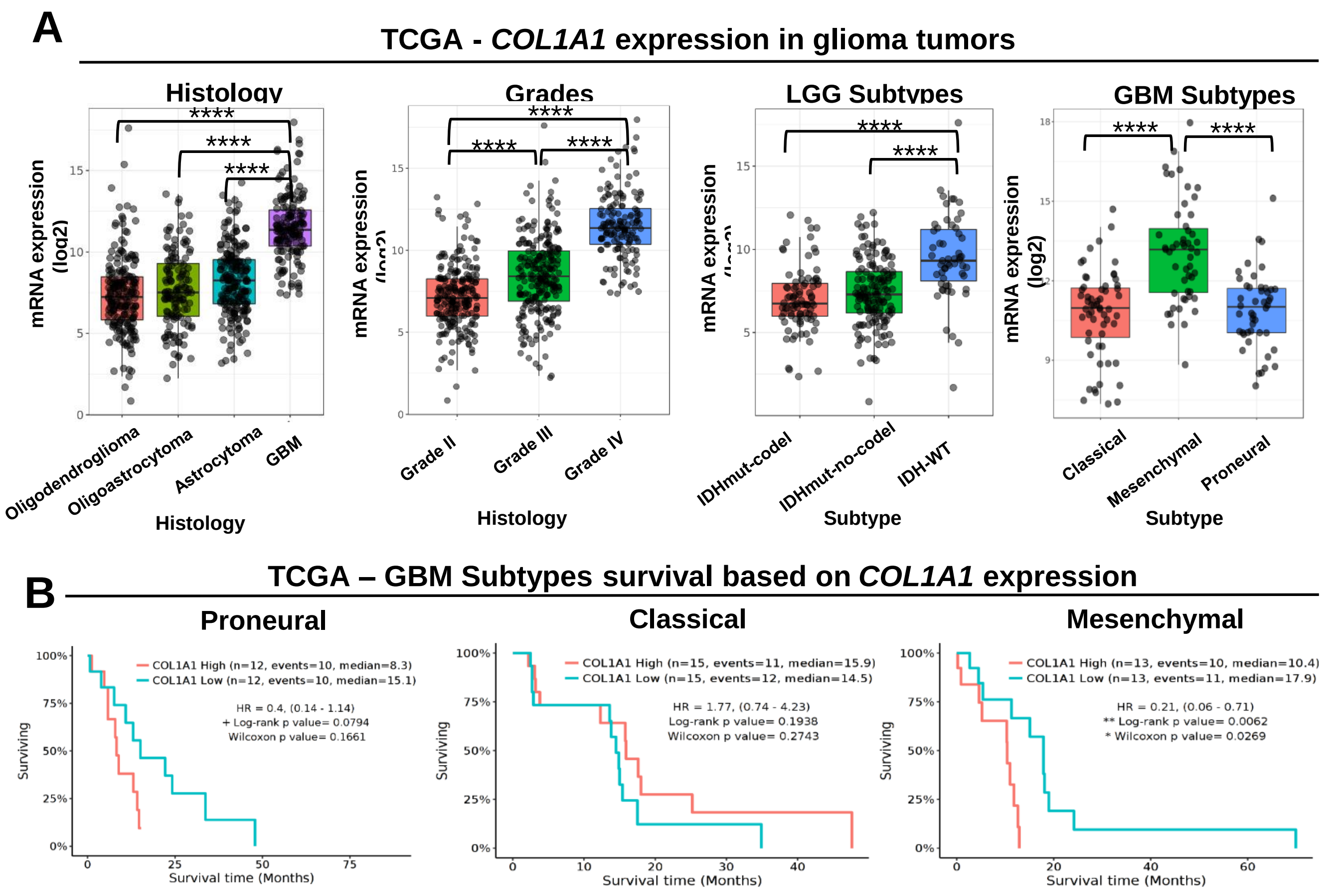

Fig. S11. Evaluation of *COL1A1* expression levels associated with human glioma aggressiveness and clinical outcomes. A) mRNA expression analysis of COL1A1 related to glioma tumor histology, grade and LGG-subtypes and GBM-subtypes (TCGA-GBMLGG database). Graph shows the $\log_2$ mRNA expression levels. Centre line of the boxplot is the median, upper bound of the boxplot is the Q3 (75th percentile), the lower bound of the boxplot is the Q1(25th percentile), the upper whiskers is the maximum value of the data (within 1.5 times the interquartile range over the 75th percentile), and the lower whisker is the minimum value of the data (within 1.5 times the interquartile range under the 25th percentile). Statistical significance of the data set was calculated using One-way ANOVA test and multiple comparison Tukey test, ****p<0.0001. Histology (GBM: n=152, Astrocytoma: n=194, Oligodendroglioma: n=191, Oligoastrocytoma: n=130). Grades (Grade II: n=226, Grade III: n=244, Grade IV: n=152). GBM subtypes (Proneural: n=46; Mesenchymal: n=51; Classical: n=59). LGG Subtypes (IDHwt: n=54; IDHmut-codel: n=85, IDHmut-no-codel: n=141). **B)** Kaplan-Meier survival curves of GBM patients comparing COL1A1 high (red) vs low (blue) expression in GBM molecular subtypes (TCGA-GBMLGG database). Data was analyzed from the Gliovis website (http://gliovis.bioinfo.cnio.es). GBM tumors with high COL1A1 expression show a significant increased survival compared to GBM COL1A1 low within the mesenchymal subtype.

Fig. S12

A

shRNA	Sequence
**Scramble**	GAATCTAATCGTATCGTGGCTG
**shCol1a1-A**	CCCTGGTGATACTGGTGTTAAA
**shCol1a1-B**	GCAACAGTCGCTTCACCTACA
**shCol1a1-C**	GCAAGACAGTCATCGAATACA

B

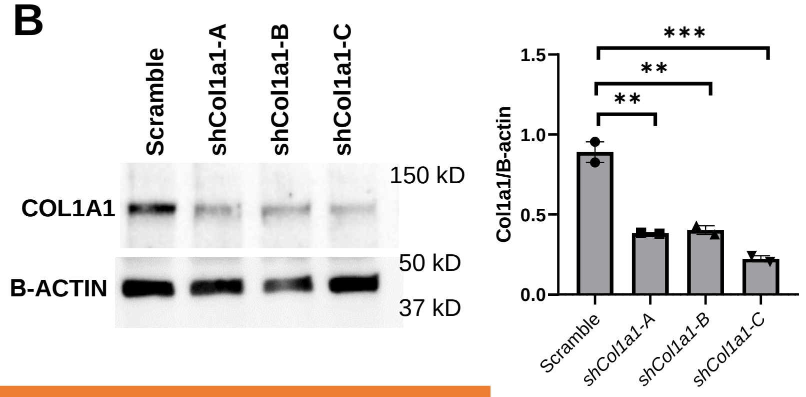

C

Tumor Model	Genetic Modifications
**NPA**	*Nras-GV12/shP53/shATRx*
**NPAshCol1a1**	*Nras-GV12/shP53/shATRx/shCol1a1*
**NPD**	*Nras-GV12/shP53/PDGFβ*
**NPDshCol1a1**	*Nras-GV12/shP53/PDGFβ/shCol1a1*

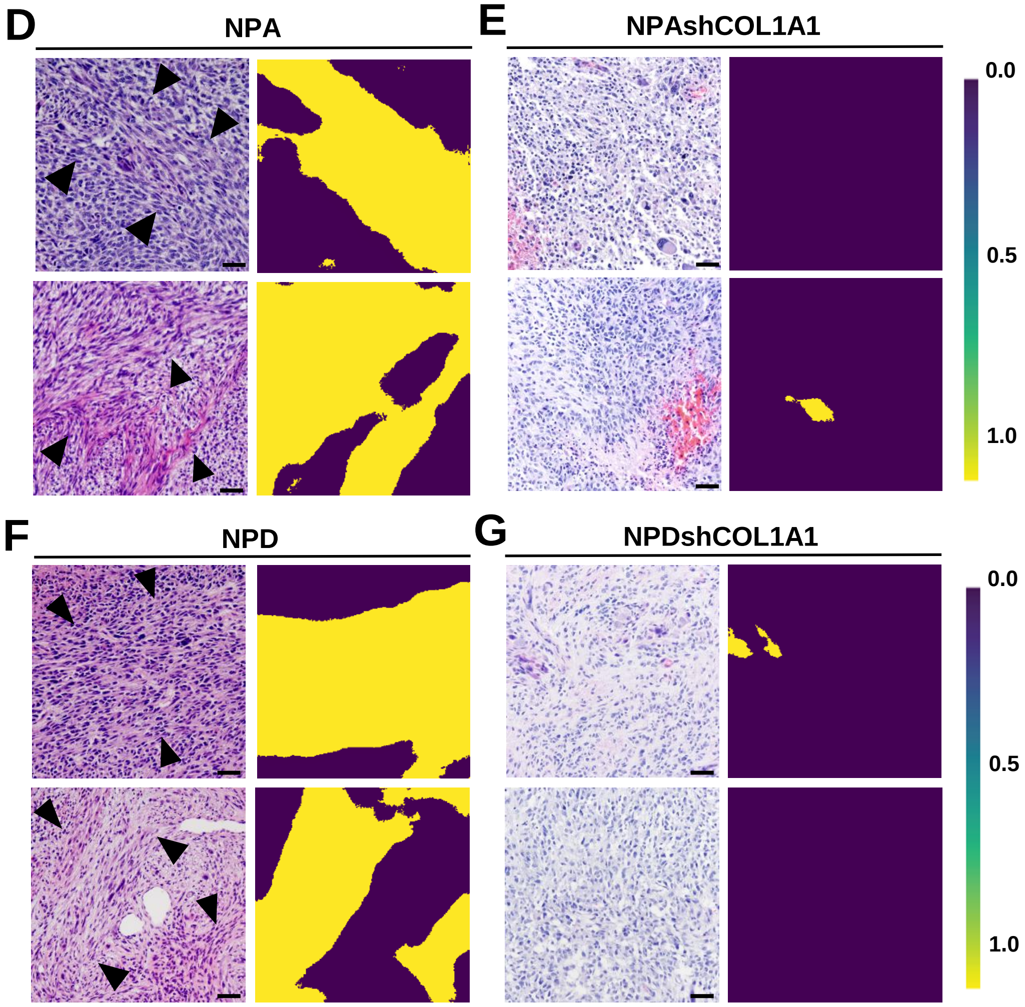

Fig. S12. Genetically engineered mouse glioma models for COL1A1 knockdown.
A) Scramble and shRNAs sequences. These oligonucleotides were cloned in PT2 vectors. **B)** Western Blot (WB) analysis shows downregulation of COL1A1 expression levels in NIH-3T3 mouse cells transfected for 72 hours with the corresponding vectors (Scramble control and 3 different shRNAs). Bar graphs represent the quantitative analysis of the WB. Values were calculated by normalizing COL1A1 expression to β-actin. Quantification was performed using ImageJ software. Statistical significance was determined using One-way ANOVA test. Graphs present mean ±SEM. n=2 biologically independent samples. Scramble vs. shCol1a1-A **p=0.0013; Scramble vs. shCol1a1-B **p=0.0015; Scramble vs. shCol1a1-C ***p=0.0005. Experiment was repeated two times. **(C)** Genetic modifications used to induce different mouse glioma models. **D-G)** Representative images of oncostreams manually segmented on Hematoxylin and Eosin (H&E) stained sections of genetically engineered mouse models NPA (**D**), NPAshCOL1A1 **(E)**, NPD **(F)** and NPDshCOL1A1 **(G)** gliomas (oncostreams are indicated with black arrowheads). Scale bar: 50 µm. 'Heatmap outputs illustrate the semantic segmentation probability heatmaps for each corresponding H&E image. Quantitative analysis of D-G is shown in Fig. 5K.

Fig. S13

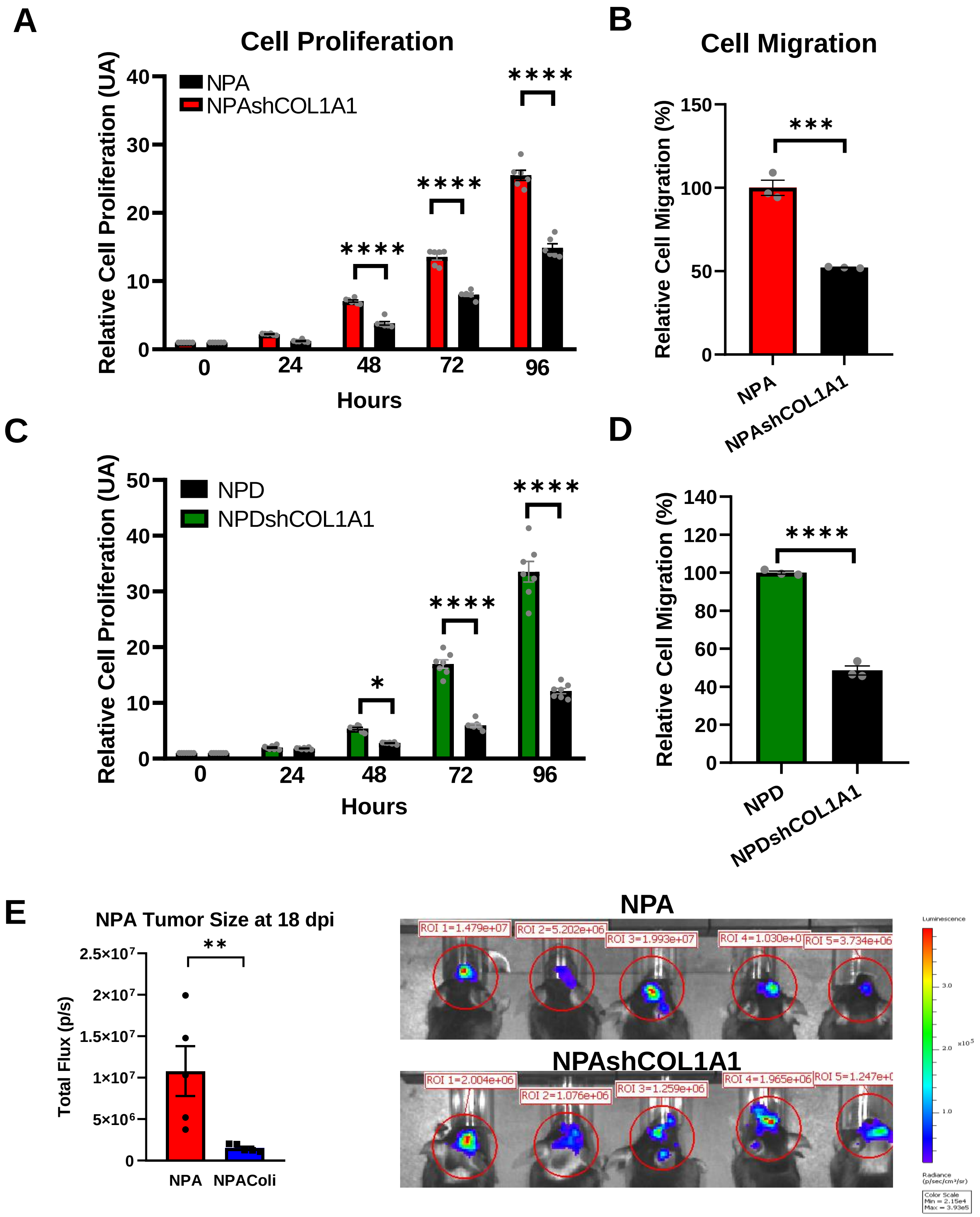

A
Cell Proliferation
Relative Cell Proliferation (UA)
NPA
NPAshCOL1A1
40
30
20
10
0
0
24
48
72
96
Hours
****
****
****
B
Cell Migration
Relative Cell Migration (%)
150
100
50
0
***
NPA
NPAshCOL1A1
C
Relative Cell Proliferation (UA)
NPD
NPDshCOL1A1
50
40
30
20
10
0
0
24
48
72
96
Hours
*
****
****
D
Relative Cell Migration (%)
140
120
100
80
60
40
20
0
****
NPD
NPDshCOL1A1
E
NPA Tumor Size at 18 dpi
Total Flux (p/s)
2.5x10^7
2x10^7
1.5x10^7
1x10^7
5x10^6
0
**
NPA
NPAColi
NPA
ROI 1=1.479e+07
ROI 2=5.202e+06
ROI 3=1.993e+07
ROI 4=1.030e+0
ROI 5=3.734e+06
NPAshCOL1A1
ROI 1=2.004e+06
ROI 2=1.076e+06
ROI 3=1.259e+06
ROI 4=1.965e+06
ROI 5=1.247e+0

Fig. S13. Downregulation of COL1A1 decreases cell proliferation and cell migration *in vitro*, and tumor growth *in vivo.*

Neurospheres obtained from genetically engineered mouse models (GEMM) of glioma controls (NPA and NPD) and COL1A1 downregulation (NPAshCOL1A1 and NPDshCOL1A1) were employed to determine cell proliferation and cell migration. **A** and **C)** Cell proliferation was assessed at 0, 24, 48 and 96 h (n=6 biologically independent samples) and was determined using Cell Titer-Glo Assay. Graphs present mean ±SEM. Two-way ANOVA with Sidak multiple comparisons. *p=0.0365, ****p<0.0001. **B** and **D)** Cell migration was measured after 15 h using transwell assay, and number of cells was determined using Cell Titer-Glo Assay. Data are expressed as percentage of migrating cells relative to the control. Graphs present mean ±SEM. Biologically independent samples n=3,***p=0.0005, ****p<0.0001. **E)** Tumor growth was determined measuring *in vivo* luminesce of C57BL mice bearing NPA or COL1A1 downregulation (NPAshCOL1A1) at 18 days post-implantation (n=5 biologically independent replicates). Graphs present mean ±SEM. Mann Whitney two-sided t-test, **P=0.0079.

Fig. S14

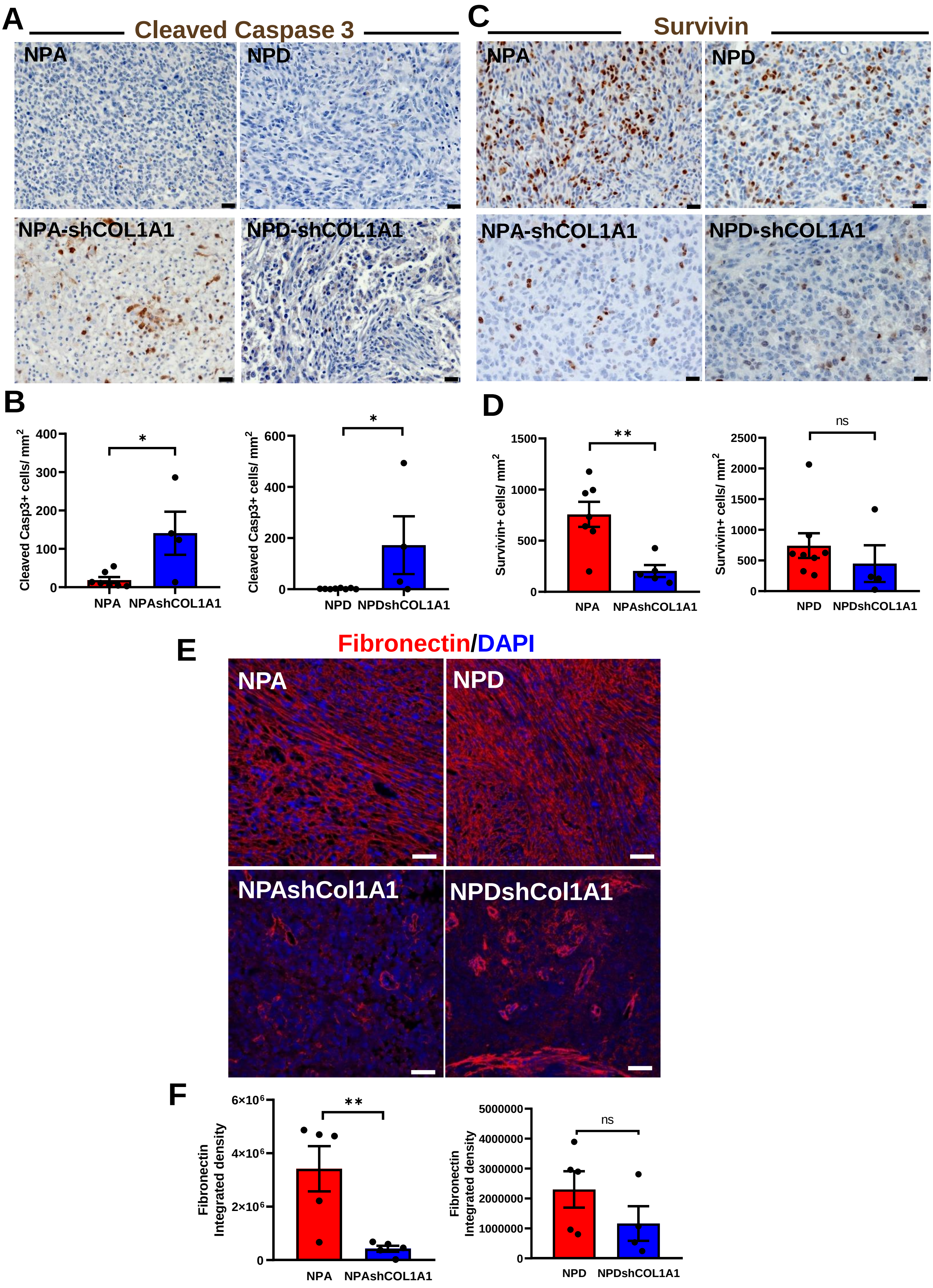
A
Cleaved Caspase 3
NPA
NPD
NPA-shCOL1A1
NPD-shCOL1A1
C
Survivin
NPA
NPD
NPA-shCOL1A1
NPD-shCOL1A1
B
Cleaved Casp3+ cells/ mm2
400
300
200
100
0
*
NPA
NPAshCOL1A1
Cleaved Casp3+ cells/ mm2
600
400
200
0
*
NPD
NPDshCOL1A1
D
Survivin+ cells/ mm2
1500
1000
500
0
**
NPA
NPAshCOL1A1
Survivin+ cells/ mm2
2500
2000
1500
1000
500
0
ns
NPD
NPDshCOL1A1
E
Fibronectin/DAPI
NPA
NPD
NPAshCol1A1
NPDshCol1A1
F
Fibronectin Integrated density
6×10⁶
4×10⁶
2×10⁶
0
**
NPA
NPAshCOL1A1
Fibronectin Integrated density
5000000
4000000
3000000
2000000
1000000
0
ns
NPD
NPDshCOL1A1

Fig. S14. COL1A1 genetic downregulation induced changes in apoptosis and decreased the expression of the mesenchymal marker fibronectin.

Immunohistochemical analysis (**A** and **C**) on genetically engineered mouse models (GEMM) of glioma controls (NPA and NPD) and COL1A1 downregulation (NPAshCOL1A1 and NPDshCOL1A1). **A)** Representative images of Cleaved Caspase 3 expression (DAB). Scale bar: 20 µm. **B)** Bar graphs represent Cleaved Caspase 3+ cells quantification (number of cell/mm^2) using QuPath positive cells detection. Graphs present mean ±SEM, (NPA: n=7, NPAshCOL1A1: n=4, NPD: n=8, NPDshCOL1A1: n=4), Mann Whitney two-sided t-test, NPA vs NPAshCOL1A1 *p=0.0424. Two-sided t-test, NPD vs NPDshCOL1A1 *p=0.0484. **C)** Representative images of Survivin expression (DAB). **D)** Bar graphs represent Survivin+ cells quantification (number of cell/mm^2) using QuPath positive cells detection. Graphs present mean ±SEM, (NPA: n=7, NPAshCOL1A1: n=5, NPD: n=8, NPDshCOL1A1: n=4), two-sided t-test, **p=0.005, ns: no significant. **E)** Immunofluorescence analysis on GEMM of glioma controls (NPA and NPD) and COL1A1 downregulation (NPAshCOL1A1 and NPDshCOL1A1). Representative images of Fibronectin expression in red (Alexa 555) and nuclei in blue (DAPI). Scale bar: 50 µm**. F)** Bar graphs represent Fibronectin quantification in terms of fluorescence integrated density. Graphs present mean ±SEM, (NPA: n=5, NPAshCOL1A1: n=5, NPD: n=5, NPDshCOL1A1: n=4), two-sided t-test, **p=0.0079, ns: no significant.

Fig. S15

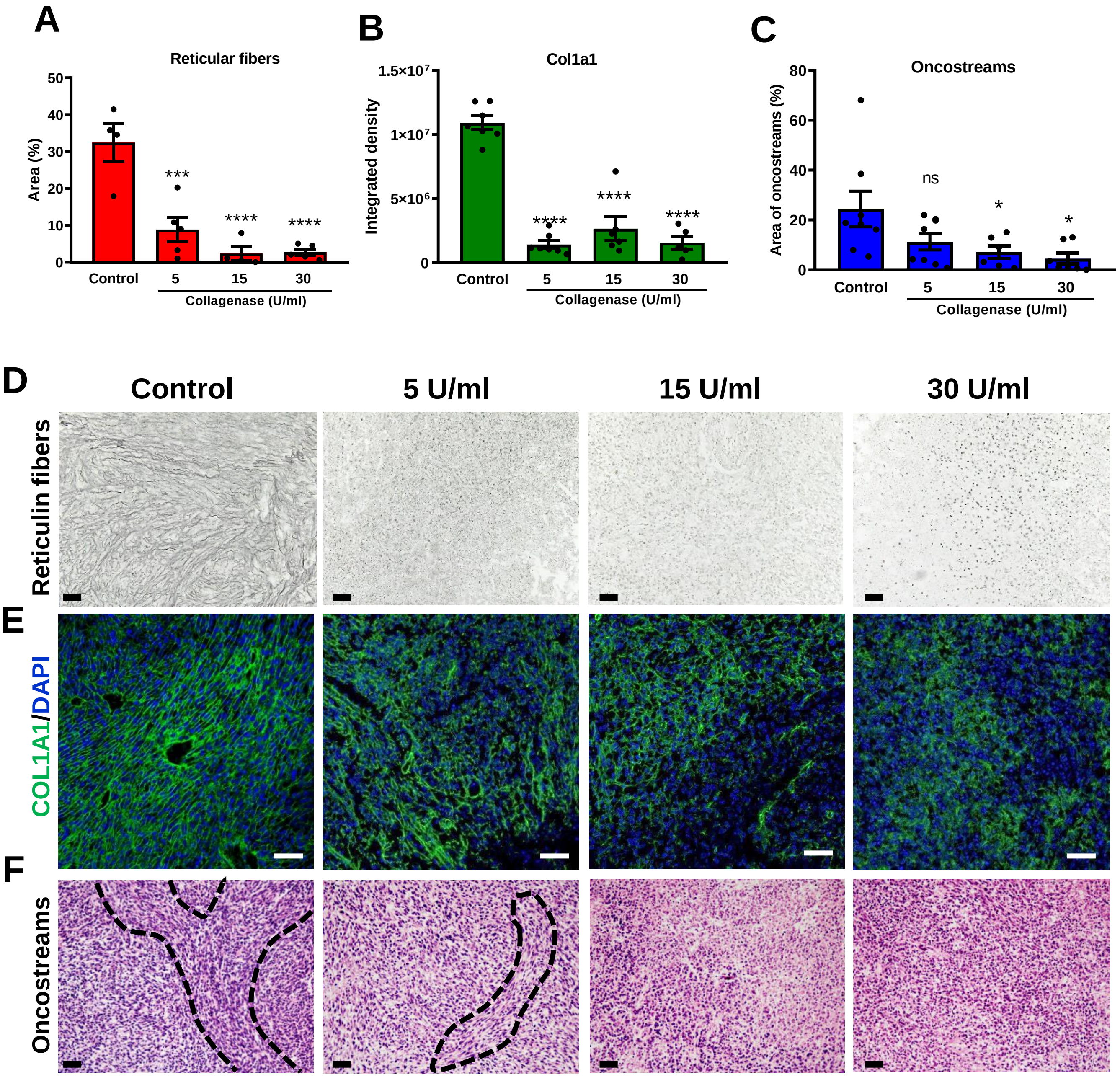

Fig. S15. Collagenase treatment depletes deposited collagen fibers and eliminated oncostream mesenchymal fascicles. A-F) Explant glioma brain slides were treated with different concentrations of collagenase (5 U/ml, 15 U/ml and 30 U/ml) for 48 hours. **A)** Bar graphs show the quantification of reticular fibers (Reticulin Silver Plating Kit acc. to Gordon & Sweets). Graphs present mean ±SEM. Control: n=8, 5 U/ml: n=8, 15 U/ml: n=6, 30 U/ml: n=7. One-way ANOVA, ***p=0.003, ****p<0.0001. **B)** Bar graphs represent COL1A1 quantification in terms of fluorescence integrated density. Error bars represent ±SEM. Control: n=7, 5 U/ml: n=7, 15 U/ml: n=6, 30 U/ml: n=5. One-way ANOVA, ****p<0.0001. **C)** Identification and quantification of oncostream areas using Image J. Graphs present mean ±SEM. Control: n=8, 5 U/ml: n=8, 15 U/ml: n=6, 30 U/ml: n=7. One-way ANOVA, Control vs 15 U/ml *p=0.0433, Control vs 30 U/ml *p=0.0133, ns: no significant. **D-F)** Representative images display reticular fiber expression and organization (**D**); COL1A1 expression and organization in green (Alexa 488) and nuclei in blue (DAPI) (**E**); and Hematoxylin and Eosin stained paraffin sections (**F**). Images (20x objective) Scale bars: 50 μm.

Fig. S16

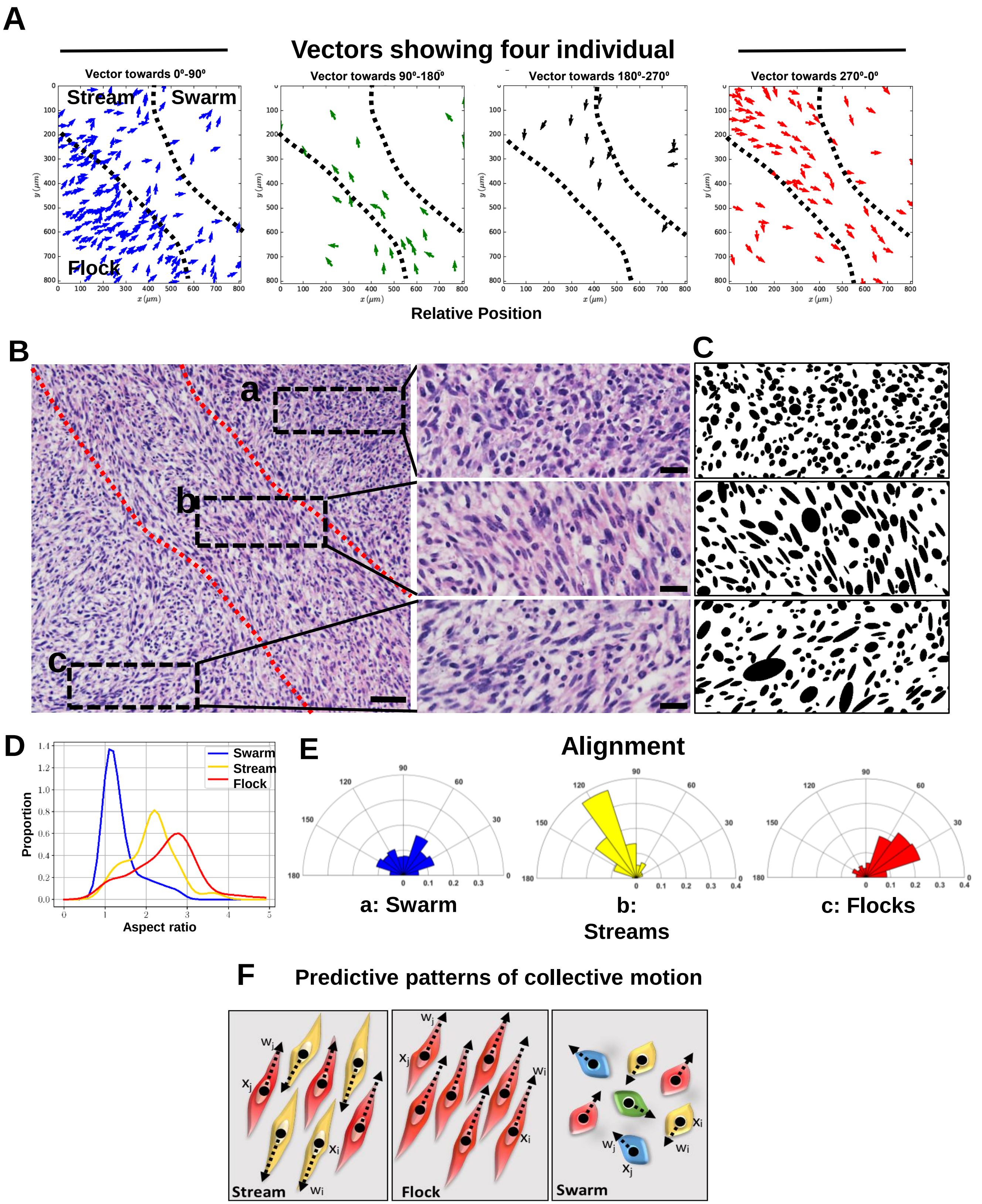
A
Vectors showing four individual
Vector towards 0º-90º
Vector towards 90º-180º
Vector towards 180º-270º
Vector towards 270º-0º
Stream
Swarm
Flock
Relative Position
B
a
b
c
C
D
Swarm
Stream
Flock
Proportion
Aspect ratio
E
Alignment
a: Swarm
b: Streams
c: Flocks
F
Predictive patterns of collective motion
Stream
Flock
Swarm

Fig. S16. Dynamic patterns are aligned to cellular morphology and histological features in gliomas. A) Vectors indicating average direction per cell for each area (from Fig. 7B-D). **B)** Hematoxylin and Eosin (H&E) staining of a 5 µm microtome section taken from an explant slice culture glioma model used for confocal imaging and shown in **Fig. 7B.** Dotted white lines define different zones which display different dynamic patterns in **Fig. 7D**, i.e., a=*swarm*, b=stream and c=flock, and boxes are shown at higher power to the right. This experiment was repeated at least three times. Scale bar: 50 µm. Inset scale bar: 20 µm **C)** Masks generated by particle analysis in Image J were used to calculate the alignments shown in Fig. 2D. **D)** Histogram of cell eccentricity analysis of organotypic H&E stained glioma sections. Aspect ratio of cells are shown for a swarm (blue; ~1), a stream (yellow; >2) and a flock (red; >2). **E)** Alignment analysis of cells: Angle histogram plots show areas of high proportion of aligned cells (stream and flock) (narrow range of angle orientation), and areas of no preferred orientation (swarm). **F)** Dynamic self-organizing collective motion patterns predicted by our *in silico* agent-based modeling, in which cell eccentricity and density determine pattern formation: 1. Stream (↑↓), 2. Flock (↑↑), 3. Swarm (↕↔). Figure was created by the authors.

Fig. S17

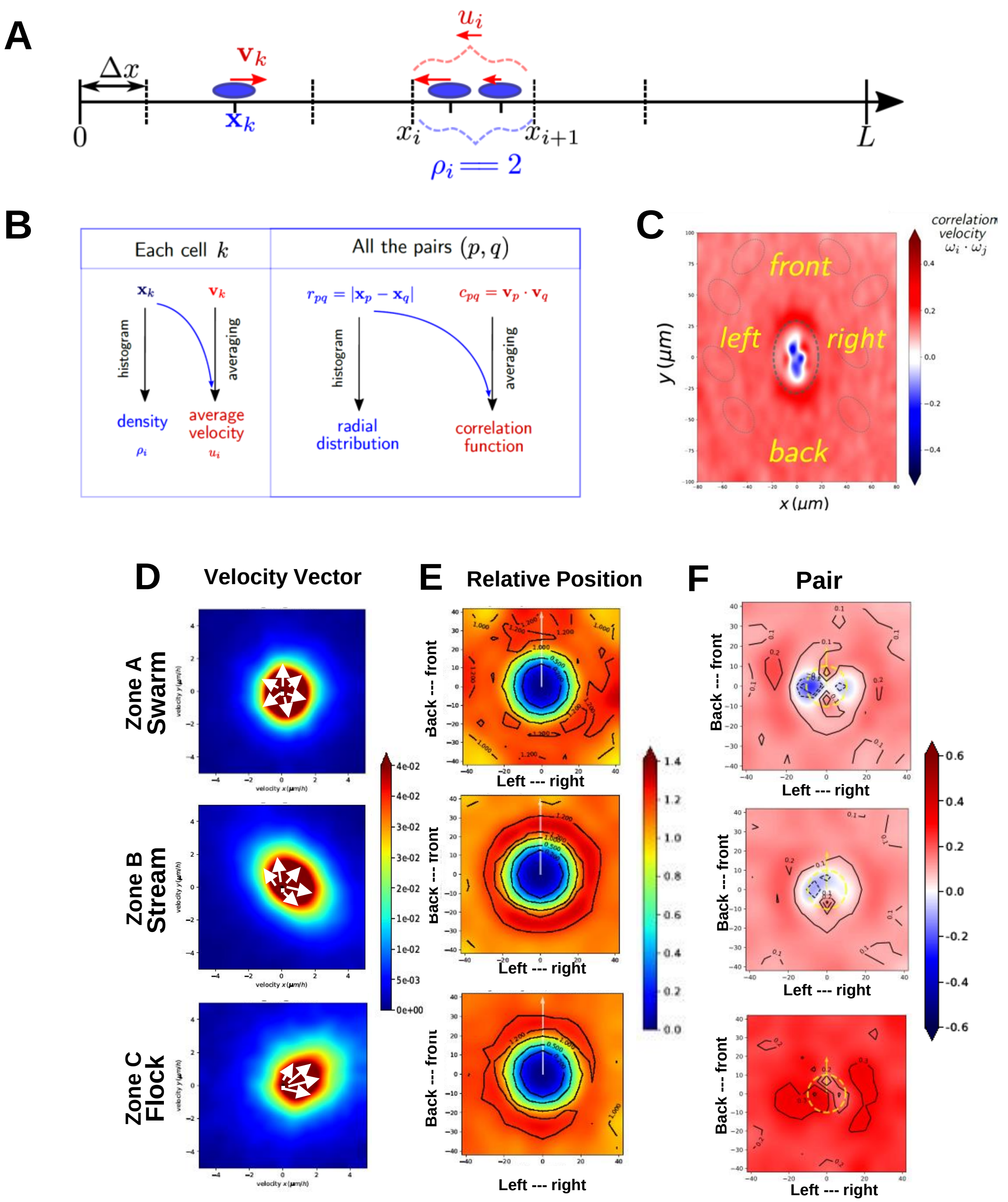

A
Δx
$\mathbf{v}_k$
$\mathbf{x}_k$
u_i
x_i
x_{i+1}
$\rho_i = 2$
0
L
B
Each cell k
All the pairs (p, q)
$\mathbf{x}_k$
$\mathbf{v}_k$
histogram
averaging
density
ρ_i
average velocity
u_i
$r_{pq} = |\mathbf{x}_p - \mathbf{x}_q|$
$c_{pq} = \mathbf{v}_p \cdot \mathbf{v}_q$
radial distribution
correlation function
C
front
left
right
back
y (μm)
x (μm)
correlation velocity
$\omega_i \cdot \omega_j$
D
Velocity Vector
E
Relative Position
F
Pair
Zone A Swarm
Zone B Stream
Zone C Flock
Back --- front
Left --- right

Fig. S17: Statistical analysis performed to study glioma dynamics.

A) Correlation functions: estimation of the density ρ_i and average velocity u_i. **B)** Radial distribution and correlation function are estimated in the same way as density and average velocity, but we use pairwise information (e.g., distances between neighbors). **C)** Heat map plot of the pair wise correlation of the velocity. We estimated the correlation depending on the position of a nearby x_j in different positions (front-to-back and left-to-right). Positive correlation (+) indicates cells are moving in same directions (red) and negative correlation (-) cells are moving in opposite directions (blue). **D)** Heat map plot of the distribution of the velocity vector in different zones of the tumor core. Velocity is shown in the x-axis and y-axis in µm/hr. **E)** Frequency for each zone of the relative position between two cells. In the frame of reference of one cell, we estimated the probability of having another cell, x_j, nearby. **F)** Pair-wise correlation with nearby neighbors. Depending on the relative position $\mathbf{x}_j$-$\boldsymbol{x}_j$, we estimate the correlation between the velocity directions $\boldsymbol{\omega}_i$ and $\boldsymbol{\omega}_j$. Axes indicate cell position and distance in µm of the neighboring cells. Dotted yellow line show the average size of the centered cell.

Fig. S18

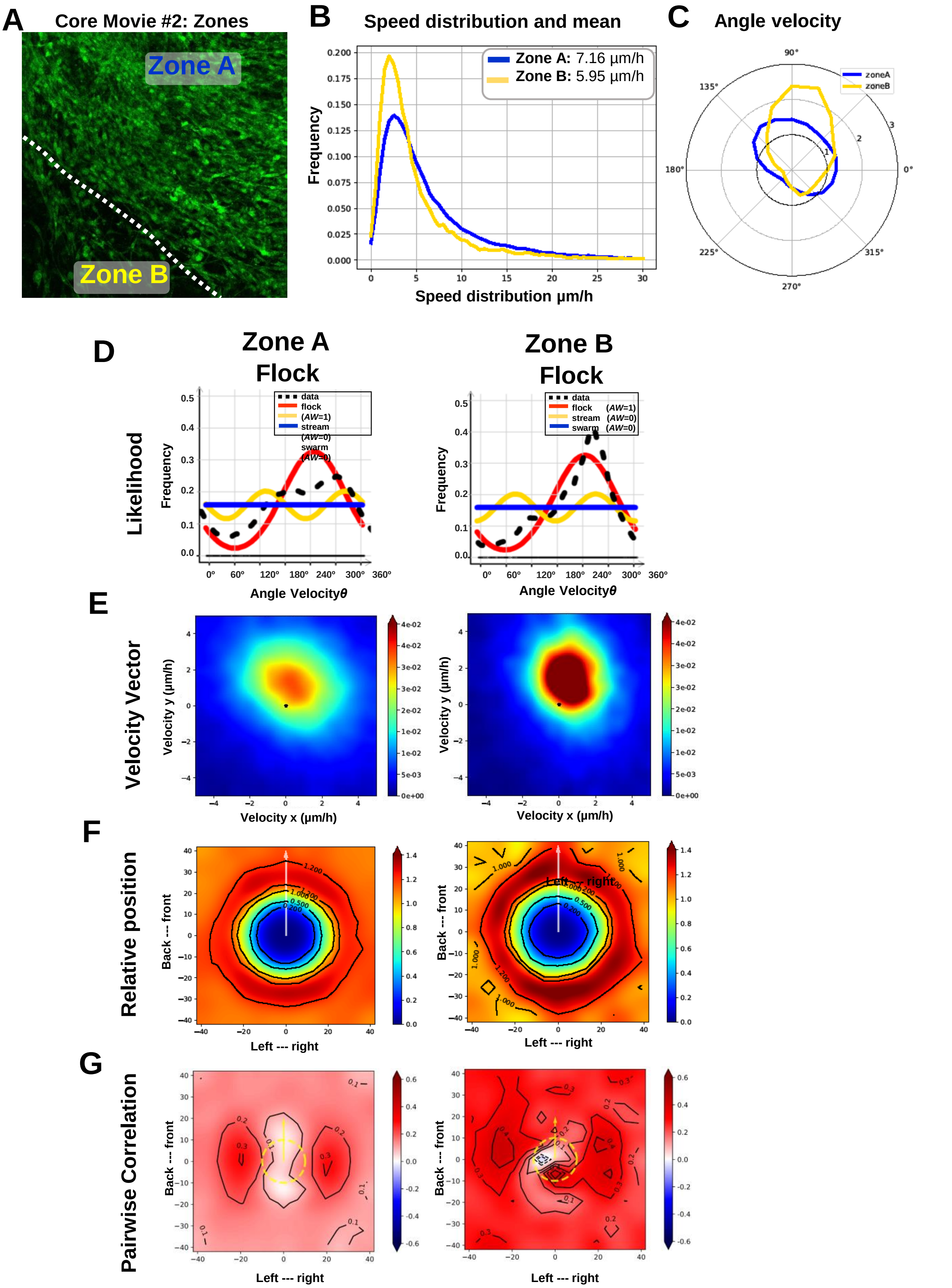
A
Core Movie #2: Zones
Zone A
Zone B
B
Speed distribution and mean
Zone A: 7.16 μm/h
Zone B: 5.95 μm/h
Frequency
Speed distribution μm/h
C
Angle velocity
zoneA
zoneB
D
Zone A Flock
Zone B Flock
Likelihood
data
flock (AW=1)
stream (AW=0)
swarm (AW=0)
Frequency
Angle Velocityθ
E
Velocity Vector
Velocity y (μm/h)
Velocity x (μm/h)
F
Relative position
Back --- front
Left --- right
G
Pairwise Correlation
Back --- front
Left --- right

Fig. S18: Collective dynamic patterns observed by confocal imaging within the glioma tumor core: analysis of core movie #2. A) Statistical analysis of different regions of the movie, Zone A and Zone B, illustrated on a representative time-lapse confocal still image (green(GFP+):tumor cells). **B)** Speed distribution (*µm/h*) in Zone A (blue) and B (yellow). Inner panel shows mean speed for each zone. **C)** Angle Velocity distribution analysis (θ) performed by zones. **D)** Likelihood analysis histograms to classify dynamic pattern formation. Zone A: flock, Zone B: flock. AW: Akaiki Weight. AW=0 or AW=1. **E)** Heat map of the distribution of velocity vectors in each zone. **F)** Histogram plot showing relative position with nearby neighbors within each zone. X and Y axes are in µm. Scale bar shows frequency represented in colors. **G)** Histograms of pair-wise correlation with nearby neighbors for each zone. X and Y axes are in µm. Scale bar shows correlation represented as colors.

Fig. S19

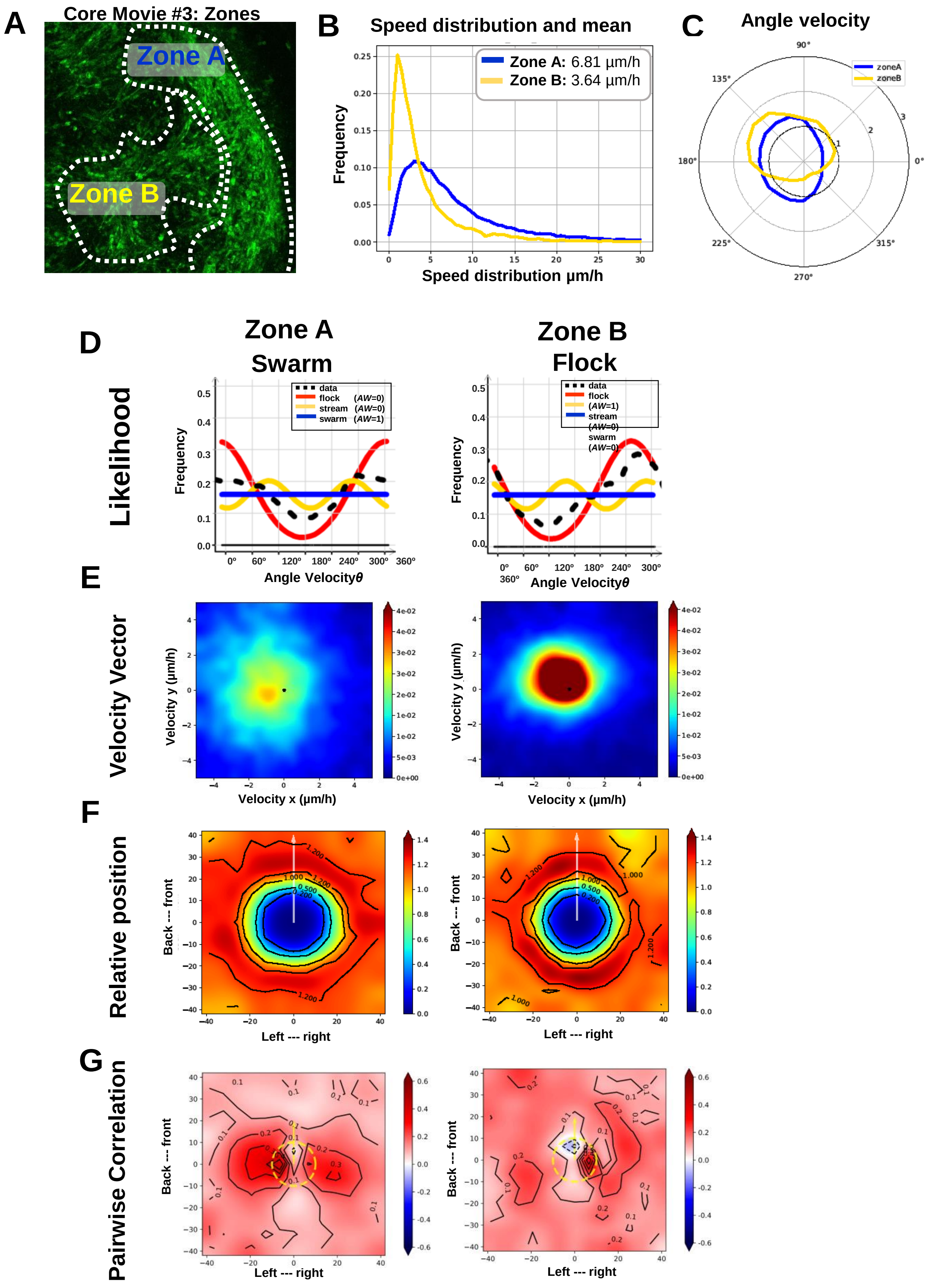
A
Core Movie #3: Zones
Zone A
Zone B
B
Speed distribution and mean
Zone A: 6.81 µm/h
Zone B: 3.64 µm/h
Frequency
Speed distribution µm/h
C
Angle velocity
zoneA
zoneB
90°
135°
180°
225°
270°
315°
0°
D
Likelihood
Zone A
Swarm
Zone B
Flock
data
flock (AW=0)
stream (AW=0)
swarm (AW=1)
flock
(AW=1)
stream
(AW=0)
swarm
(AW=0)
Frequency
Angle Velocityθ
E
Velocity Vector
Velocity y (µm/h)
Velocity x (µm/h)
F
Relative position
Back --- front
Left --- right
G
Pairwise Correlation
Back --- front
Left --- right

Fig. S19: Collective dynamic patterns observed by confocal imaging within the glioma tumor core: analysis of core movie #3. A) Statistical analysis of different regions of the movie, Zone A and Zone B, illustrated on a representative time-lapse confocal still image (green(GFP+):tumor cells). **B)** Speed distribution (*μm/hr*) in Zone A (blue) and B (yellow). Inner panel shows mean of the speed for each zone. **C)** Angle Velocity distribution analysis (θ) performed by zones. **D)** Likelihood analysis histograms to classify dynamic pattern formation. Zone A: flock, Zone B: flock. AW: 0 or AW:1. **E)** Heat map plot of the distribution of the velocity vector in each zone. **F)** Histogram plot showing interposition with nearby neighbor for each zone. X and Y axes are in µm. Scale bar shows frequency represented in colors. **G)** Histograms of pair-wise correlation with nearby neighbors for each zone. X and Y axes are in µm. Scale bar shows correlation represented in colors.

Fig. S20

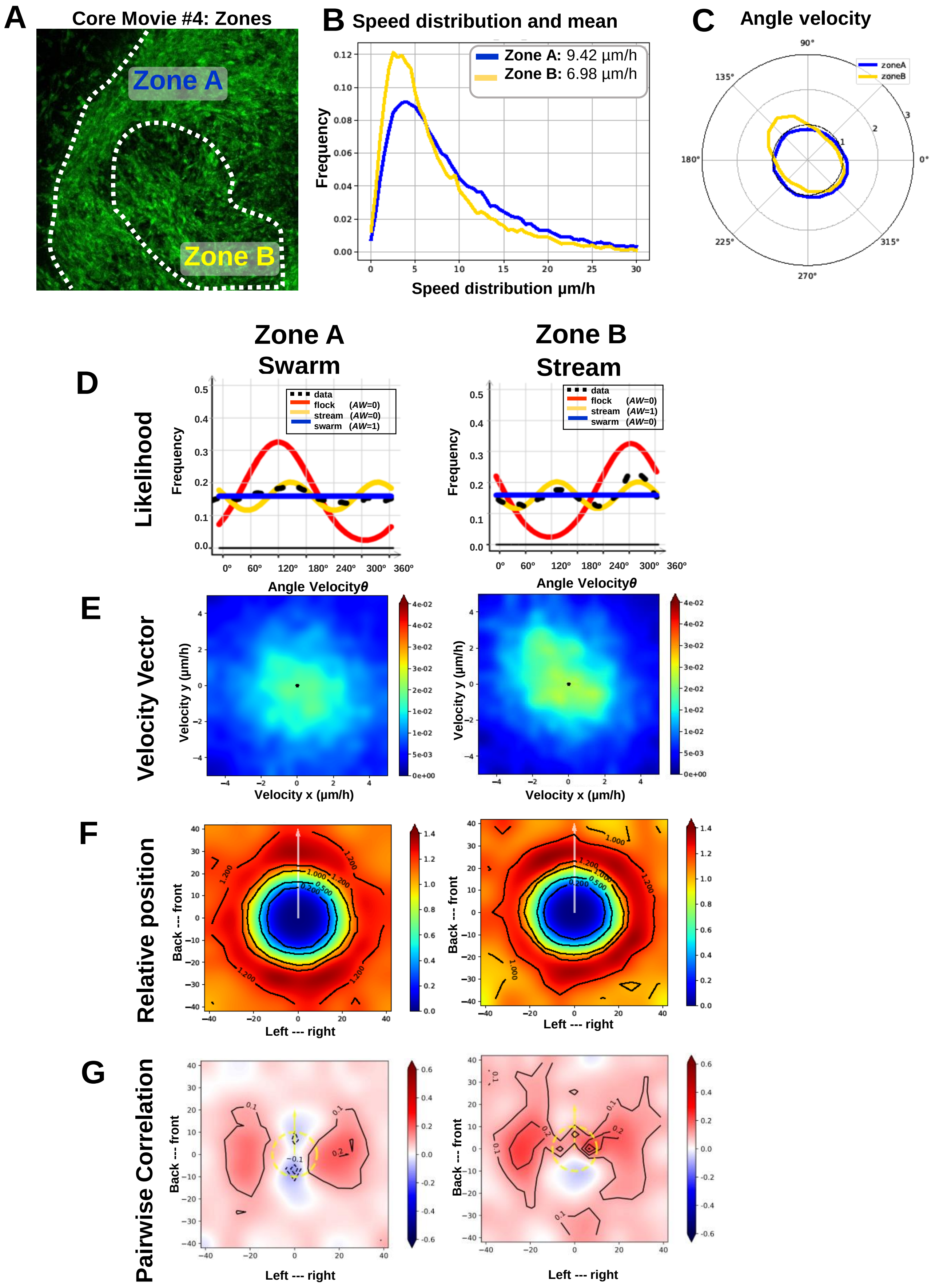
A
Core Movie #4: Zones
Zone A
Zone B
B
Speed distribution and mean
Zone A: 9.42 µm/h
Zone B: 6.98 µm/h
Frequency
Speed distribution µm/h
C
Angle velocity
zoneA
zoneB
Zone A
Swarm
Zone B
Stream
D
Likelihood
data
flock (AW=0)
stream (AW=0)
swarm (AW=1)
flock (AW=0)
stream (AW=1)
swarm (AW=0)
Frequency
Angle Velocityθ
E
Velocity Vector
Velocity y (µm/h)
Velocity x (µm/h)
F
Relative position
Back --- front
Left --- right
G
Pairwise Correlation
Back --- front
Left --- right

Fig. S20: Collective dynamic patterns observed by confocal imaging within the glioma tumor core: analysis of core movie #4. A) Statistical analysis of different regions of the movie, Zone A and Zone B, illustrated on a representative time-lapse confocal still image (green(GFP+):tumor cells). **B)** Speed distribution (*µm/hr*) in Zone A (blue) and B (yellow). Inner panel shows mean of the speed for each zone. **C)** Angle Velocity distribution analysis (θ) performed by zones. **D)** Likelihood analysis histograms to classify dynamic pattern formation. Zone A: swarm, Zone B: Stream. AW:0 or AW:1. **E)** Heat map plot of the distribution of the velocity vector in each zone. **F)** Histogram plot showing interposition with nearby neighbor for each zone. X and Y axes are in µm. Scale bar shows frequency represented in colors. **G)** Histograms of pair-wise correlation with nearby neighbors for each zone. X and Y axes are in µm. Scale bar shows correlation represented in colors.

Fig. S21

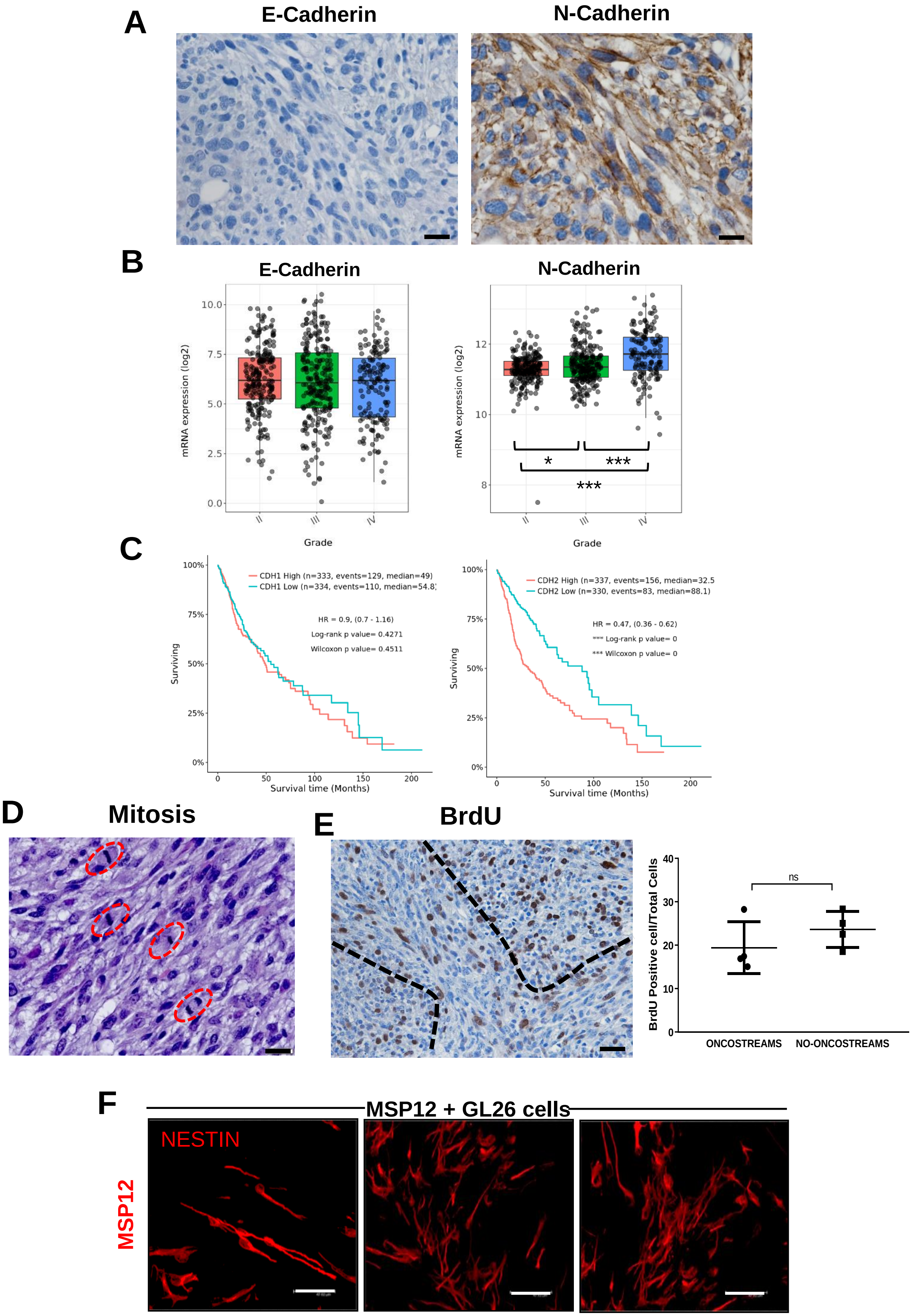
A
E-Cadherin
N-Cadherin
B
E-Cadherin
N-Cadherin
mRNA expression (log2)
Grade
II
III
IV
*
***
***
C
CDH1 High (n=333, events=129, median=49)
CDH1 Low (n=334, events=110, median=54.8)
HR = 0.9, (0.7 - 1.16)
Log-rank p value= 0.4271
Wilcoxon p value= 0.4511
CDH2 High (n=337, events=156, median=32.5
CDH2 Low (n=330, events=83, median=88.1)
HR = 0.47, (0.36 - 0.62)
*** Log-rank p value= 0
*** Wilcoxon p value= 0
Surviving
Survival time (Months)
D
Mitosis
E
BrdU
BrdU Positive cell/Total Cells
ns
ONCOSTREAMS
NO-ONCOSTREAMS
F
MSP12 + GL26 cells
NESTIN
MSP12

Fig. S21: Characterization of Oncostream' s cellular functions. A) Immunohistochemistry analysis of E-cadherin and N-cadherin expression within NPA gliomas. Gliomas are negative for E-cadherin and positive for N-cadherin both within and outside oncostream structures. **B)** mRNA expression analysis of N-cadherin and E-cadherin comparing glioma tumors of different grade using the TCGA-GBMLGG database. Graph shows the $\log_2$ mRNA expression levels. Centre line of the boxplot is the median, upper bound of the boxplot is the Q3 (75th percentile), the lower bound of the boxplot is the Q1(25th percentile), the upper whiskers is the maximum value of the data (within 1.5 times the interquartile range over the 75th percentile), and the lower whisker is the minimum value of the data (within 1.5 times the interquartile range under the 25th percentile). Statistical significance of the data set was calculated using One-way ANOVA test and multiple comparison Tukey test, *p=0.031, ****p<0.0001. (Grade II: n=226, Grade III: n=244, Grade IV: n=150). **C)** Kaplan-Meier survival curves of glioma patients comparing N-cadherin and E-cadherin high (red) vs low (blue) expression in glioma tumors (TCGA-GBMLGG database). All these data was analyzed from the Gliovis website (http://gliovis.bioinfo.cnio.es). **D)** Analysis of glioma cell mitosis within oncostreams in Hematoxylin and Eosin (H&E) stained sections. Mitosis is orientated in the same orientation of oncostreams. Red lines indicate mitosis. Experiment was performed in 5 independent tumor sections. **E)** Proliferation analysis comparing oncostream (dotted lines) with no-oncostream areas. Positive BrDu cells were counted by Image-J software. Scale bars: 50 µm. BrDu positive cells per total cells in the visual field were counted; n=4. Ten fields of each section were selected at random. Error bars represent ± SEM; paired two-sided t-test. **F)** Immunofluorescence images of human-nestin (red) highlighting MSP12 cells. MSP-12 cells were implanted with GL26-citrine cells. MSP12 cells adopt a bipolar structure only when aligned to GL26-citrine cells. Scale bar: 47.62 µm. This image complement Fig. 7K. Experiment was performed in 3 independent tumor samples as indicated in Fig. 7K.

Fig. S22

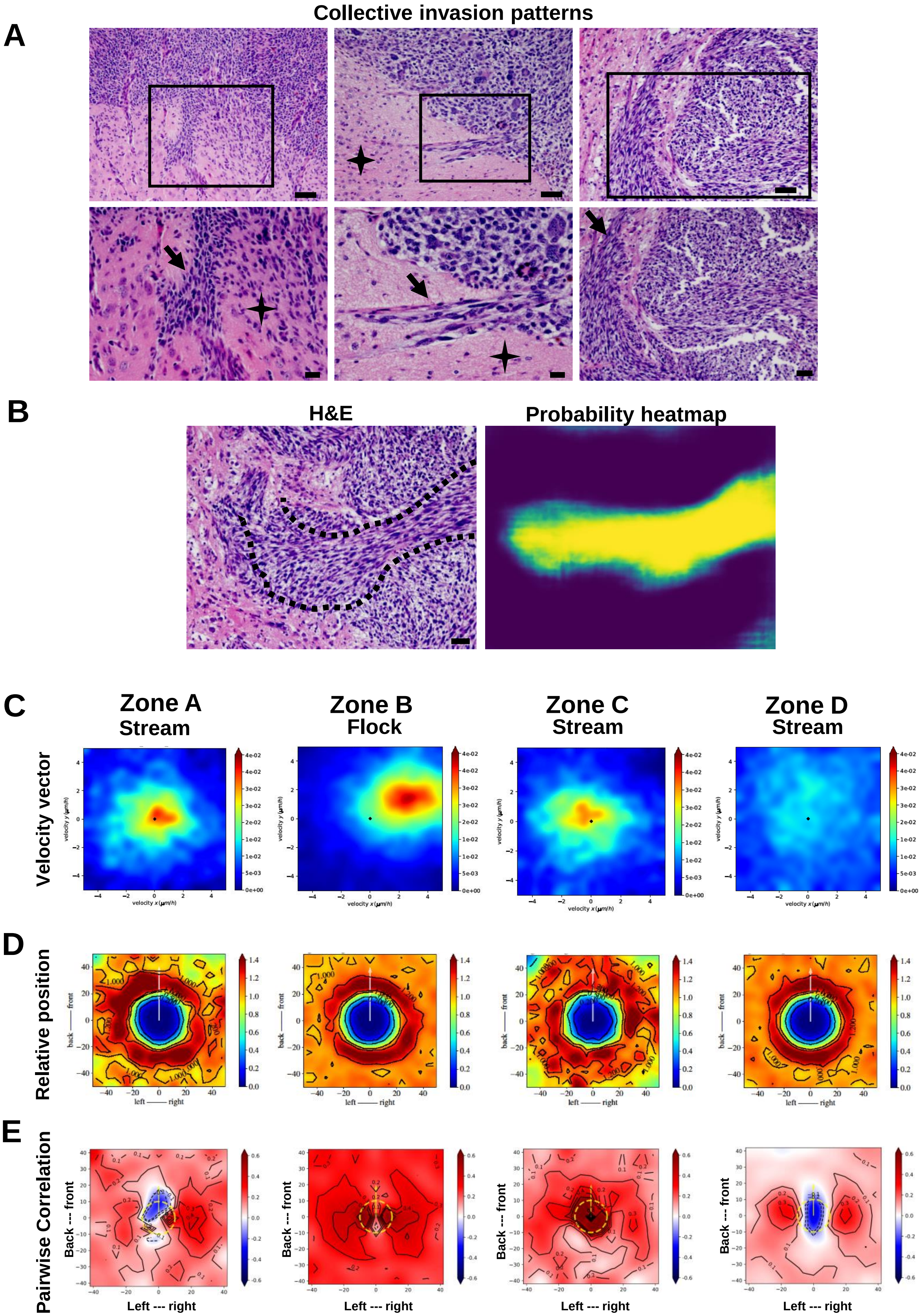
Collective invasion patterns
A
B
H&E
Probability heatmap
C
Zone A
Stream
Zone B
Flock
Zone C
Stream
Zone D
Stream
Velocity vector
velocity x (μm/h)
velocity y (μm/h)
D
Relative position
left —— right
back —— front
E
Pairwise Correlation
Left --- right
Back --- front

Fig. S22: Invasion analysis using confocal time-lapse imaging on the tumor border: Movie #5. A) Representative images of oncostream invasion (top row, black boxes) from Hematoxylin and Eosin (H&E) stained sections of GEMM of glioma. Arrows indicate oncostream collective invasion. Areas highlighted by black boxes are shown at higher magnification in the lower row. Stars indicate sites of single cell invasion. Experiment was performed in six independent tumor sections. Scale bars: 50 µm (top) and 20 µm (bottom). **B)** Representative images of oncostream invasion (dotted line indicates an oncostream at the tumor border) on H&E stained sections of NPA gliomas (left), and its detection by deep learning analysis (right). Experiment was performed in three independent tumor sections. Scale bar=20 µm. **C)** Heatmap plot of the distribution of velocity vectors in zones A, B, C and D of border movie #5 (shown in **Fig. 5)**. **D)** Histogram of relative cell positions in relation to nearby neighbors. For each cell, $x_{i,}$ we estimate the probability to find another cell, $x_{j,}$ nearby. Probability scale bars are represented by colors. Axes shows cells position left-to-right and front-to-back in µm. **E)** Pairwise correlation of motion with nearby neighbors. Scale bars of probability are represented by colors. Axes indicate cell position and distance in µm from neighboring cells. Dotted yellow line indicates the location of x_i.

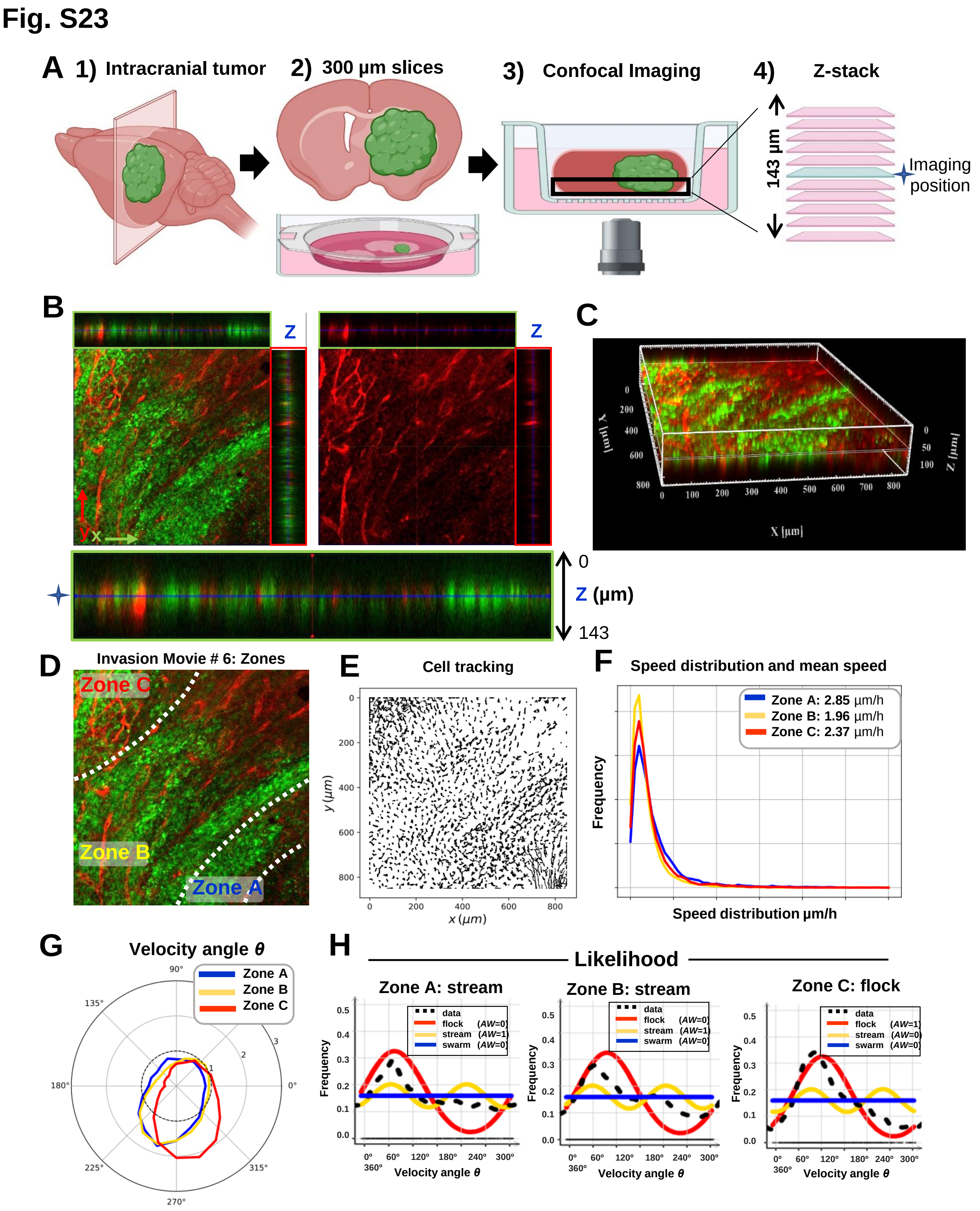
Fig. S23
A
1) Intracranial tumor
2) 300 μm slices
3) Confocal Imaging
4) Z-stack
143 μm
Imaging position
B
Z
Z
0
Z (μm)
143
C
X [μm]
Z [μm]
D
Invasion Movie # 6: Zones
Zone C
Zone B
Zone A
E
Cell tracking
y (μm)
x (μm)
F
Speed distribution and mean speed
Zone A: 2.85 μm/h
Zone B: 1.96 μm/h
Zone C: 2.37 μm/h
Frequency
Speed distribution μm/h
G
Velocity angle θ
Zone A
Zone B
Zone C
H
Likelihood
Zone A: stream
Zone B: stream
Zone C: flock
data
flock (AW=0)
stream (AW=1)
swarm (AW=0)
flock (AW=1)
stream (AW=0)
Frequency
Velocity angle θ

Fig. S23

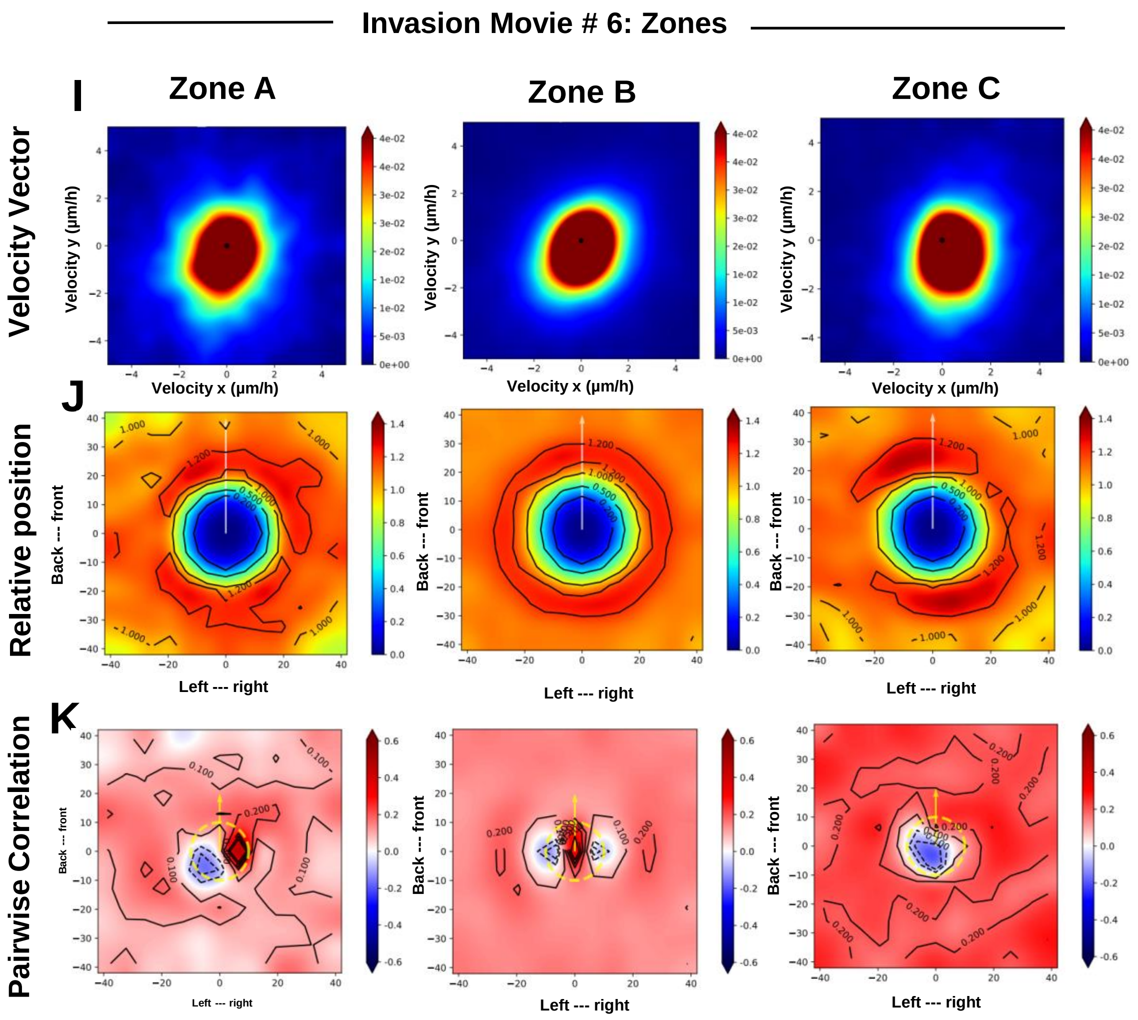

Invasion Movie # 6: Zones
I
Zone A
Zone B
Zone C
Velocity Vector
Velocity y (μm/h)
Velocity x (μm/h)
J
Relative position
Back --- front
Left --- right
K
Pairwise Correlation
Back --- front
Left --- right

Fig. S23. Brain tumor explant model setup and invasion analysis using time lapse-confocal imaging: Movie #6

A) Schematic representation of the experimental setup and site of imaging. 1) NPA-GFP+ cells were intracranially implanted, and brains from tdTomato mice bearing GFP+ tumors were isolated 19 days post-tumor implantation. 2) These brains were sliced into 300 µm thick coronal sections and transferred to a laminin-coated cell culture Insert. 3) Cell culture insets containing brain tumor slices were placed in the incubator chamber of an inverted Zeiss LSM880 laser scanning confocal microscope at 37 °C with a 5% CO2 atmosphere. The black rectangle represents the imaging area that extends from the bottom to ~143 µm; not being able to image further into the brain slice. 4) A high-resolution z-stack was obtained (dimensions of x=850.19, y=850.19, z=143.81). The location for time-lapse imaging was selected to fall at the middle of the z-stack (~70 µm) to avoid imaging at the bottom of the explant. Red fluorescent protein: normal brain parenchyma. Green fluorescent protein: tumor cells. (Created with BioRender.com) **B)** Orthogonal projections from z-stacks revealed the cross-sectional aspects of the tumor volume and the position of time-lapse confocal imaging of Movie #6, an NPA glioma. Green rectangles (on top) show the profile of the x-z plane. Red rectangles (on the right) show the profile of the y-z plane. The left image illustrates tumor cells (in green) and brain parenchyma (in red); the right image shows only the brain parenchyma. The larger green rectangle at the bottom of **(Fig. S23B)** illustrates the orthogonal z-stack projection of the x-z plane (from 0 to 143 µm) and the exact plane of imaging, in the middle of z plane (indicated by a star and a blue line). **C)** A high-resolution 3D z-stack spanning up to 143 µm depth (starting at the bottom of the brain slice) was acquired on a confocal microscope, imported into the Imaris viewer, and used to reconstruct this 3D image. 30 x-y frames from the brain's surface were taken at a depth increment of 4.96 µm at a resolution of 1024x1024 pixels. XYZ axes of the 3D image are shown in white (850x850x143 µm), and the yellow line shows the exact imaging plane for time-lapse data acquisition *in vivo* (at ~70 µm depth). Red fluorescent protein: normal brain parenchyma. Green fluorescent protein: tumor cells. **D)** Representative time-lapse scanning confocal image of an NPA glioma growing at the tumor border, and showing Zones A, B, C. **E)** Tracking analysis of individual cell paths using the TrackMate plugin from Image-J. **F)** Histogram of speed distribution and mean speed (*µm/h*) of Zones A, B and C. **G)** Angle Velocity distribution analysis (*θ*) performed by zones. The plot shows the overall direction and magnitude of cell movement. **H)** Likelihood analysis of the dynamic patterns at the tumor border. The frequency distribution *ρ flock* (red), *ρ stream* (yellow) and *ρ swarm* (blue) are shown. The estimation of the black line (data) uses a *non-parametric* estimation. AW: 0 or AW:1. **I)** Heat map plot of the distribution of the velocity vectors in each zone. **J)** Histogram plot showing the relative position with nearby neighbors for each zone; *x* and *y* axes are in µm. Bars to the right of each figure indicate probability values in color. **K)** Histograms of pairwise correlations with nearby neighbors for each zone; *x* and *y* axes are in µm. Bars to the right of each figure indicate correlation values in colors.

Fig. S24

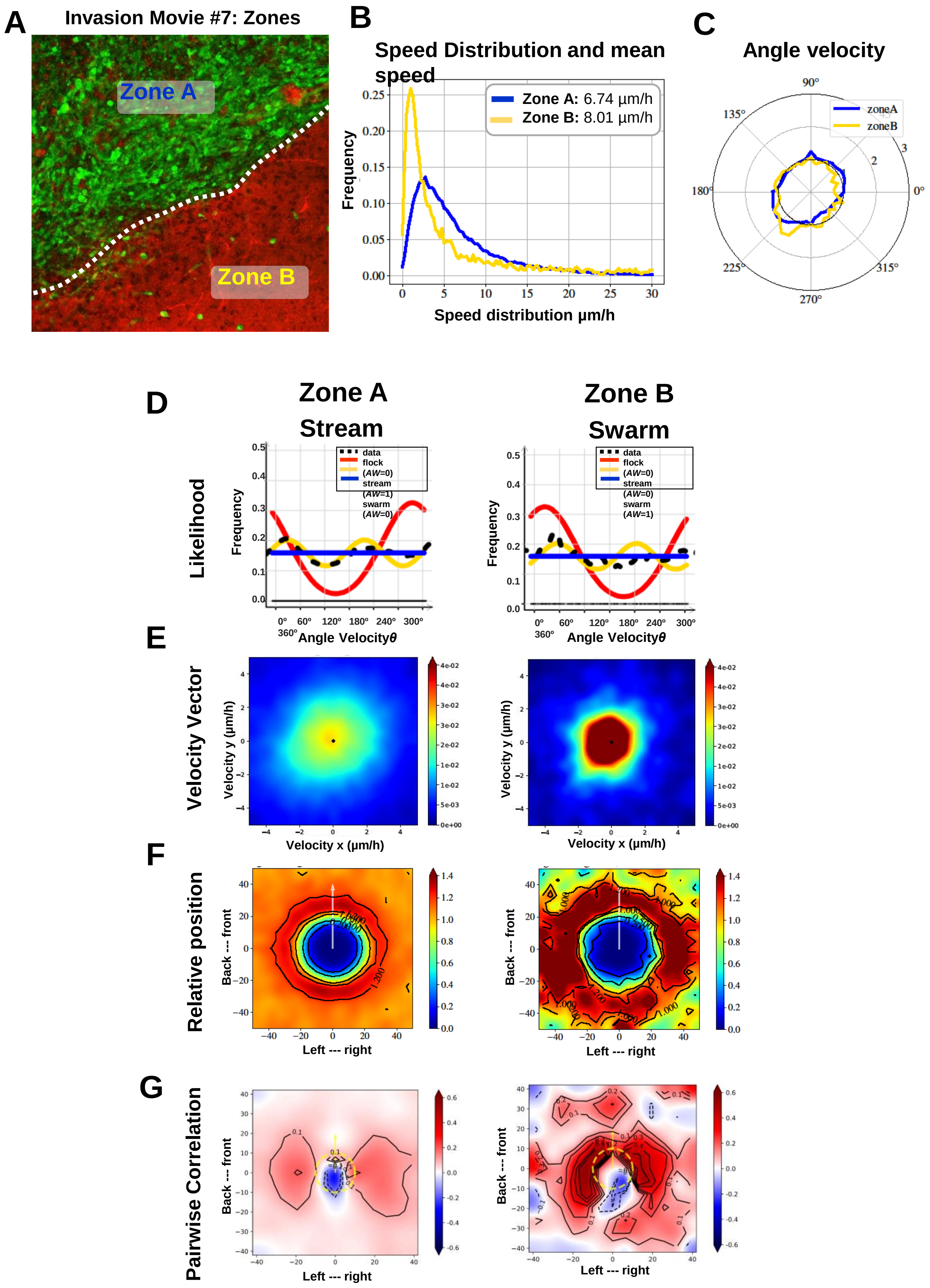
A
Invasion Movie #7: Zones
Zone A
Zone B
B
Speed Distribution and mean speed
Zone A: 6.74 µm/h
Zone B: 8.01 µm/h
Frequency
Speed distribution µm/h
C
Angle velocity
zoneA
zoneB
D
Zone A
Stream
Zone B
Swarm
Likelihood
data
flock (AW=0)
stream (AW=1)
swarm (AW=0)
data
flock (AW=0)
stream (AW=0)
swarm (AW=1)
Frequency
Angle Velocity θ
E
Velocity Vector
Velocity y (µm/h)
Velocity x (µm/h)
F
Relative position
Back --- front
Left --- right
G
Pairwise Correlation
Back --- front
Left --- right

Fig. S24: Invasion analysis using confocal time-lapse imaging on the tumor border: Movie #7) A) Labeling of different regions of the tumor border for movie #6. Representative time-lapse confocal images subdivided into dynamic Zones A and B. The image shows tumor cells (green) and brain parenchyma (red). **B)** Speed distribution (*μm/h*) in Zone A (blue) and B (yellow). Inner panel shows mean speed for each zone. **C)** Angle Velocity distribution analysis (θ) performed by zones. **D)** Likelihood analysis histograms classify dynamic motion patterns. Zone A: stream, Zone B: swarm. AW:0 or AW:1. **E)** Heat map plot of the distribution of the velocity vectors in each zone. **F)** Histogram plot showing the relative position with nearby neighbors for each zone. *x* and *y* axes are in μm. Bars to the right of each figure indicate probability values in color. **G)** Histograms of pairwise correlations with nearby neighbors for each zone. *x* and *y* axes are in μm. Bars to the right of each figure indicate correlation values in colors.

Fig. S25

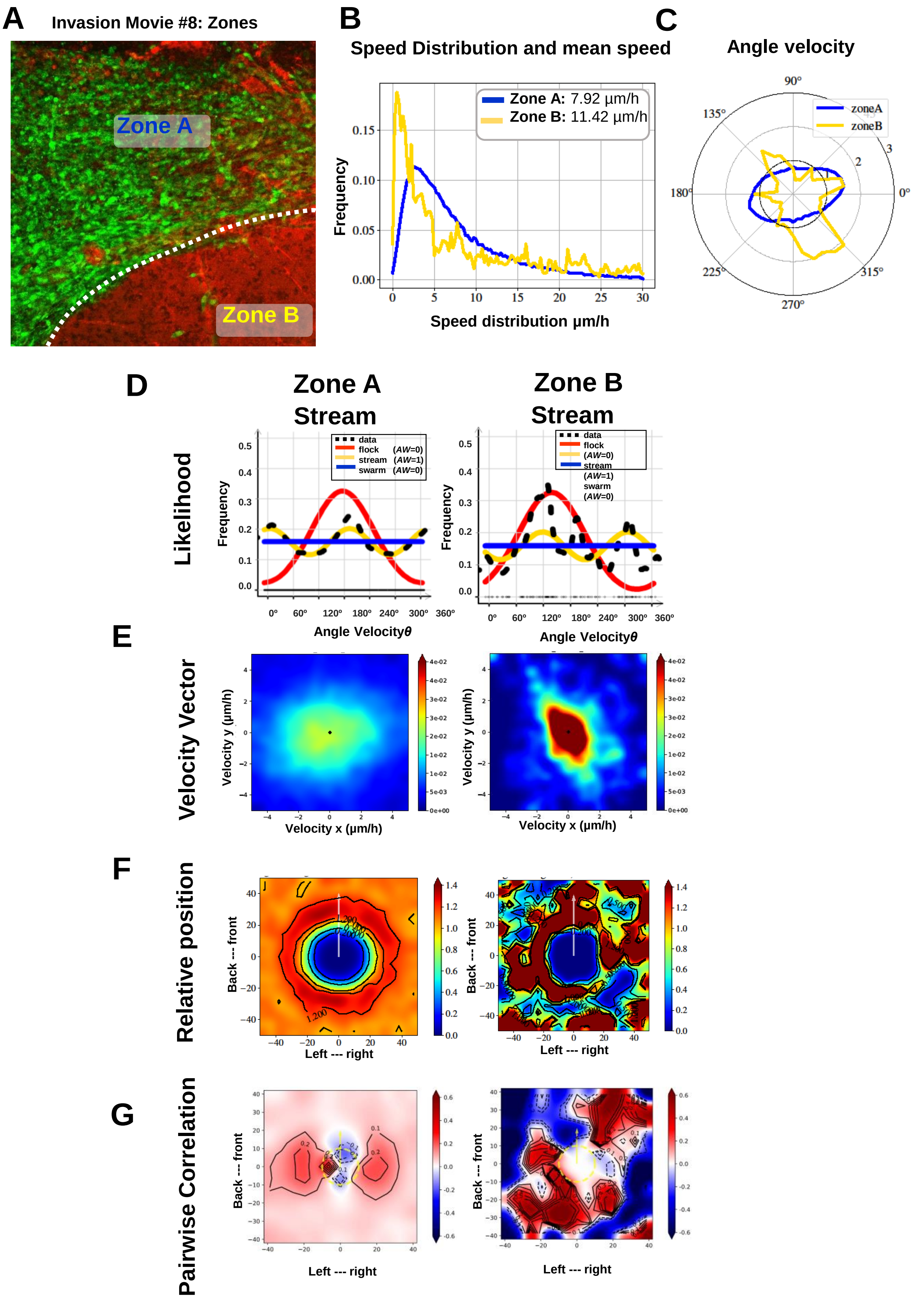
A
Invasion Movie #8: Zones
Zone A
Zone B
B
Speed Distribution and mean speed
Zone A: 7.92 µm/h
Zone B: 11.42 µm/h
Frequency
Speed distribution µm/h
C
Angle velocity
zoneA
zoneB
D
Zone A
Stream
Zone B
Stream
Likelihood
data
flock (AW=0)
stream (AW=1)
swarm (AW=0)
Frequency
Angle Velocityθ
E
Velocity Vector
Velocity y (µm/h)
Velocity x (µm/h)
F
Relative position
Back --- front
Left --- right
G
Pairwise Correlation
Back --- front
Left --- right

Fig. S25: Invasion analysis using confocal time-lapse imaging of the tumor border: Movie #8. A) Labeling of different regions of the tumor border for movie #7. Representative time-lapse confocal images subdivided into dynamic Zones A and B. The image shows tumor cells (green) and brain parenchyma (red). **B)** Speed distribution (*μm*/*h*) in Zone A (blue) and B (yellow). Inner panel shows mean speed for each zone. **C)** Angle Velocity distribution analysis (θ) performed by zones. **D)** Likelihood analysis histograms classify dynamic motion patterns. Zone A: stream, Zone B: stream. Akaiki Weight; AW:0 or AW:1. **E)** Heat map plot of the distribution of the velocity vectors in each zone. **F)** Histogram plot showing the relative position with nearby neighbors for each zone. *x* and *y* axes are in μm. Bars to the right of each figure indicate probability values in color. **G)** Histograms of pairwise correlations with nearby neighbors for each zone. *x* and *y* axes are in μm. Bars to the right of each figure indicate correlation values in colors.

Fig. S26

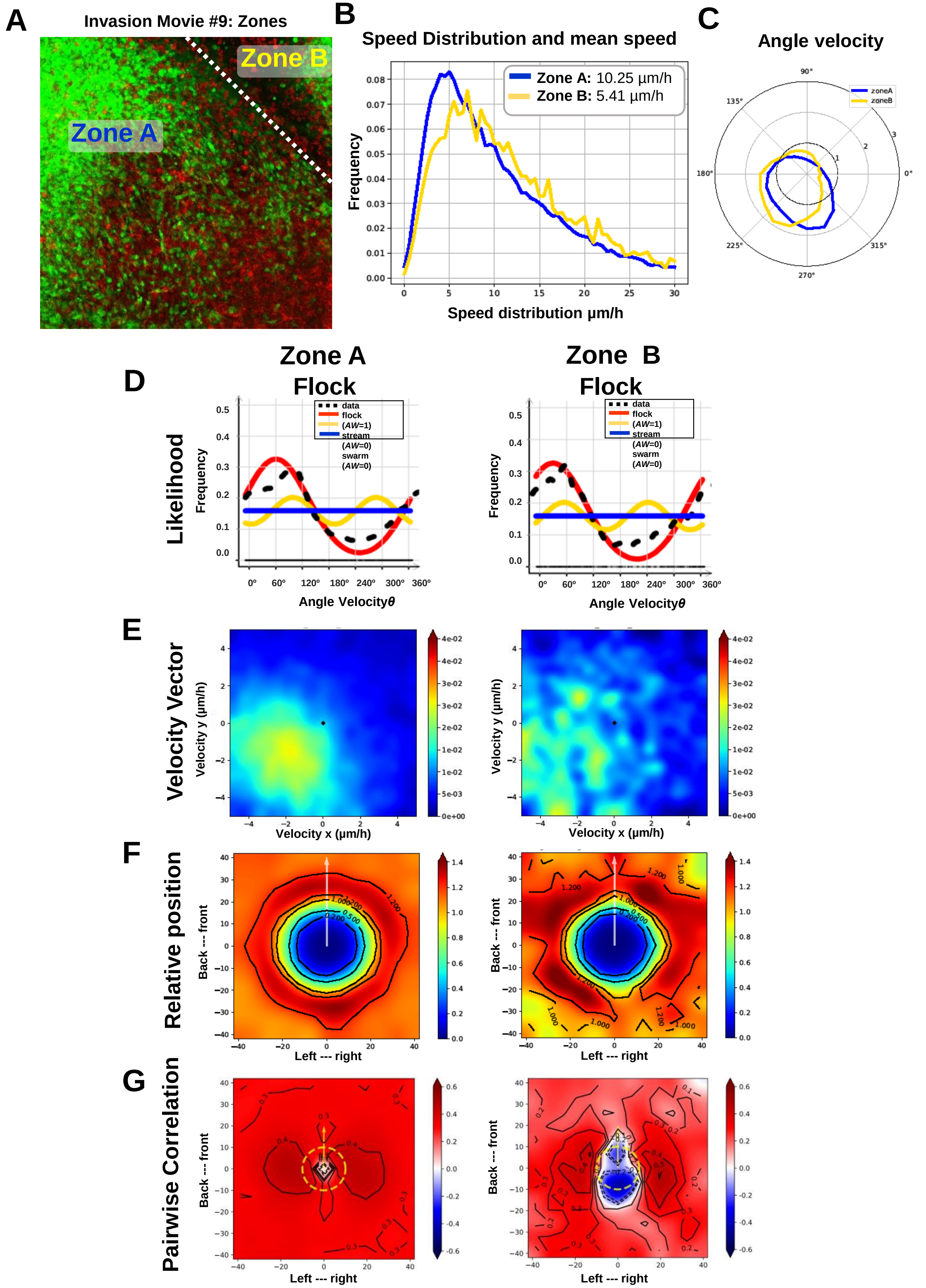
A
Invasion Movie #9: Zones
Zone A
Zone B
B
Speed Distribution and mean speed
Zone A: 10.25 µm/h
Zone B: 5.41 µm/h
Frequency
Speed distribution µm/h
C
Angle velocity
zoneA
zoneB
D
Zone A Flock
Zone B Flock
Likelihood
data
flock (AW=1)
stream (AW=0)
swarm (AW=0)
Frequency
Angle Velocityθ
E
Velocity Vector
Velocity y (µm/h)
Velocity x (µm/h)
F
Relative position
Back --- front
Left --- right
G
Pairwise Correlation
Back --- front
Left --- right

Fig. S26: Invasion analysis using confocal time-lapse imaging on the tumor border: Movie #9. A) Labeling of different regions of the tumor border for movie #8. Representative time-lapse confocal images subdivided into dynamic Zones A and B. The image shows tumor cells (green) and brain parenchyma (red). **B)** Speed distribution (*µm*/*h*) in Zone A (blue) and B (yellow). Inner panel shows mean speed for each zone. **C)** Angle Velocity distribution analysis (θ) performed by zones. **D)** Likelihood analysis histograms to classify dynamic motion patterns. Zone A: flock, Zone B: flock. AW: 0 or AW:1. **E)** Heat map plot of the distribution of the velocity vectors in each zone. **F)** Histogram plot showing the relative position with nearby neighbors for each zone. *x* and *y* axes are in µm. Bars to the right of each figure indicate probability values in color. **G)** Histograms of pairwise correlations with nearby neighbors for each zone. *x* and *y* axes are in µm. Bars to the right of each figure indicate correlation values in colors.

Fig. S27

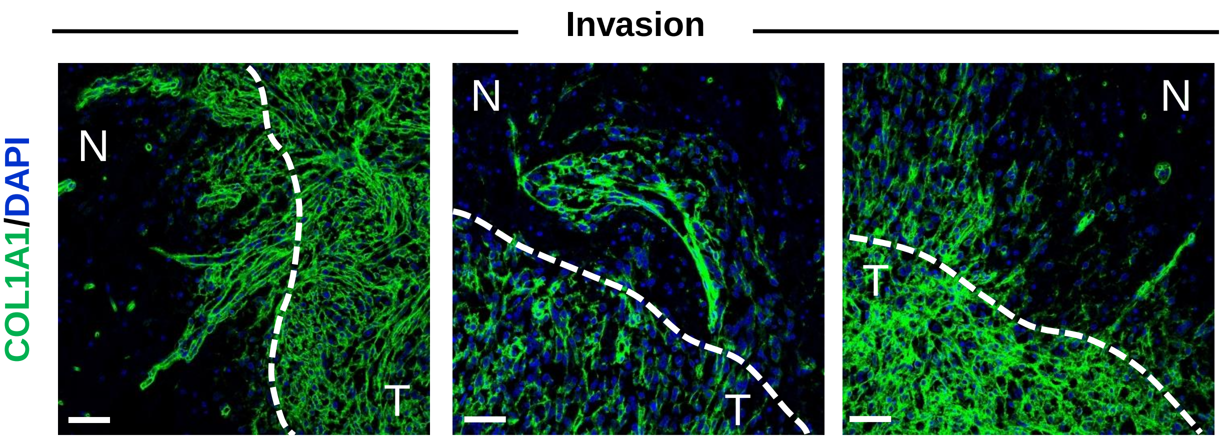

Fig. S27. Role of COL1A1 fibers in oncostreams invasion of the normal brain. Representative images of immunofluorescence analysis of COL1A1 expression in a genetically engineered mouse glioma, NPA (IDH1-WT). Images show aligned fibers of collagen1A1 along multicellular fascicles of glioma cells invading the normal brain. We propose that Collagen fibers function as scaffolds for collective tumoral cell migration. COL1A1 expression in green (Alexa 488) and nuclei in blue (DAPI). Images (20x, 1240 x 1240 pixels). White dotted line indicates tumor core border. Experiment was performed in three independent tumor sections. Scale bar: 50 µm. T=Tumor. N=normal tissue.

Fig. S28

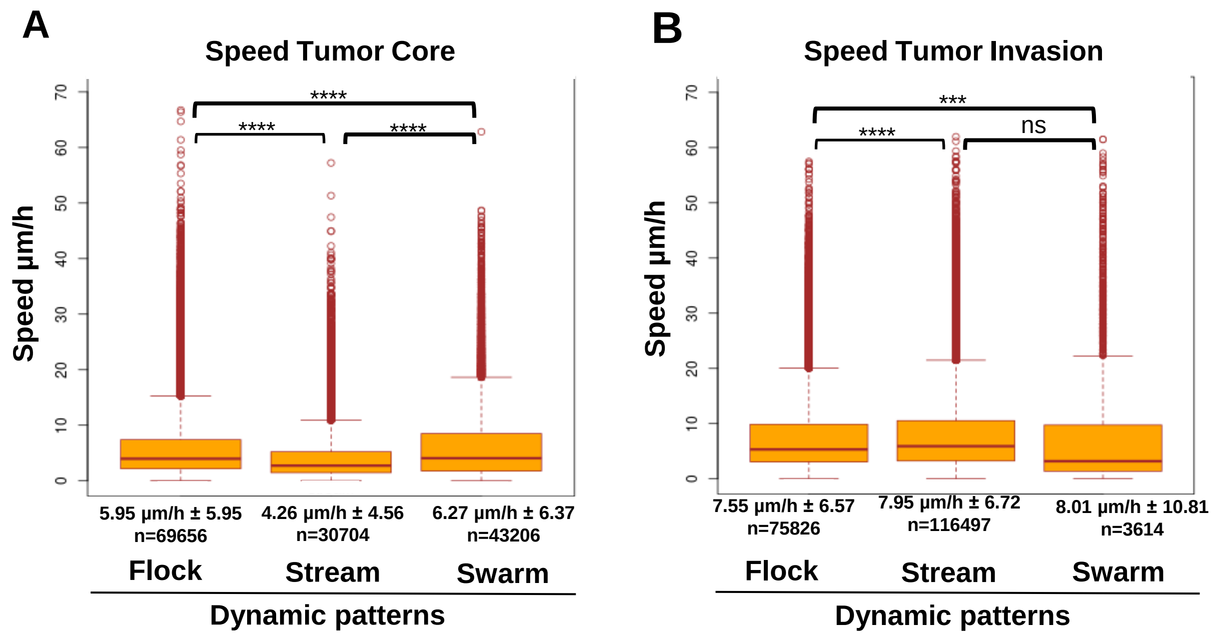

Fig. S28: Speed analysis of cells in either flocks, streams, or swarms within the tumor core or the tumor border. **A)** Box plots of cell speed (µm/hr) within flocks, streams, or swarms in the tumor core. Stream n=30704, Flock **n= 69656**, Swarm n=43206. Mean ±SEM are shown; One-way ANOVA, ****p<2.2e-16. **B)** Box plot of speed of cells (µm/hr) within flocks, streams, or swarms within the tumor border. Stream n=116497, Flock n=75826, Swarm n=3614. Mean ±SEM are shown; One-way ANOVA, flock versus swarm '***' p=0.001753, stream versus swarm 'ns' p=0.7132

Fig. S29

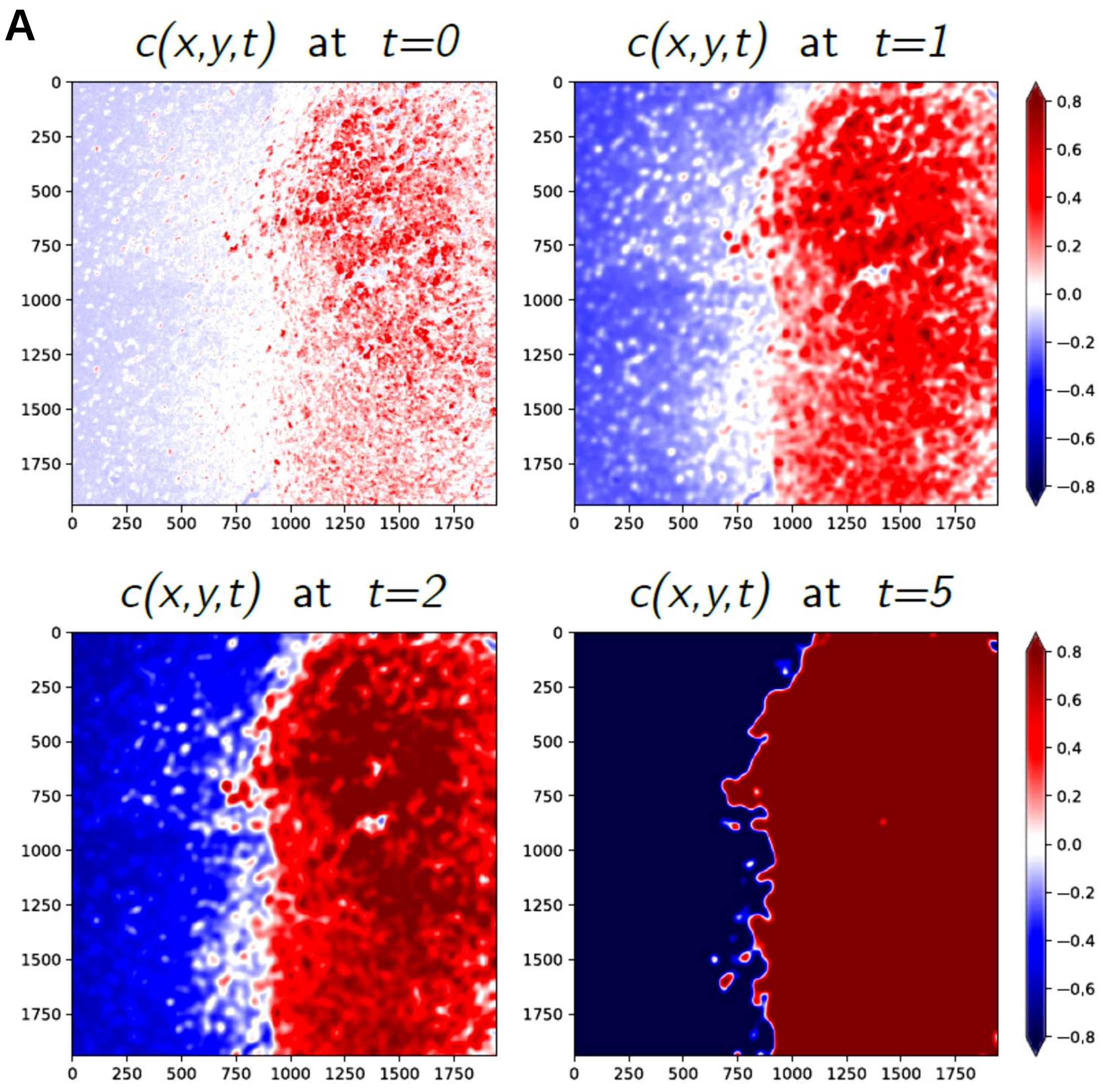

Fig. S29: Analysis of the tumor borders. A) Two-phase segmentation of an image using Allen-Cahn equation (3.1) described in Supplementary material and Methods. Each pixel has to converge to either +1 or -1 depending on its neighboring pixels. As a result, the image becomes segregated into two regions representing the inside and the outside of the tumor.

Fig. S30

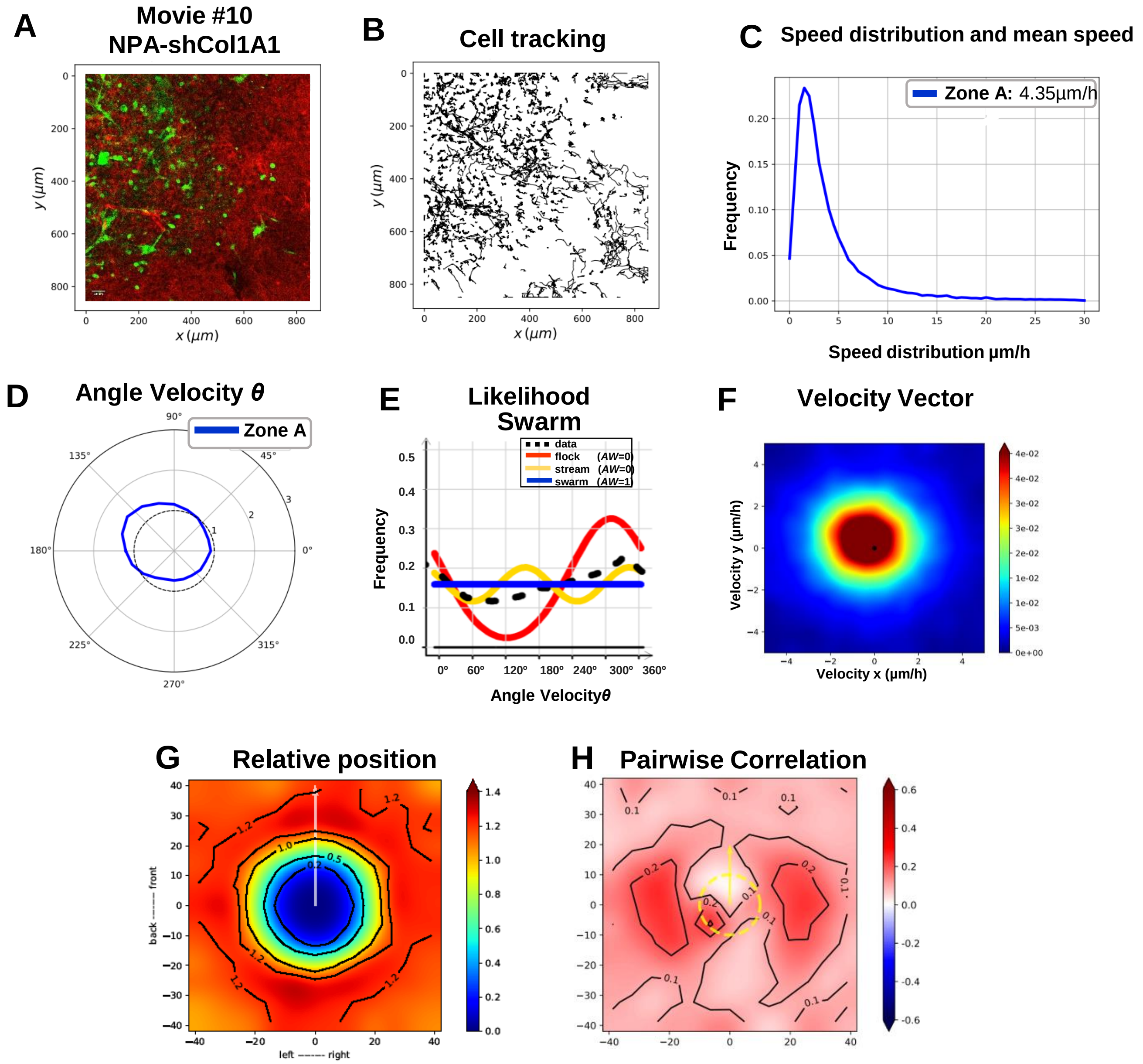

Fig. S30: Migration analysis of an NPAshCol1A1 explant model using confocal time-lapse imaging; Movie # 10. A) Representative time-lapse confocal images of the tumor border for movie #10. The image shows tumor cells (green) and brain parenchyma (red). **B)** Tracking analysis of individual cell paths performed using the Track-Mate plugin from Image-J. **C)** Speed distribution and mean speed (*µm/h*). **D)** Angle Velocity distribution analysis (*θ*) performed. **E)** Likelihood analysis histogram to classify the dynamic motion pattern as a swarm. **F)** Heat map plot of the distribution of the velocity vectors. **G)** Histogram plot showing the relative position with nearby neighbors. *x* and *y* axes are in µm. Bars to the right of each figure indicate probability values in color. **H)** Histograms of pairwise correlations with nearby neighbors. *x* and *y* axes are in µm. Bars to the right of each figure indicate correlation values in colors.

Fig. S31

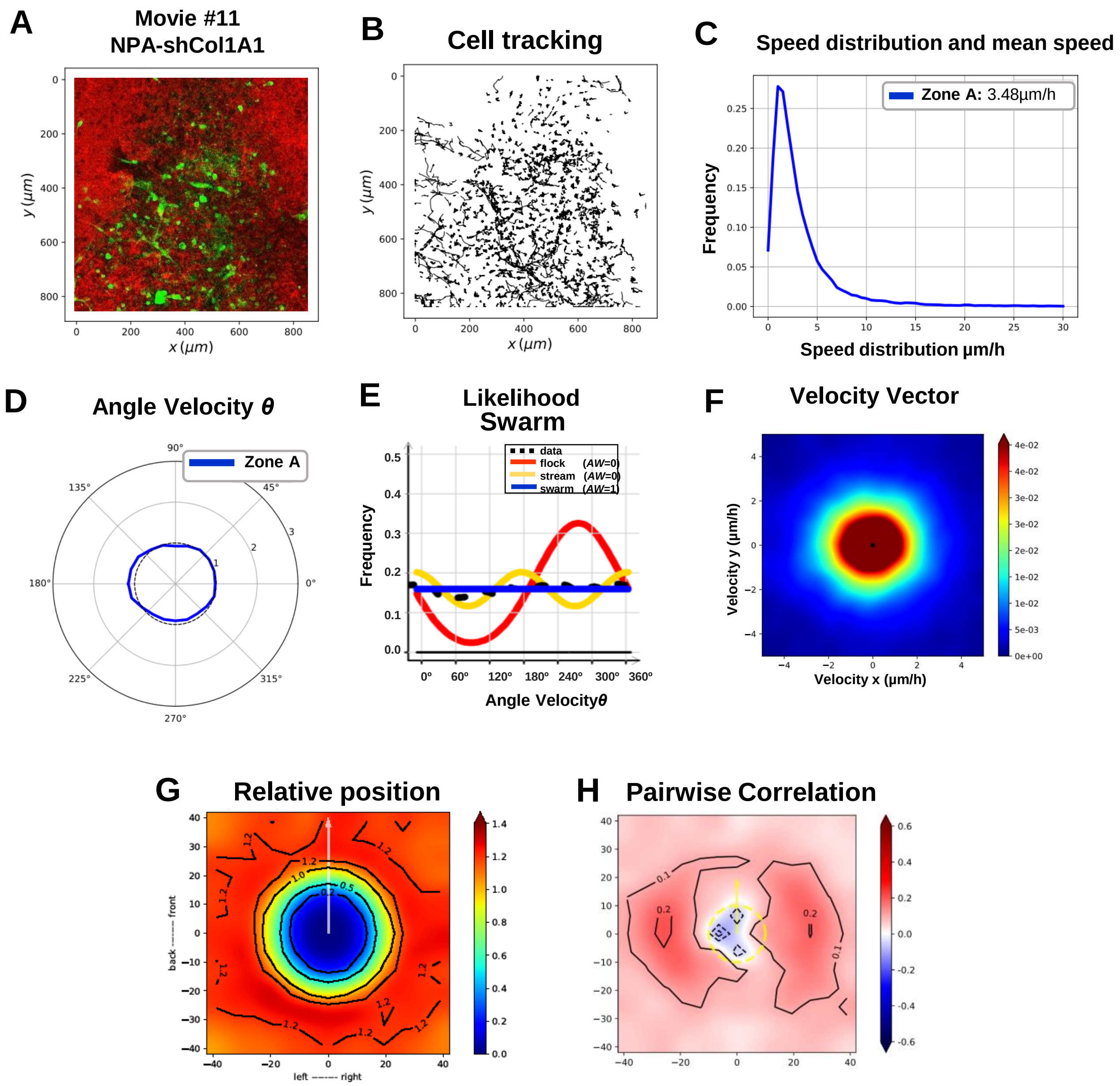

Fig. S31: Migration analysis of an NPAshCol1A1 explant model using confocal time-lapse imaging; Movie # 11. A) Representative time-lapse confocal images of the tumor border for movie #11. The image shows tumor cells (green) and brain parenchyma (red). **B)** Tracking analysis of individual cell paths performed using the Track-Mate plugin from Image-J. **C)** Speed distribution and mean speed (*µm/h*). **D)** Angle Velocity distribution analysis (*θ*) performed. **E)** Likelihood analysis histogram to classify the dynamic motion pattern as a swarm. **F)** Heat map plot of the distribution of the velocity vectors. **G)** Histogram plot showing the relative position with nearby neighbors. *x* and *y* axes are in µm. Bars to the right of each figure indicate probability values in color. **H)** Histograms of pairwise correlations with nearby neighbors, *x* and *y* axes are in µm. Bars to the right of each figure indicate correlation values in colors.

Fig. S32

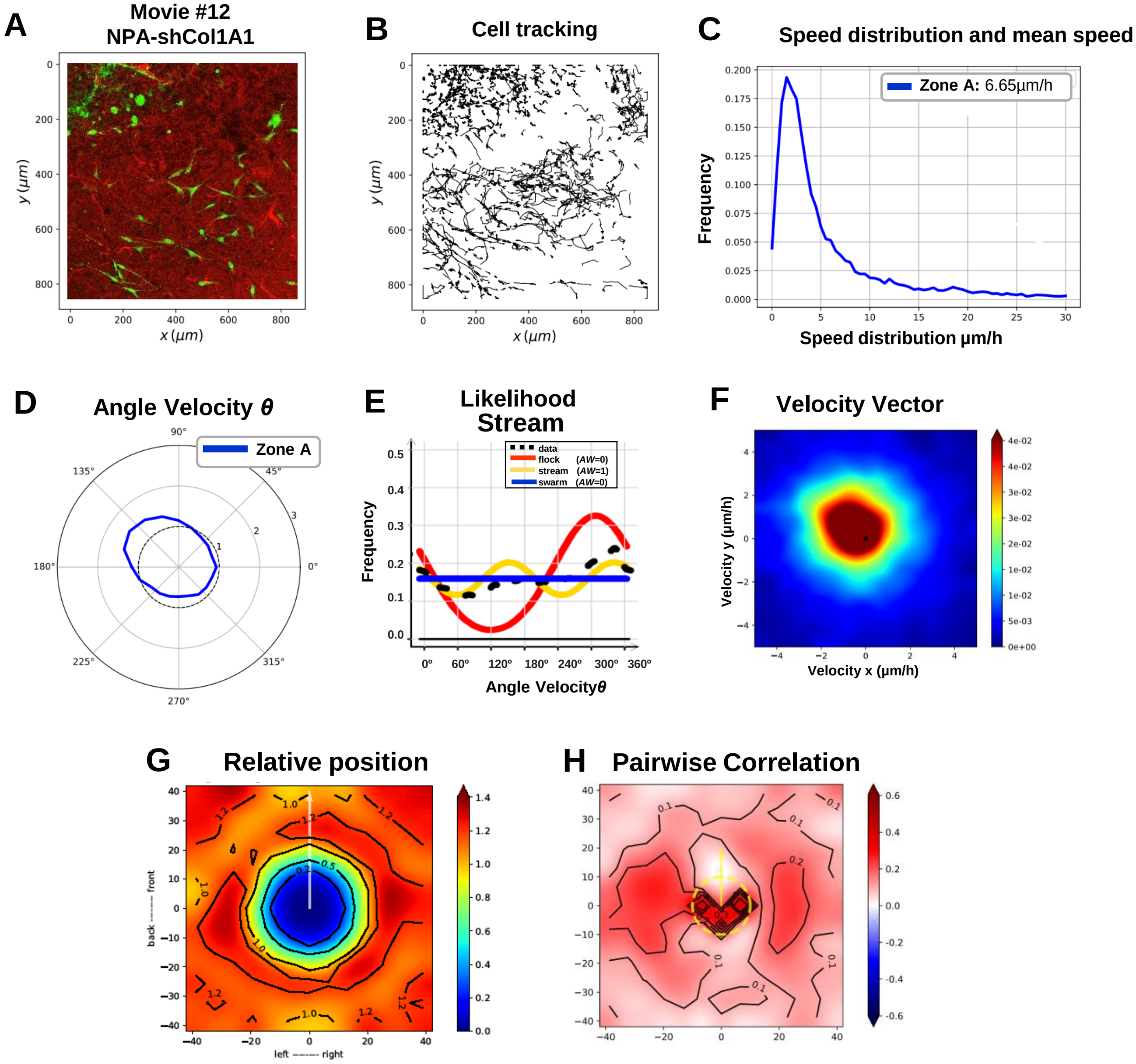

Fig. S32: Migration analysis of an NPAshCol1A1 explant model using confocal time-lapse imaging; Movie # 12. A) Representative time-lapse confocal images of the tumor border for movie #12. The image shows tumor cells (green) and brain parenchyma (red) **B)** Tracking analysis of individual cell paths performed using the Track-Mate plugin from Image-J. **C)** Speed distribution and mean speed (*μm*/*h*). **D)** Angle Velocity distribution analysis (θ) performed. **E)** Likelihood analysis histogram to classify the dynamic motion pattern as a swarm. **F)** Heat map plot of the distribution of the velocity vectors. **G)** Histogram plot showing the relative position with nearby neighbors. *x* and *y* axes are in µm. Bars to the right of each figure indicate probability values in color. **H)** Histograms of pairwise correlations with nearby neighbors. *x* and *y* axes are in µm. Bars to the right of each figure indicate correlation values in colors.

Fig. S33

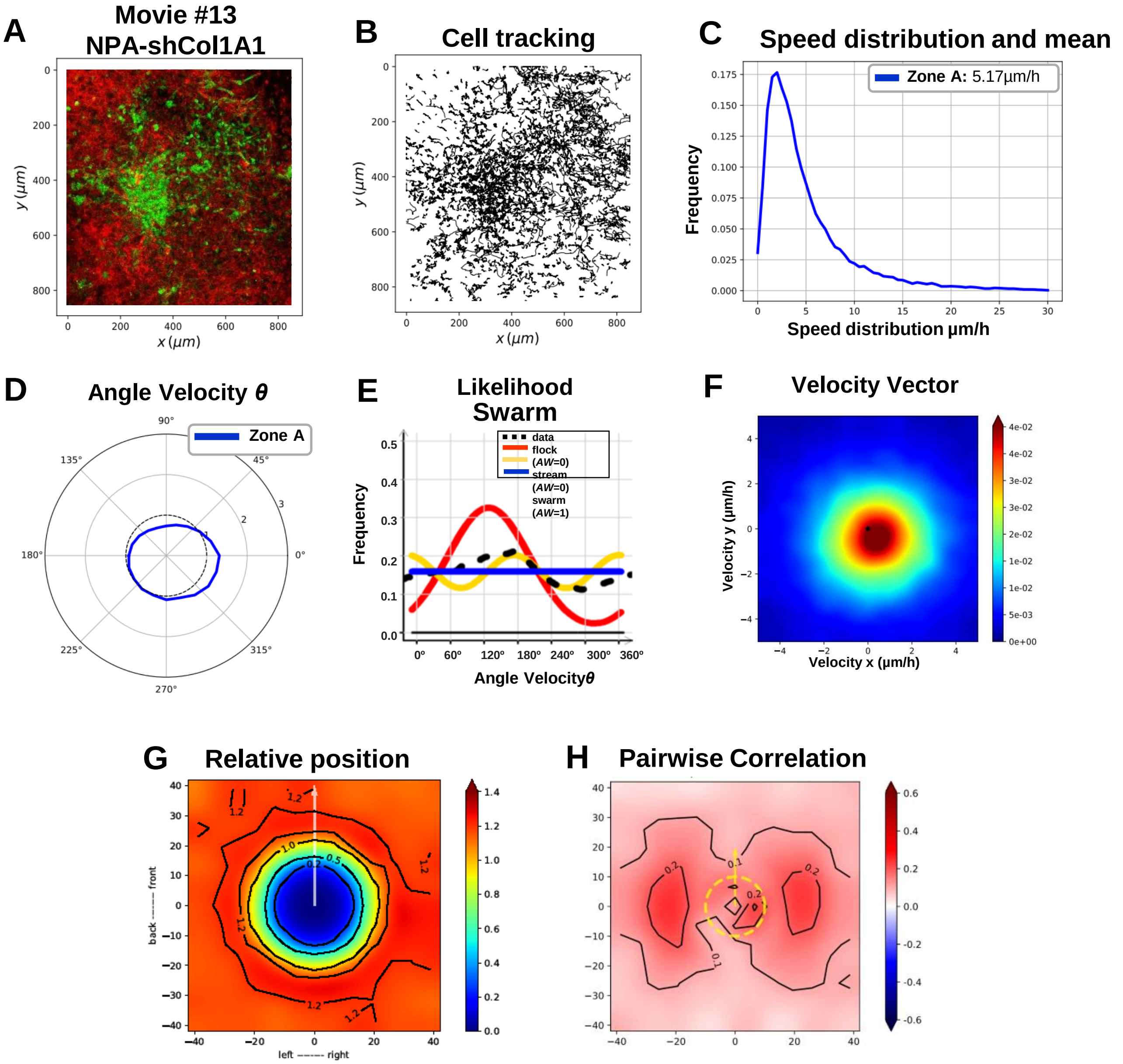

Fig. S33: Migration analysis of NPAshCol1A1 explant model using confocal time-lapse imaging; Movie # 13 A) Representative time-lapse confocal images of the tumor border for movie #13. The image shows tumor cells (green) and brain parenchyma (red). **B)** Tracking analysis of individual cell paths performed using the Track-Mate plugin from Image-J. **C)** Speed distribution and mean speed (*µm/h*). **D)** Angle Velocity distribution analysis (*θ*) performed. **E)** Likelihood analysis histogram to classify the dynamic motion pattern as a swarm. **F)** Heat map plot of the distribution of the velocity vectors. **G)** Histogram plot showing the relative position with nearby neighbors. *x* and *y* axes are in µm. Bars to the right of each figure indicate probability values in color. **H)** Histograms of pairwise correlations with nearby neighbors. *x* and *y* axes are in µm. Bars to the right of each figure indicate correlation values in colors.

Fig. S34

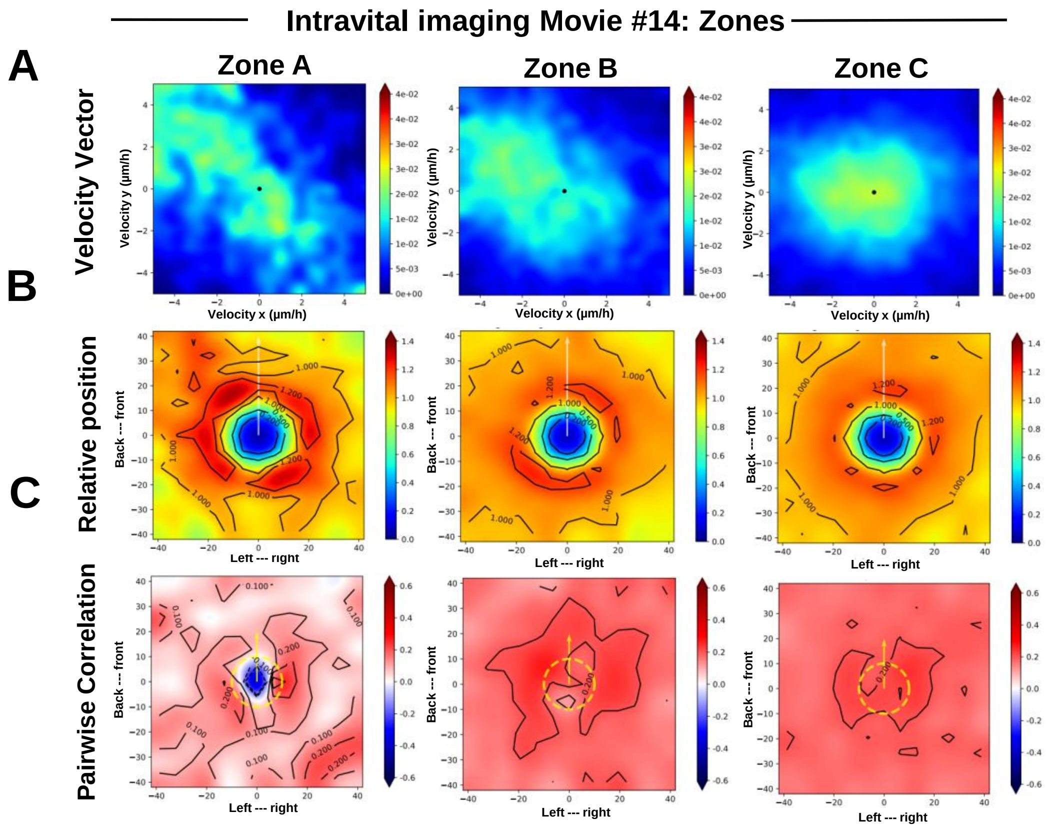

Fig. S34. Migration analysis in intravital imaging Movie#14

A) Heat map plot of the distribution of the velocity vectors in each zone. **B)** Histogram plot showing the relative position with nearby neighbors for each zone. x and y axes are in μm. Bars to the right of each figure indicate probability values in color. **C)** Histograms of pairwise correlations with nearby neighbors for each zone. x and y axes are in μm. Bars to the right of each figure indicate correlation values in colors.

Fig. S35

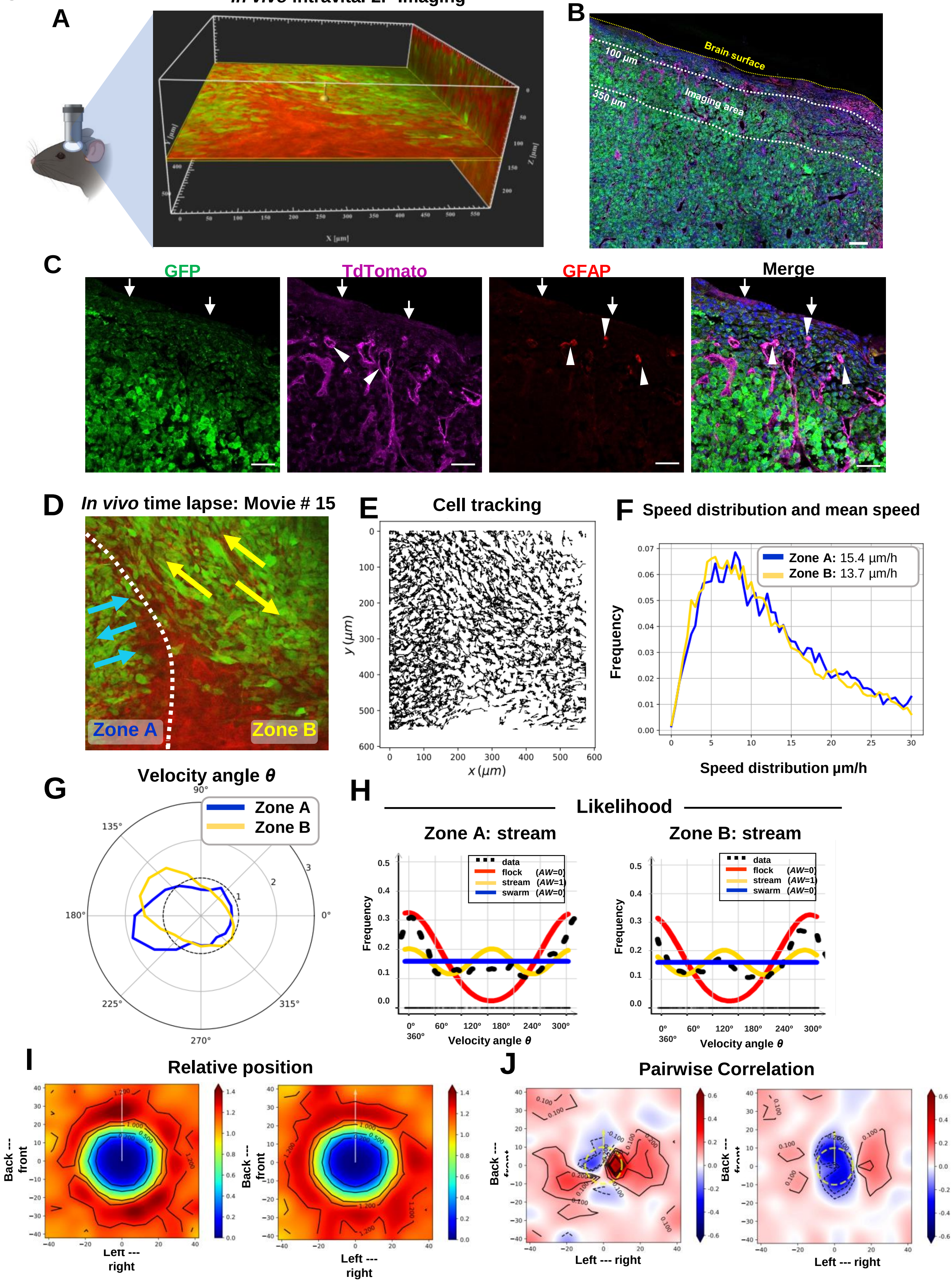
A
In vivo intravital 2P Imaging
B
Brain surface
100 µm
Imaging area
350 µm
C
GFP
TdTomato
GFAP
Merge
D
In vivo time lapse: Movie # 15
Zone A
Zone B
E
Cell tracking
F
Speed distribution and mean speed
Zone A: 15.4 µm/h
Zone B: 13.7 µm/h
Frequency
Speed distribution µm/h
G
Velocity angle θ
Zone A
Zone B
H
Likelihood
Zone A: stream
Zone B: stream
data
flock (AW=0)
stream (AW=1)
swarm (AW=0)
Frequency
Velocity angle θ
I
Relative position
Back --- front
Left --- right
J
Pairwise Correlation
Back --- front
Left --- right

Fig. S35. Intravital two-photon imaging setup for time-lapse acquisition and immunofluorescence of coronal section of the imaging area: Movie #15

A) shows an image generated using the Orthoslicer 3D function of the Imaris viewer software illustrating the plane used for time-lapse imaging. The image depicts the imaging plane and its distance from the brain's surface. For this experiment, we took 248 x-y frames from the brain's surface at a depth increment of 1 µm (voxel size=1) and resolution of 1024x1024 pixels. XYZ axes of the orthoslicer3D image are shown in white color (596x596x248 µm). The imaging plane was at 140 µm from the brain surface. Normal brain parenchyma Is shown in red and tumor cells in green. (Created in BioRender.com) **B-C)** Following *in vivo* intravital imaging, the animal was perfused, and the brain was fixed and embedded in paraffin. Immunofluorescence was performed on 5 µm thick sections. This experiment was repeated three times. The image shows tumor cells (green) and brain parenchyma (red). **B)** The brain surface is marked in yellow, and the imaging area is marked in between white lines (100 to 350 µm in depth). Sections were stained with anti-GFP (green) to detect tumor cells, anti-tdTomato (pink) to detect brain parenchyma cells, and anti-GFAP (red) to detect astrocytes. Scale bar: 100 µm. **C)** From left to right: Tumor cells (GFP+) growing within the cortex. The brain parenchymal cells (anti-tdTomato+) are present in the brain surface, blood vessels, as well as in between tumor cells. GFAP+ cell processes can be found within the tumor. The merged channel shows the tumor growing mainly below the brain surface and within the brain parenchyma. The tumor cells are interacting with the brain parenchyma (blood vessels, astrocytes). Scale bar: 50 µm. **D)** Single representative time-lapse two-photon image of glioma cell invasion *in vivo* (Movie #15), showing Zones A, B. The image shows tumor cells (green) and brain parenchyma (red). **E)** Individual cell paths trajectories of this *in vivo* time-lapse experiment. **F)** Histogram of speed distribution and mean speed (µm/h) in Zones A, B. **G)** Angle Velocity distribution for each zone's in the *in vivo* time-lapse. The Angle Velocity of each cell is denoted θ. The plot shows the proportion of cells moving in the angle direction θ for each zone. **H)** Likelihood analysis of the dynamic patterns in each zone of Movie #15 by intravital imaging. Graph of frequency distribution *ρ flock* (red), *ρ stream* (yellow) and *ρ swarm* (blue). The estimation of the black line (data) uses a *non-parametric* estimation. AW: 0 or AW:1. **I)** Histogram plot showing the relative position with nearby neighbors for each zone; *x* and *y* axes are in µm. Bars to the right of each figure indicate probability values in color. **J)** Histograms of pairwise correlations with nearby neighbors for each zone; *x* and *y* axes are in µm. Bars to the right of each figure indicate correlation values in colors.

Fig. S36

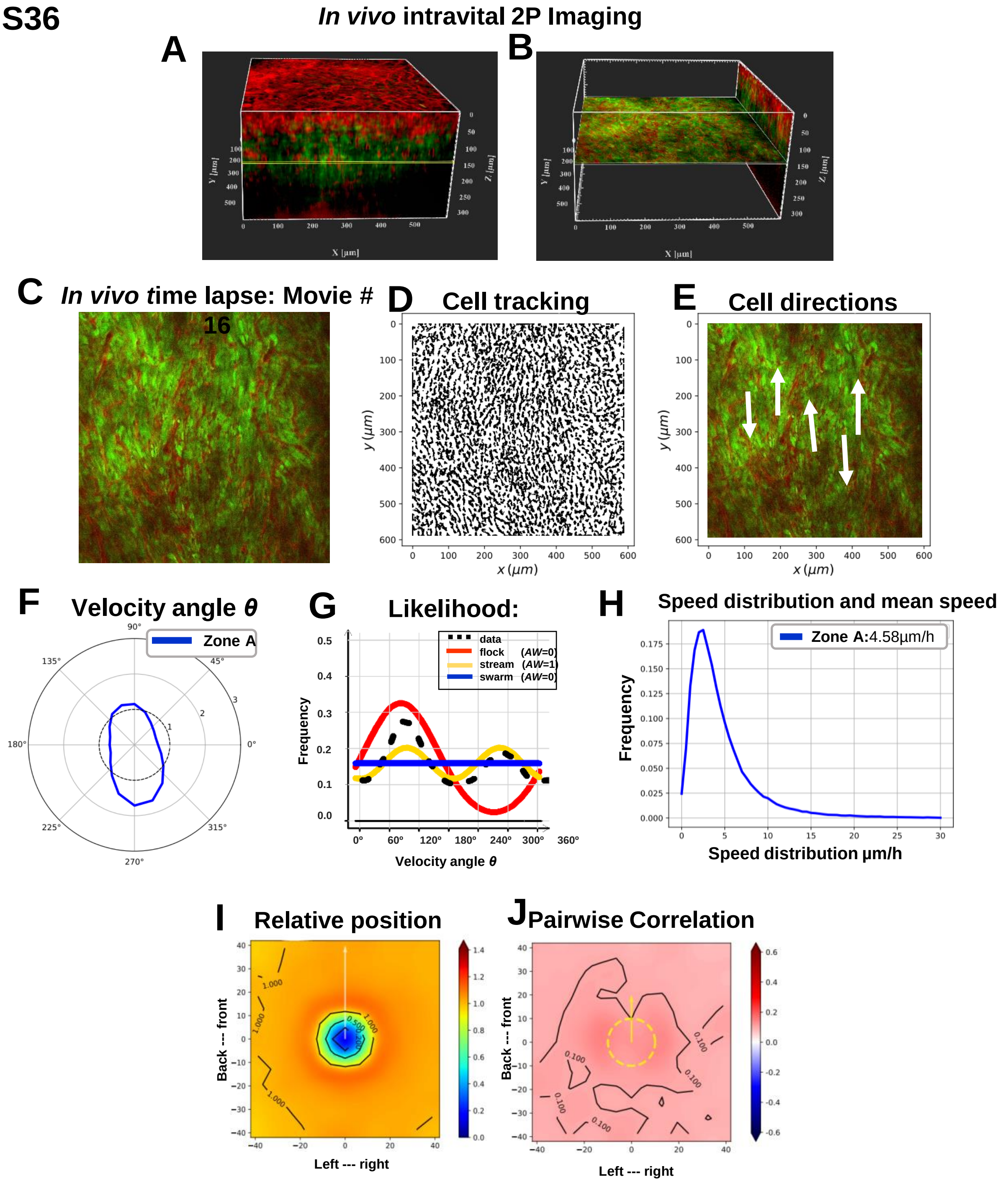

In vivo intravital 2P Imaging
A
B
X [µm]
Y [µm]
Z [µm]
C In vivo time lapse: Movie # 16
D Cell tracking
E Cell directions
y (µm)
x (µm)
F Velocity angle θ
Zone A
G Likelihood:
data
flock (AW=0)
stream (AW=1)
swarm (AW=0)
Frequency
Velocity angle θ
H Speed distribution and mean speed
Zone A:4.58µm/h
Frequency
Speed distribution µm/h
I Relative position
Back --- front
Left --- right
J Pairwise Correlation
Back --- front
Left --- right

Fig. S36. Invasion analysis of NPA glioma dynamics within the tumor core using Intravital two-photon microscopy *in vivo:* Movie #16

A) High-resolution 3D Z-stacks (spanning up to 330 µm depth from the brain's surface) were imported into the Imaris viewer to reconstruct the 3D image. To do so, 330 x-y frames from the brain's surface were taken at a depth increment of 1 µm (voxel size=1) and resolution of 1024x1024 pixels. The XYZ axes of the 3D image are shown in white color (each comprising an area of 596x596x330 µm), and the yellow line (at 145 µm depth) shows the exact imaging plane used to obtain the *in vivo* time-lapse data for movie #16. The image shows tumor cells (green) and brain parenchyma (red). **B)** shows an image generated with the Orthoslicer3D from Imaris using the 3D reconstruction data shown in **(A)**; this illustrates the depth of the imaging plane and its distance from the brain surface. Normal brain parenchyma is shown in red, and tumor cells are shown in green. **C)** Single representative time-lapse two-photon image of glioma cells within the tumor core *in vivo* taken at a depth of 145 µm. **D)** Individual cell paths trajectories of this *in vivo* time-lapse experiment. **E)** Preferred directions of cells within the tumor core superimposed onto a representative time-lapse image. **F)** Angle Velocity distribution for the migration of cells growing within the tumor core for the *in vivo* time-lapse dataset. Angle Velocity for each cell is denoted θ. The plot shows the proportion of cells moving in the angle direction θ. **G)** The frequency distribution corresponding to Angle Velocity(θ) is shown by dotted black lines. Likelihood analysis of the dynamic patterns within the tumor core (Movie#16). Graph of frequency distribution is shown as *ρ flock* (red), *ρ stream* (yellow) and *ρ swarm* (blue). The estimation of the black line (data) uses *non-parametric* estimation. AW: 0 or AW:1. **H)** Histogram of speed distribution and mean speed in µm/hr for the full time-lapse movie considered as a single zone named Zone A. **I)** Histogram plot showing the relative position of nearest neighbors for Zone A; *x* and *y* axes are in µm. Bars to the right of each figure indicate probability values in color. **J)** Histograms of pairwise correlations with nearby neighbors for Zone A; *x* and *y* axes are in µm. Bars to the right of each figure indicate correlation values in colors.

Fig. S37

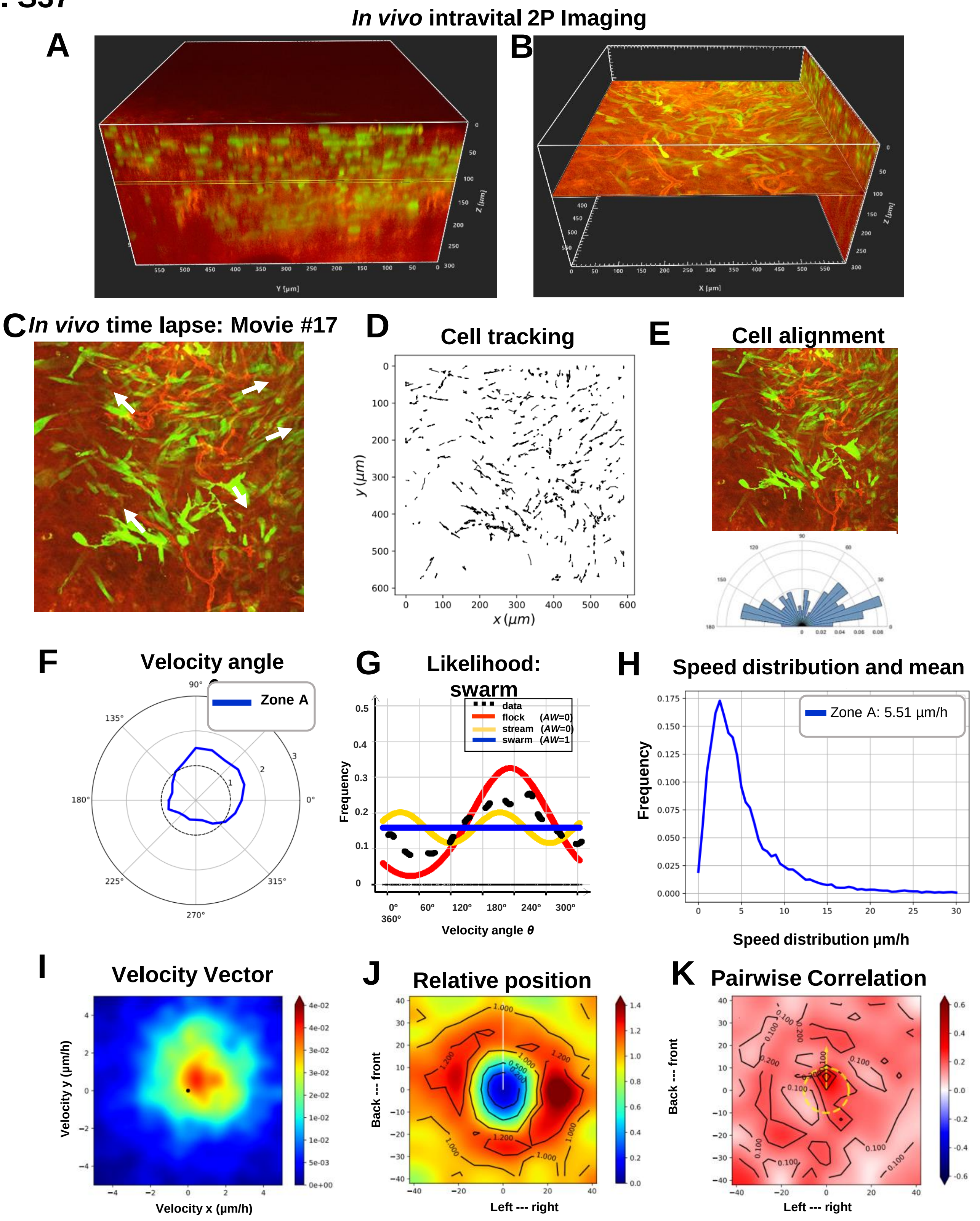
In vivo intravital 2P Imaging
A
B
Y [µm]
Z [µm]
X [µm]
C In vivo time lapse: Movie #17
D Cell tracking
y (µm)
x (µm)
E Cell alignment
F Velocity angle
Zone A
G Likelihood: swarm
data
flock (AW=0)
stream (AW=0)
swarm (AW=1
Frequency
Velocity angle θ
H Speed distribution and mean
Zone A: 5.51 µm/h
Frequency
Speed distribution µm/h
I Velocity Vector
Velocity y (µm/h)
Velocity x (µm/h)
J Relative position
Back --- front
Left --- right
K Pairwise Correlation
Back --- front
Left --- right

Fig. S37. Invasion analysis of NPAshCol1A1 glioma dynamics using Intravital two-photon microscopy *in vivo:* Movie #17

A) High-resolution 3D Z-stacks (spanning up to 300 µm depth from the brain's surface) were imported into the Imaris viewer to reconstruct the 3D image. To do so, 300 x-y frames from the brain's surface were taken at a depth increment of 1 µm (voxel size=1) and resolution of 1024x1024 pixels. The XYZ axes of the 3D image are shown in white color (each comprising an area of 596x596x300 µm), and the yellow line (at 110 µm depth) shows the exact imaging plane used to obtain the *in vivo* time-lapse data for movie #17. The image shows tumor cells (green) and brain parenchyma (red). **B)** shows an image generated with the Orthoslicer3D from Imaris using the 3D reconstruction data shown in **(A)**; this illustrates the depth of the imaging plane and its distance from the brain surface. Normal brain parenchyma is shown in red, and tumor cells are shown in green. **C)** Single representative time-lapse two-photon image of NPAshCol1A1 glioma cells within the tumor core *in vivo* taken at a depth of 110 µm. Preferred directions of cells within tumor core superimposed onto a representative time-lapse image. **D)** Individual cell paths trajectories of this *in vivo* time-lapse experiment. **E)** Angle histogram plots show the random alignment of NPAshCol1A1 glioma cells lacking oncostreams *in vivo*. Angle histograms correspond to the representative time-lapse two-photon image on the top. **F)** Angle Velocity distribution for the migration of NPAshCol1A1 glioma cells growing within the tumor core for the *in vivo* time-lapse dataset. The Angle Velocity of each cell is denoted θ. The plot shows the proportion of cells moving in the angle direction θ. **G)** The frequency distribution corresponding to Angle Velocity(θ) is shown by dotted black lines. Likelihood analysis of the dynamic patterns within the tumor core (Movie#17). Graph of frequency distribution is shown as *ρ flock* (red), *ρ stream* (yellow) and *ρ swarm* (blue). The estimation of the black line (data) uses a *non-parametric* estimation. AW:0 or AW:1. **H)** Histogram of speed distribution and mean speed in µm/h for the full time-lapse movie considered as a single zone named Zone A. **I)** Heatmap plot of the distribution of velocity vectors within Zone A. **J)** Histogram plot showing the relative position of nearest neighbors for Zone A; *x* and *y* axes are in µm. Bars to the right of each figure indicate probability values in color. **K)** Histograms of pairwise correlations with nearby neighbors for Zone A; *x* and *y* axes are in µm. Bars to the right of each figure indicate correlation values in colors.
