# Supplementary material for: Spatiotemporal analysis of glioma heterogeneity reveals COL1A1 as an actionable target to disrupt tumor progression
Source: Nat Commun. 2022 Jun 24;13:3606. doi: 10.1038/s41467-022-31340-1 (PMC9232499; doi:10.1038/s41467-022-31340-1)
Supplement: Supplementary file 3 — Description to Additional Supplementary Information [file 41467_2022_31340_MOESM3_ESM.docx]

**Inventory of Supporting Information**

**Supplementary Data 1. Oncostreams analysis using TCGA-GBM and TCGA-LGG histological tissues database**

**Supplementary Data 2. Oncostreams’ differentially gene expression analysis**

**Movies #1 Glioma dynamics at the tumor core**

**Movies #2 Glioma dynamics at the tumor core**

**Movies #3 Glioma dynamics at the tumor core**

**Movies #4 Glioma dynamics at the tumor core**

**Movies #5 Glioma dynamics at the tumor border**

**Movies #6 Glioma dynamics at the tumor border**

**Movies #7 Glioma dynamics at the tumor border**

**Movies #8 Glioma dynamics at the tumor border**

**Movies #9 Glioma dynamics at the tumor border**

**Movies #10 Glioma dynamics of NPAshCOL1A1 tumors**

**Movies #11 Glioma dynamics of NPAshCOL1A1 tumors**

**Movies #12 Glioma dynamics of NPAshCOL1A1 tumors**

**Movies #13 Glioma dynamics of NPAshCOL1A1 tumors**

**Movies #14  *In vivo* NPA glioma dynamics patterns employing 2-Photon intravital imaging**

**Movies #15  *In vivo* NPA glioma dynamics patterns employing 2-Photon intravital imaging**

**Movies #16  *In vivo* NPA glioma dynamics patterns employing 2-Photon intravital imaging**

**Movie 17. *In vivo* NPAshCOL1A1 glioma dynamics patterns employing 2-Photon intravital imaging**
