# Supplementary material for: Spatiotemporal analysis of glioma heterogeneity reveals COL1A1 as an actionable target to disrupt tumor progression
Source: Nat Commun. 2022 Jun 24;13:3606. doi: 10.1038/s41467-022-31340-1 (PMC9232499; doi:10.1038/s41467-022-31340-1)
Supplement: Supplementary file 23 — Reporting Summary [file 41467_2022_31340_MOESM23_ESM.pdf]

nature portfolio

Corresponding author(s): Pedro R Lowenstein

Last updated by author(s): June 06th, 2022

Reporting Summary

Nature Portfolio wishes to improve the reproducibility of the work that we publish. This form provides structure for consistency and transparency in reporting. For further information on Nature Portfolio policies, see our Editorial Policies and the Editorial Policy Checklist.

Statistics

For all statistical analyses, confirm that the following items are present in the figure legend, table legend, main text, or Methods section.

n/a	Confirmed	
☐	☒	The exact sample size (*n*) for each experimental group/condition, given as a discrete number and unit of measurement
☐	☒	A statement on whether measurements were taken from distinct samples or whether the same sample was measured repeatedly
☐	☒	The statistical test(s) used AND whether they are one- or two-sided<br>*Only common tests should be described solely by name; describe more complex techniques in the Methods section.*
☒	☐	A description of all covariates tested
☐	☒	A description of any assumptions or corrections, such as tests of normality and adjustment for multiple comparisons
☐	☒	A full description of the statistical parameters including central tendency (e.g. means) or other basic estimates (e.g. regression coefficient) AND variation (e.g. standard deviation) or associated estimates of uncertainty (e.g. confidence intervals)
☐	☒	For null hypothesis testing, the test statistic (e.g. F, t, r) with confidence intervals, effect sizes, degrees of freedom and P value noted<br>*Give P values as exact values whenever suitable.*
☒	☐	For Bayesian analysis, information on the choice of priors and Markov chain Monte Carlo settings
☒	☐	For hierarchical and complex designs, identification of the appropriate level for tests and full reporting of outcomes
☒	☐	Estimates of effect sizes (e.g. Cohen's d, Pearson's r), indicating how they were calculated

Our web collection on statistics for biologists contains articles on many of the points above.

Software and code

Policy information about availability of computer code

Data collection	Olympus BX53 Upright Microscope,<br>Laser Scanning Confocal Microscope (LSM 880, Axio Observer, Zeiss, Germany),<br>Leica Laser Capture Microdissection (LMD7000),<br>Illumina HiSeq 4000,<br>Leica ASP 300 paraffin tissue processor/Tissue-Tek paraffin tissue embedding station (Leica, Buffalo Grove IL), and<br>Bruker Optima two-photon microscope, using a Chameleon Ti-sapphire laser (Coherent).

Data analysis	ImageJ version 1.53f51,<br>Zen (Blue edition) version 2.5<br>Fiji using Java8,<br>GraphPad Prism version 8.0,<br>Statistical Analysis System (SAS), 2021 SAS Institute (Cary, NC),<br>Julia version 1.0.5,<br>Atom version 1.58.0,<br>RStudio version 1.4,<br>MATLAB version R2019b update 3 (9.7.0.1261785),<br>Imaris viewer version 9.8 (Bitplane, Imaris, Oxford Instruments, MA, USA),<br>Cytoscape version 3.7.1 (https://cytoscape.org),<br>Custom codes to analyze oncostreams using U-Net architecture and deep learning can be found at https://github.com/MLNeurosurg/DeepStreams,<br>Custom codes to analyze glioma dynamics using the Julia Programing Language can be found at public GitHub repository https://github.com/smotsch/analysis_glioma.

For manuscripts utilizing custom algorithms or software that are central to the research but not yet described in published literature, software must be made available to editors and reviewers. We strongly encourage code deposition in a community repository (e.g. GitHub). See the Nature Portfolio guidelines for submitting code & software for further information.

Data

Policy information about availability of data

All manuscripts must include a data availability statement. This statement should provide the following information, where applicable:

- Accession codes, unique identifiers, or web links for publicly available datasets
- A description of any restrictions on data availability
- For clinical datasets or third party data, please ensure that the statement adheres to our policy

Data availability: All data associated with this study are in the paper and/or the Supplementary Information. RNA-Seq dataset generated in this study have been deposited at the NCBI's Gene Expression Omnibus (GEO) with identifier GSE188970 [https://www.ncbi.nlm.nih.gov/geo/query/acc.cgi?acc=GSE188970]. Additionally, the following public databases were used: TCGA glioma diagnostic tissue slides from the Genomic Data Commons Portal of the National Cancer Institute [https://portal.gdc.cancer.gov]. Clinical data [http://firebrowse.org]; [http://gliovis.bioinfo.cnio.es]; and [https://portal.gdc.cancer.gov]. TCGA dataset related to Col1A1, E-Cadherin, and N-Cadherin expression and its correlation with patient survival [http://gliovis.bioinfo.cnio.es]. To select shRNA targeting Col1a1 gene, we used the RNAi codex database [http://cancan.cshl.edu/cgi-bin/Codex/Codex.cgi] and InvivoGen's siRNA Wizard [http://www.invivogen.com/sirnawizard]. All the movies/imaging data generated in this study have been provided in the supplementary information. The remaining data are available within the article, supplementary information, or source data file. Cells, plasmids, and other reagents developed in this study could be made available upon request to pedrol@umich.edu. Source data are provided with this paper.

Code Availability: The analysis of oncostreams in mouse and human glioma tissue was performed using U-Net architecture to provide semantic segmentation of specimens using deep learning. Public GitHub repository for the project code can be found at https://github.com/MLNeurosurg/DeepStreams.
Analysis of glioma cells dynamics was performed using the Julia Programing Language. Link for this project Script and their dependencies can be found at public GitHub repository https://github.com/smotsch/analysis_glioma.

Field-specific reporting

Please select the one below that is the best fit for your research. If you are not sure, read the appropriate sections before making your selection.

☒ Life sciences ☐ Behavioural & social sciences ☐ Ecological, evolutionary & environmental sciences

For a reference copy of the document with all sections, see nature.com/documents/nr-reporting-summary-flat.pdf

Life sciences study design

All studies must disclose on these points even when the disclosure is negative.

Sample size	Following standards of the field, including power analysis, sample sizes were estimated which were capable of yielding statistically significant differences. For in vivo studies, at least $n \geq 3$ mice were utilized. To verify the data obtained, and estimate the median survival at least $n \geq 3$ biological replicates were chosen on the basis of previously published studies (PMID: 26936505, PMID: 30760578, and PMID: 34586841). The number of replicates used are mentioned in the corresponding figure legends.<br>For sleeping beauty survival experiments, at least $n \geq 9$ were used, to detect a hazard ratio of 0.714 using log-rank test corresponding to at least 40% increase in median survival/improvement. In cases where sample size was less than three due to experimental limitations, statistical differences were not presented.
Data exclusions	No data were excluded from the analyses.
Replication	Replicates were used in all experiments as noted in the manuscript and figure legends. All experiments were repeated at least three times with reproducible results.
Randomization	All samples such as cells or animals were randomly allocated into experimental groups.

Blinding	We were not blinded to group allocations during data collection and analysis of ex vivo and in vivo experiments involving various experimental groups. In order to perform the imaging and dynamic analysis ex-vivo and in-vivo, it was imperative to know the assigned experimental group. In case of image analysis, experimental images were blinded during selection of the imaging.

Reporting for specific materials, systems and methods

We require information from authors about some types of materials, experimental systems and methods used in many studies. Here, indicate whether each material, system or method listed is relevant to your study. If you are not sure if a list item applies to your research, read the appropriate section before selecting a response.

Materials & experimental systems

n/a	Involved in the study
☐	☒ Antibodies
☐	☒ Eukaryotic cell lines
☒	☐ Palaeontology and archaeology
☐	☒ Animals and other organisms
☐	☒ Human research participants
☒	☐ Clinical data
☒	☐ Dual use research of concern

Methods

n/a	Involved in the study
☒	☐ ChIP-seq
☒	☐ Flow cytometry
☒	☐ MRI-based neuroimaging

Antibodies

Antibodies used

Primary antibodies:
Anti-Nuclei Antibody, Clone 235-1 (HuNu), Millipore Sigma (MAB1281),
Anti-Green Fluorescent Protein (GFP), Rockland (600-101-215),
Anti-Alpha Smooth Muscle Actin (α-SMA/ACTA2), Abcam (ab5694),
Anti-Glial Fibrillary Acidic Protein (GFAP), Millipore Sigma (AB5804),
Anti-Neurofilament-L (C28e10), Cell Signaling (2837),
Anti-E-Cadherin (24E10), Cell Signaling (3195),
Anti-N-Cadherin, Abcam (AB18203),
Anti-Nestin, Novus (NB100-1604),
Anti-Sox2, Invitrogen (MA1-014),
Anti-Iba1 Antibody [EPR16588], Abcam (ab178846),
Anti-Brdu (Bu20a), Cell Signaling (5292),
Anti-Collagen I Antibody, Abcam (ab34710),
Anti-Col1A1 Polyclonal Antibody, ThermoFischer Scientific (PA5-29569)
Anti-Fibronectin, Abcam (ab2413),
Anti-CD68, Abcam (ab125212),
Anti-CD31, Cell Signaling (77699),
Anti-Survivin, Cell Signaling (2808),
Anti-PCNA, Cell Signaling (2586),
Anti-Cleaved Caspase 3, Cell Signaling (9661)
Anti-TdTomato, SICGEN (AB8181-200)
Anti-Glial Fibrillary Acidic Protein (GFAP), AMD Millipore (AB5541)
Anti-Green Fluorescent Protein (GFP), Abcam (AB290)

Secondary antibodies:

Polyclonal Goat Anti-Rabbit Immunoglobulins/Biotin, Agilent (E043201-6)
Polyclonal Goat Anti-Mouse Immunoglobulins/Biotinylated, Agilent (E0433)
Goat Anti-Chicken IgY Antibody (H+L), Biotinylated, Vector Laboratories (BA-9010-1.5)
Goat anti-Chicken IgY (H+L) Secondary Antibody, Alexa Fluor 555, Invitrogen (A-21437)
Donkey anti-Rabbit IgG (H+L), Alexa Fluor Plus 488, Invitrogen (A32790)
Donkey anti-Goat IgG (H+L), Alexa Fluor Plus 488, Invitrogen (A32814)
Donkey anti-Rabbit IgG (H+L), Alexa Fluor Plus 555, Invitrogen (A32794)
Donkey anti-Goat IgG (H+L), Alexa Fluor Plus 647, Invitrogen (A21447)
Donkey anti-Chicken IgY (IgG) (H+L), Alexa Fluor 594, Jackson ImmunoResearch Labs (703-585-155)
Donkey anti-Mouse IgG (H+L), Alexa Fluor Plus 488, Invitrogen (A21202)

Validation

Validation of antibodies:
All primary antibodies used in this study were validated by manufacturers and are frequently used in publications. Validation statement for each antibody is provided on the manufacturer's website. All secondary antibodies are used in many publications. The detailed information is listed as follows:
Primary antibodies:
• Anti-Nuclei Antibody [Clone 235-1 (HuNu), Millipore Sigma, Cat# MAB1281, Host# Mouse, Dilution# 1:100]:

Validation by immunohistochemistry-immunofluorescence staining of human neural stem cell transplanted into rat brain and SH-SY5Y cells.
Citation from manufacturer is listed at: https://www.emdmillipore.com/US/en/product/Anti-Nuclei-Antibody-clone-235-1,MM_NF-MAB1281

• Anti-Green Fluorescent Protein (GFP) [Rockland, Cat# 600-101-215 Host# Goat, Dilution# 1:1000]:
Validation by immunohistochemistry-immunofluorescence staining of E5.5 Hex-GFP transgenic mouse embryo and neurons generated through Sf-1: Cre mice crossed to the Z/EG reporter line.
Citation from manufacturer is listed at: https://www.rockland.com/categories/primary-antibodies/gfp-antibody-600-101-215/GetProductDataSheet/?code=600-101-215

• Anti-Alpha Smooth Muscle Actin (α-SMA/ACTA2) [Abcam, Cat# ab5694, Host# Rabbit, Dilution# 1:500]:
Validation by immunohistochemistry-immunofluorescence staining of paraffin embedded mouse intestine and mesentery and Pancreatic vessel imaging in the intact adult mouse pancreas.
Citation from manufacturer is listed at: https://www.abcam.com/alpha-smooth-muscle-actin-antibody-ab5694.html

• Anti-Glial Fibrillary Acidic Protein (GFAP) [Millipore Sigma AB5804, Host# Rabbit, Dilution# 1:1000]:
Validation by immunohistochemistry-immunofluorescence staining of astrocytes in human brain and rat cerebral cortex neurons.
Citation from manufacturer is listed at: https://www.emdmillipore.com/US/en/product/Anti-Glial-Fibrillary-Acidic-Protein-GFAP-Antibody,MM_NF-AB5804#documentation

• Anti-Neurofilament-L (C28e10) [Cell Signaling, Cat# 2837, Host# Rabbit, Dilution# 1:100]:
Validation by immunohistochemistry-immunofluorescence staining of paraffin-embedded mouse brain and normal rat cerebellum.
Citation from manufacturer is listed at: https://www.cellsignal.com/datasheet.jsp?productId=2837&images=1

• Anti-E-Cadherin (24E10) [Cell Signaling, Cat# 3195, Host# Rabbit, Dilution# 1:400]:
Validation by immunohistochemistry-immunofluorescence staining of paraffin-embedded human lung carcinoma, mouse prostate, human metastatic adenocarcinoma in lymph node, and MCF7 cells.
Citation from manufacturer is listed at: https://www.cellsignal.com/datasheet.jsp?productId=3195&images=1

• Anti-N-Cadherin [Abcam, Cat# AB18203, Host# Rabbit, Dilution# 1:1000]:
Validation by immunohistochemistry-immunofluorescence staining of human ovarian cancer tissue, human melanoma xenograft mouse model, and human embryonic stem cells differentiated into mesoderm.
Citation from manufacturer is listed at: https://www.abcam.com/n-cadherin-antibody-intercellular-junction-marker-ab18203.html

• Anti-Nestin [Novus, Cat# NB100-1604, Host# Chicken, Dilution# 1:800]:
Validation by immunohistochemistry-immunofluorescence staining of 3T3 cells, adult dentate gyrus of the hippocampal formation, and A7R5 neuroblastoma cells.
Citation from manufacturer is listed at: https://www.novusbio.com/PDFs/NB100-1604.pdf

• Anti Sox2 [Invitrogen, Cat# MA1-014, Host# Mouse, Dilution# 1:200]:
Validation by immunohistochemistry-immunofluorescence staining of H9 embryonic stem cells grown for a few days on Matrigel-coated chamber slides, H9 embryonic stem cells grown for a few days on Matrigel-coated chamber slides, and human neural stem cells derived from PD-3 iPSCs.
Citation from manufacturer is listed at: https://www.thermofisher.com/order/genome-database/dataSheetPdf?producttype=antibody&productsubtype=antibody_primary&productId=MA1-014&version=223

• Anti-Iba1 Antibody [EPR16588] [Abcam, Cat# ab178846, Host# Rabbit, Dilution# 1:500]:
Validation by immunohistochemistry-immunofluorescence staining of rat normal brain, human normal hippocampus, and mouse microglia cells.
Citation from manufacturer is listed at: https://www.abcam.com/iba1-antibody-epr16588-ab178846.html

• Anti-Brdu (Bu20a) [Cell Signaling, Cat# 5292, Host# Mouse, Dilution# 1:200]:
Validation by immunohistochemistry-immunofluorescence staining of HeLa cells and Jurkat cells.
Citation from manufacturer is listed at: https://www.cellsignal.com/datasheet.jsp?productId=5292&images=1

• Anti-Collagen I antibody [Abcam, Cat# ab34710, Host# Rabbit, Dilution# 1:500]:
Validation by Western blot analysis of Col1a1 expression in human collagen.
Citation from manufacturer is listed at: https://www.abcam.com/collagen-i-antibody-ab34710.html

• Anti-Collagen I antibody [ThermoFischer Scientific, Cat# PA5-29569, Host# Rabbit, Dilution# 1:500]:
Validation by Western blot analysis of Col1a1 expression in SK-N-SH whole cell extracts in comparison to HT-29 cells.
Citation from manufacturer is listed at: https://www.thermofisher.com/order/genome-database/dataSheetPdf?producttype=antibody&productsubtype=antibody_primary&productId=PA5-29569&version=223

• Anti-Fibronectin [Abcam, Cat# ab2413, Host# Rabbit, Dilution# 1:1000]:
Validation by immunohistochemistry-immunofluorescence staining of human kidney tissue and HeLa cells.
Citation from manufacturer is listed at: https://www.abcam.com/fibronectin-antibody-ab2413.html

• Anti-CD68 [Abcam, Cat# ab125212, Host# Rabbit, Dilution# 1:1000]:

Validation by immunohistochemistry-immunofluorescence staining of rat liver tissue, mouse spleen, and mouse liver tissue.
Citation from manufacturer is listed at: https://www.abcam.com/cd68-antibody-ab125212.html

• Anti-CD31 [Cell Signaling, Cat# 77699, Host# Rabbit, Dilution# 1:100]:
Validation by immunohistochemistry staining of mouse liver, A2058 xenograft, mouse kidney and mouse small intestine.
Citation from manufacturer is listed at: https://www.cellsignal.com/datasheet.jsp?productId=77699&images=1

• Anti-Survivin [Cell Signaling, Cat# 2808, Host# Rabbit, Dilution# 1:500]:
Validation by immunohistochemistry-immunofluorescence staining of human lung carcinoma showing nuclear localization, human colon carcinoma, human pituitary adenoma, human transitional epithelial carcinoma of the bladder and HeLa cells.
Citation from manufacturer is listed at: https://www.cellsignal.com/datasheet.jsp?productId=2808&images=1

• Anti-PCNA [Cell Signaling, Cat# 2586, Host# Mouse, Dilution# 1:1000]:
Validation by immunohistochemistry-immunofluorescence staining of human breast carcinoma, showing nuclear localization, human colon carcinoma, human non-Hodgkin's lymphoma, and NCI-H460 cells.
Citation from manufacturer is listed at: https://www.cellsignal.com/datasheet.jsp?productId=2586&images=1

• Anti-Cleaved Caspase 3 [Cell Signaling, Cat# 9661, Host# Rabbit, Dilution# 1:400]:
Validation by immunohistochemistry-immunofluorescence staining of human tonsil, showing cytoplasmic and perinuclear localization in apoptotic cells and HT-29 cells, untreated (left) or Staurosporine #9953 treated.
Citation from manufacturer is listed at: https://www.cellsignal.com/datasheet.jsp?productId=9661&images=1

• Anti-TdTomato [SICGEN, Cat# AB8181-200, Host# Goat, Dilution# 1:200]:
Validation by immunofluorescence staining of 293HEK cells transfected with cDNA.
Citation from manufacturer is listed at: https://www.sicgen.pt/product/tdtomato-polyclonal-antibody_1_135

• Anti-Glial Fibrillary Acidic Protein (GFAP) [EMD Millipore, Cat# AB5541, Host# Chicken, Dilution# 1:500]:
Validation by immunohistochemistry-immunofluorescence staining of human brain, rat neonatal forebrain cells, and Hippocamppal derived rat glial cells.
Citation from manufacturer is listed at: https://www.emdmillipore.com/US/en/product/Anti-Glial-Fibrillary-Acidic-Protein-Antibody,MM_NF-AB5541

• Anti-Green Fluorescent Protein (GFP) [Abcam, Cat# AB290, Host# Rabbit, Dilution# 1:500]:
Validation by immunohistochemistry-immunofluorescence staining of bone marrow-derived infiltrating cells in the stromal tissue of gastric intraepithelial tumor, mouse brain tissue, dog hearts, GFP-transfected NIH3T3 cells, and U2OS cells expressing TRF2-GFP fusion protein.
Citation from manufacturer is listed at: https://www.abcam.com/gfp-antibody-ab290.html

Secondary antibodies:

• Polyclonal Goat Anti-Rabbit Immunoglobulins/Biotin [Agilent, Cat# E043201-6, Host# Goat, Dilution# 1:1000]:
Citation from manufacturer is listed at: https://www.agilent.com/store/en_US/Prod-E043201-6/E043201-6

• Polyclonal Goat Anti-Mouse Immunoglobulins, Biotinylated, Secondary Antibody [Agilent, Cat# E0433, Host# Goat, Dilution# 1:1000]:
Citation from manufacturer is listed at: https://www.agilent.com/cs/library/msds/SDS480_NAEnglish.pdf

• Goat Anti-Chicken IgY antibody (H+L), Biotinylated, Secondary Antibody [Vector Laboratories, Cat# BA-9010-1.5, Host# Goat, Dilution# 1:1000]:
Citation from manufacturer is listed at: https://vectorlabs.com/products/antibodies/biotinylated-goat-anti-chicken-igy
• Goat anti-Chicken IgY (H+L) Secondary Antibody, Alexa Fluor 555 [Invitrogen, Cat# A-21437, Host# Goat, Dilution# 1:1000]:
Citation from manufacturer is listed at: https://www.thermofisher.com/antibody/product/Goat-anti-Chicken-IgY-H-L-Secondary-Antibody-Polyclonal/A-21437

• Donkey anti-Rabbit IgG (H+L) Secondary Antibody, Alexa Fluor Plus 488 [Invitrogen, Cat# A32790, Host# Donkey, Dilution# 1:1000]:
Citation from manufacturer is listed at: https://www.thermofisher.com/antibody/product/A32790.html

• Donkey anti-Goat IgG (H+L) Secondary Antibody Alexa Fluor Plus 488 [Invitrogen, Cat# A32814, Host# Donkey, Dilution# 1:1000]:
Citation from manufacturer is listed at: https://www.thermofisher.com/antibody/product/Donkey-anti-Goat-IgG-H-L-Highly-Cross-Adsorbed-Secondary-Antibody-Polyclonal/A32814

• Donkey anti-Rabbit IgG (H+L) Secondary Antibody, Alexa Fluor Plus 555 [Invitrogen, Cat# A32794, Host# Donkey, Dilution# 1:1000]:
Citation from manufacturer is listed at: https://www.thermofisher.com/antibody/product/Donkey-anti-Rabbit-IgG-H-L-Highly-Cross-Adsorbed-Secondary-Antibody-Polyclonal/A32794

• Donkey anti-Goat IgG (H+L) Secondary Antibody, Alexa Fluor Plus 647 [Invitrogen, Cat# A-21447, Host# Donkey, Dilution# 1:1000]:
Citation from manufacturer is listed at: https://www.thermofisher.com/antibody/product/Goat-anti-Chicken-IgY-H-L-Secondary-Antibody-Polyclonal/A-21447

• Donkey anti-Chicken IgY (IgG) (H+L) Secondary Antibody, Alexa Fluor 594 [Jackson ImmunoResearch Labs, Cat# 703-585-155, Host#

Donkey, Dilution# 1:1000:
Citation from manufacturer is listed at: https://www.jacksonimmuno.com/catalog/products/703-585-155

• Donkey anti-Mouse IgG (H+L) Secondary Antibody, Alexa Fluor Plus 488 [Invitrogen, Cat# A-21202, Host# Donkey, Dilution# 1:1000]:
Citation from manufacturer is listed at: https://www.thermofisher.com/antibody/product/Donkey-anti-Mouse-IgG-H-L-Highly-Cross-Adsorbed-Secondary-Antibody-Polyclonal/A-21202

Eukaryotic cell lines

Policy information about cell lines

Cell line source(s)	Mouse NPA, NPD, NPAshCol1A1, NPDshCol1A1 neurospheres were derived from genetically engineered tumor using the Sleeping Beauty (SB) transposase system as previously described [1-4]. Mouse GL26 glioma cells were generated by Sugiura K and obtained from the frozen stock maintained by the National Cancer Institute (Bethesda, MD) [5]. MSP-12 human glioma cell lines were provided by Christine Brown, City of Hope, and SJGBM2 human glioma cells were provided by Children's Oncology Group (COG) Repository, Health Science Center, Texas Tech University.<br>References:<br>1. Calinescu, A. A. et al. Transposon mediated integration of plasmid DNA into the subventricular zone of neonatal mice to generate novel models of glioblastoma. Journal of visualized experiments : JoVE, doi:10.3791/52443 (2015).<br>2. Comba, A. et al. Fyn tyrosine kinase, a downstream target of receptor tyrosine kinases, modulates antiglioma immune responses. Neuro-oncology 22, 806-818 (2020).<br>3. Koschmann, C. et al. ATRX loss promotes tumor growth and impairs nonhomologous end joining DNA repair in glioma. Sci Transl Med 8, 328ra328, doi:10.1126/scitranslmed.aac8228 (2016).<br>4. Núñez, F. J. et al. IDH1-R132H acts as a tumor suppressor in glioma via epigenetic up-regulation of the DNA damage response. 11, eaaq1427, doi:10.1126/scitranslmed.aaq1427 %J Science Translational Medicine (2019).<br>5. Brat, D. J. et al. Comprehensive, Integrative Genomic Analysis of Diffuse Lower-Grade Gliomas. N Engl J Med 372, 2481-2498, doi:10.1056/NEJMoa1402121 (2015).
Authentication	The cell lines used in the study were not authenticated.
Mycoplasma contamination	All cell lines were tested for mycoplasma contamination. Cell lines used in the present study were mycoplasma free.
Commonly misidentified lines<br>(See ICLAC register)	We didn't use any commonly misidentified cell lines in the study.

Animals and other organisms

Policy information about studies involving animals; ARRIVE guidelines recommended for reporting animal research

Laboratory animals	Six to eight week old female C57BL/6 mice and 6-8 week old male and female B6.129(Cg)-Gt(ROSA)26Sortm4(ACTB-tdTomato-EGFP)Luo/J- transgenic mice were purchased from Jackson Laboratory (Bar Harbor, ME). Six to eight week old male NOD.Cg-Prkdcscid Il2rgtm1Wjl/SzJ (i.e., NOD-scid IL2Rgnull or NSG) mice were also purchased from Jackson Laboratory (Bar Harbor, ME), and were housed in a pathogen-free, humidity and temperature-controlled vivarium on a 12:12 hour light: dark cycle with free access to food and water at the University of Michigan.
Wild animals	No wild animals were used.
Field-collected samples	No field-collected samples were used.
Ethics oversight	All in vivo experiments were conducted according to the guidelines approved by the Institutional Animal Care and Use Committee (IACUC) at the University of Michigan protocols PRO00009599, PRO00009578 and PRO00009551. All animals were housed in an AAALAC accredited animal facility.

Note that full information on the approval of the study protocol must also be provided in the manuscript.

Human research participants

Policy information about studies involving human research participants

Population characteristics	Patient-derived primary glioma samples from the University of Michigan Medical School Hospital were used to analyze the existence of oncostreams and Col1a1 expression. Blood samples were not collected.
Recruitment	*Describe how participants were recruited. Outline any potential self-selection bias or other biases that may be present and how these are likely to impact results.*
Ethics oversight	All patients gave informed consent for collection of tissue collection under Institutional Review Board–approved protocols (CNS Tissue Registry: HUM00057130 and HUM00024610) at the University of Michigan.

Note that full information on the approval of the study protocol must also be provided in the manuscript.
